# Behavior of Trapped Molecules in Lantern-Like Carcerand Superphanes

**DOI:** 10.1021/acs.jcim.4c01040

**Published:** 2024-10-11

**Authors:** Andrzej Eilmes, Mirosław Jabłoński

**Affiliations:** † Faculty of Chemistry, Jagiellonian University in Kraków, Gronostajowa 2, PL-30 387 Krakow, Poland; ‡ Faculty of Chemistry, Nicolaus Copernicus University in Toruń, Gagarina 7, PL-87 100 Torun, Poland

## Abstract

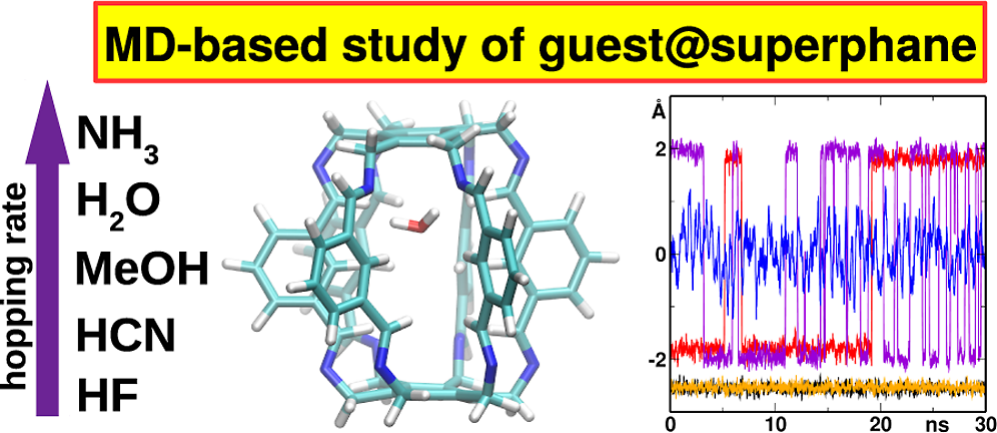

Superphanes are a group of organic molecules from the cyclophane family. They are characterized by the presence of two parallel benzene rings joined together by six bridges. If these bridges are sufficiently long, the superphane cavity can be large enough to trap small molecules or ions. Using ab initio (time scale of 80 ps) and classical (up to 200 ns) molecular dynamics (MD) methods, we study the behavior of five fundamental molecules (M = H_2_O, NH_3_, HF, HCN, MeOH) encapsulated inside the experimentally reported lantern-like superphane and its two derivatives featuring slightly modified side bridges. The main focus is studying the dynamics of hydrogen bonds between the trapped M molecule and the imino nitrogen atoms of the side chains of the host superphane. The length of the N···H hydrogen bond increases in the following order: HF < HCN < H_2_O < MeOH < NH_3_. The mobility of the trapped molecule and its preferred position inside the superphane cage depend not only on the type of this molecule but also largely on the in/out conformational arrangement of the imino nitrogens in the side chains of the superphane. Their inward-pointing positions allow the formation of strong N···H hydrogen bonds. For this reason, these nitrogens are the preferred sites of interaction. The mobility of the molecules and their residence times on each side of the superphane have been explained by referring to the symmetry and conformation of the given superphane cage. All force field MD simulations have shown that the encapsulated molecule remained inside the superphane cage for 200 ns without any escape event to the outside. Moreover, our simulations based on some endohedral complexes in the water box also showed no exchange event. Thus, the superphanes we study are true carcerand molecules. We attribute this property to the hydrophobic side chains and their pinwheel arrangement, which makes the side walls of the studied superphanes fairly impenetrable to small molecules.

## Introduction

**Figure 1:**
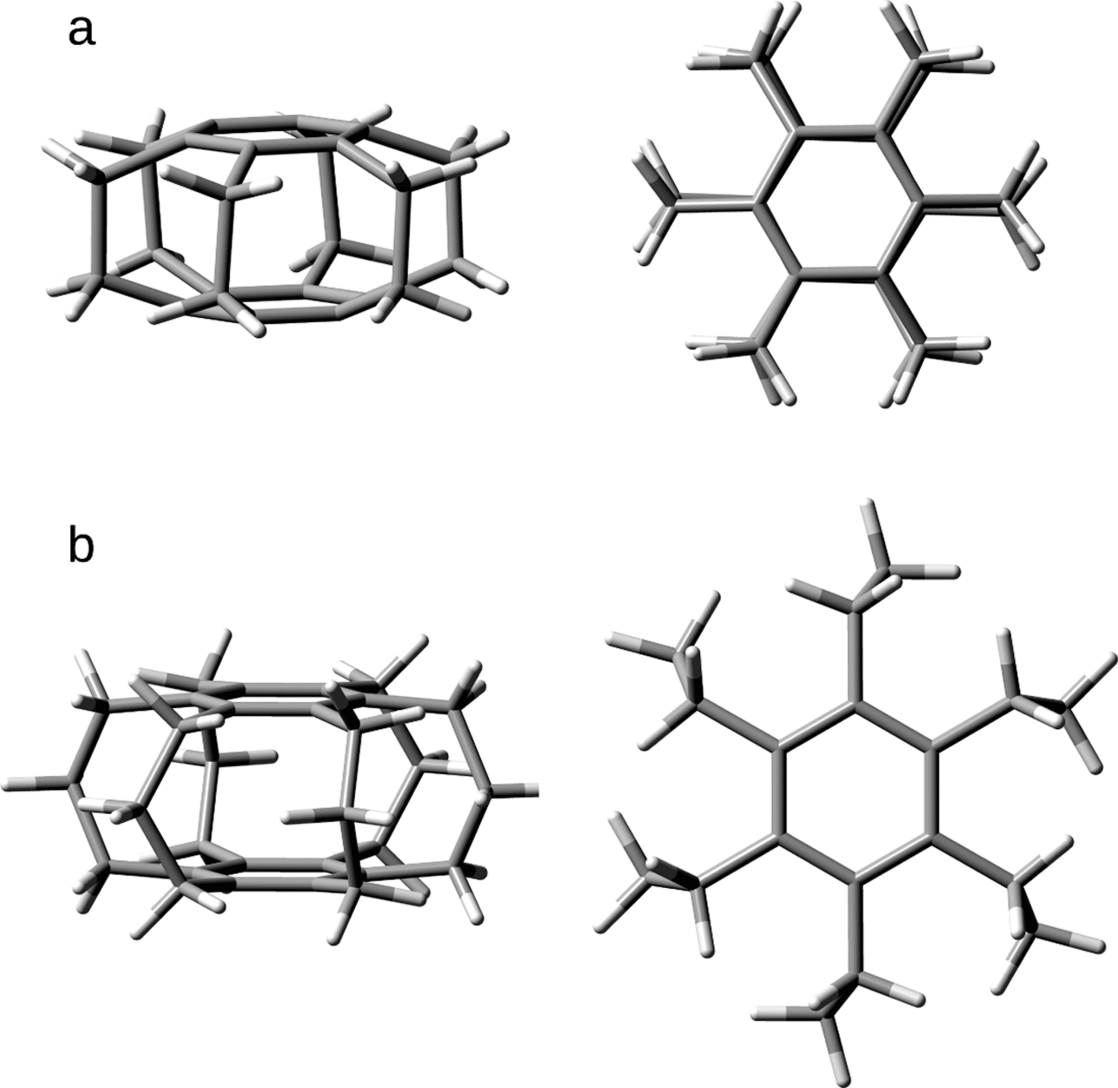
Side and top views of the [2_6_](1,2,3,4,5,6) (a) and [3_6_](1,2,3,4,5,6) (b) superphanes.

Superphanes are aesthetically beautiful organic molecules reminiscent of six-blade pinwheels, Chinese lanterns, pumpkins, or barrels. Its simplest representative, [2.2.2.2.2.2](1,2,3,4,5,6)cyclophane (or shortly [2_6_](1,2,3,4,5,6)cyclophane), consists of two parallel arranged benzene rings joined together by six ethylene bridges (Figure 1a). It was first synthesized by Boekelheide in 1979,^1^ then by Hopf in 1983,^2^ and again by Boekelheide in 1984.^3^ This superphane is characterized by high strain resulting from the forced proximity of both benzene rings, which gives it specific physicochemical properties.^4−8^ However, the larger representative, [3.3.3.3.3.3](1,2,3,4,5,6)cyclophane (or shortly [3_6_](1,2,3,4,5,6)cyclophane), having six trimethylene bridges (Figure 1b),^9^ is devoid of such strain. This allows for the occurrence of a conformational phenomenon, related to flipping the directions of bending of trimethylene bridges.^10−15^ In the energetically most stable form with *C*_6h_ symmetry^11,12,15^ all blades are bent in the same direction.

Importantly, superphanes can be seen as starting compounds for a very wide group of cyclophanes, containing only 2 to 5 bridges connecting benzene rings.^16−29^ Moreover, these bridges may have different lengths, may contain different substituents, and even the benzene rings themselves may be replaced with other ring systems.^30−33^ The variety of possible changes is so large that cyclophanes actually constitute a very important group of organic compounds.^16^

**Figure 2:**
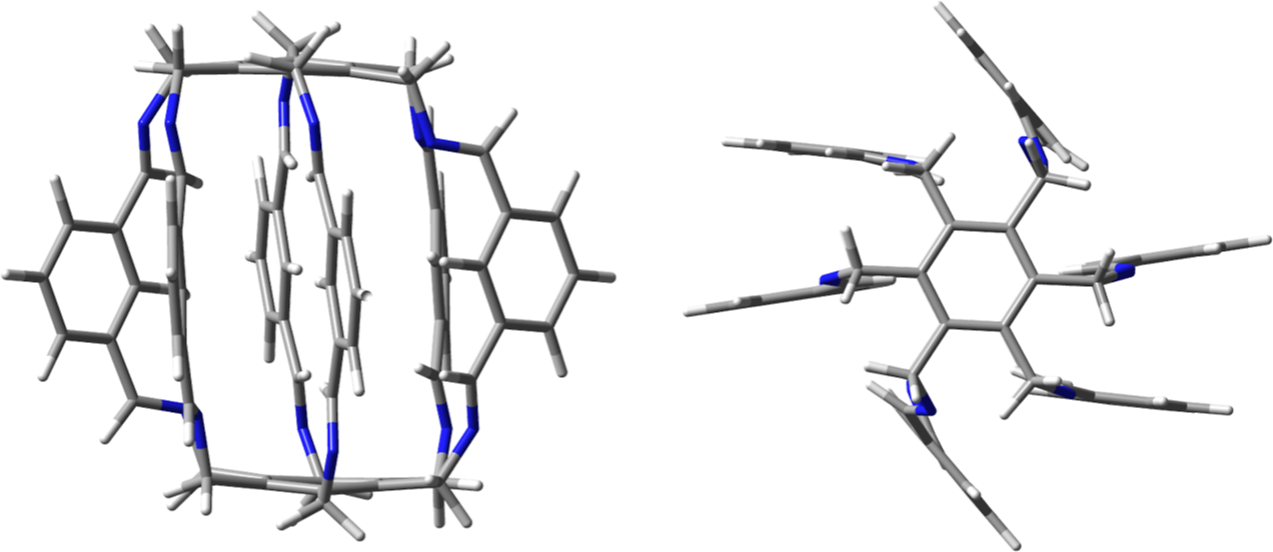
Side and top views of the lantern-like superphane **1** synthesized by Qing He’s group.^42^

Of course, the cage structure of superphanes sparks imagination about encapsulating various chemical entities inside them. The possibility of trapping single noble gas atoms by superphanes [2_6_](1,2,3,4,5,6) and [3_6_](1,2,3,4,5,6) was recently investigated by one of us (M. J.)^7,8,15^ in the context of the interpretation of so-called counterintuitive bond paths^34−38^ related to Bader’s Quantum Theory of Atoms in Molecules (QTAIM).^39−41^ Of course, it is expected that the trapping ability of superphane should increase significantly as its size increases, and therefore mainly by the lengthening of the side chains connecting both benzene rings.^15^ Moreover, this trapping can be further facilitated by the use of numerous binding sites or charged groups in the side chains. Indeed, quite recently this technique was used by Qing He’s group, who synthesized the large-sized lantern-like superphane **1** (Figure 2). Moreover, it has been shown that **1** is able to trap a water dimer inside itself.^42^ This dimer was stabilized by numerous hydrogen bonds involving side benzene rings and protons from imine groups.

Shortly thereafter, this group demonstrated the ability to encapsulate small molecules and ions by various similar superphanes.^43−46^ The trapped species included (2Cl^–^·H_2_O) and MeOH,^43^ ReO_4_^–^, DMSO, and (H_2_O·MeOH),^44^ H_2_PO_4_^–^ and AsO_4_^3–^,^45^ or I_2_ and I_3_^–^.^46^ The trapped species were found interacting with multiple binding sites via hydrogen bonds. As has often been emphasized,^42−46^ the obtained superphanes are characterized by extremely high selectivity toward trapped species over many other types of competing ions. Moreover, the obtained carceplexes show high thermal stability in a wide range of pH values. Therefore, the obtained superphanes may be extremely important in terms of, for example, removing highly toxic ions (e.g., AsO_4_^3–^) from industrial wastewater.

**Figure 3:**
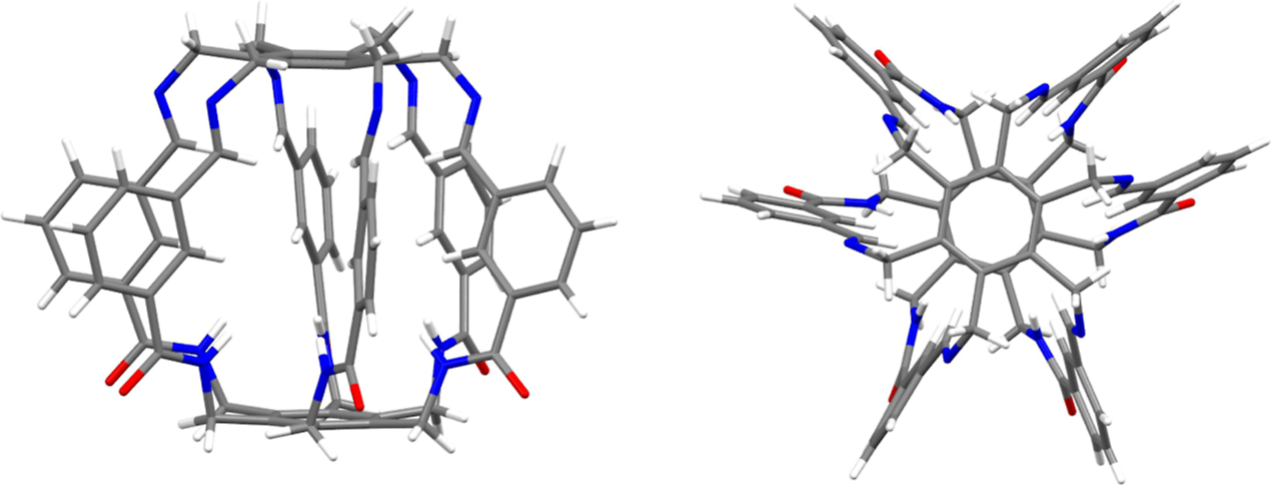
Side and top views of the superphane synthesized by Badjić et al.^47^

Similar research on the selective ion-catching abilities of superphanes is also conducted by the group of Badjić.^47,48^ Importantly, their barrel-shaped hexapodal superphane (Figure 3) is able to easily bind tetrahedral oxyanions such as SO_4_^2–^ or HPO_4_^–^.^47^ High selectivity and different ion trapping times result from the specific slotted structure of the side surface of the superphane. It turns out that the adaptation of the appropriate conformation by the side spokes of the carcerand superphane molecule facilitates the accommodation of the trapped ion.

It is also worth mentioning the recent articles by Oh et al.^49^ and Zhao et al.^50^ Although their carcerands are not superphanes as they contain only three rather than six side chains, they are shown to feature high recognition toward tetrahedral oxyanions such as H_2_PO_4_^–^ and SO_4_^2–^. These two articles prove that the ability to capture and bind ions does not necessarily depend on the total number of side chains limiting the cavity of the cyclophane molecule but on the number of binding sites that can form intermolecular hydrogen bonds. Moreover, these are also good examples demonstrating the key role of intermolecular hydrogen bonds in the recognition and binding of various chemical species.^42−50^

More recently, Qing He’s group also reported the excellent ability of a solution of superphane (very similar to the one in Figure 2 but having 12 secondary amine units in place of the imine ones and 6 pyridyl rings instead of benzene ones) in chloroform to adsorb CO_2_ from various ultradilute sources, viz. flue gas, exhaled gas, or indoor air.^51^ Moreover, importantly, the adsorbed CO_2_ could be easily released under (sub)ambient conditions by triggering mechanical power (magnetic stirring) and its concentration increased from 6% up to 83%.

**Figure 4:**
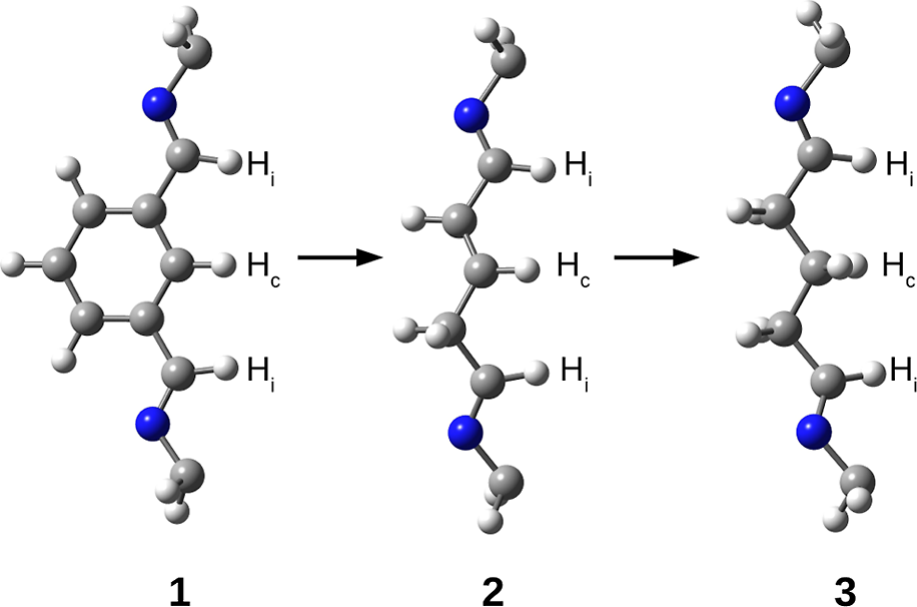
Side chains in superphanes **1**, **2**, and **3**. Hydrogen atoms participating in hydrogen bonds with the guest molecule are labeled as follows: H_i_—imino H atom, H_c_—central H atom.

Recently, one of us (M. J.) has investigated the motifs and energies of hydrogen bonds and other intermolecular interactions between the binding sites of superphane **1** and five fundamental molecules M (M = H_2_O, NH_3_, HF, HCN, MeOH) trapped in it.^52^ An additional goal of previous studies was to investigate the influence of the type of superphane side chain (Figure 4) on the structural motifs and energies of the intermolecular interactions present in the resulting M@**2** and M@**3** endohedral complexes.

**Figure 5:**
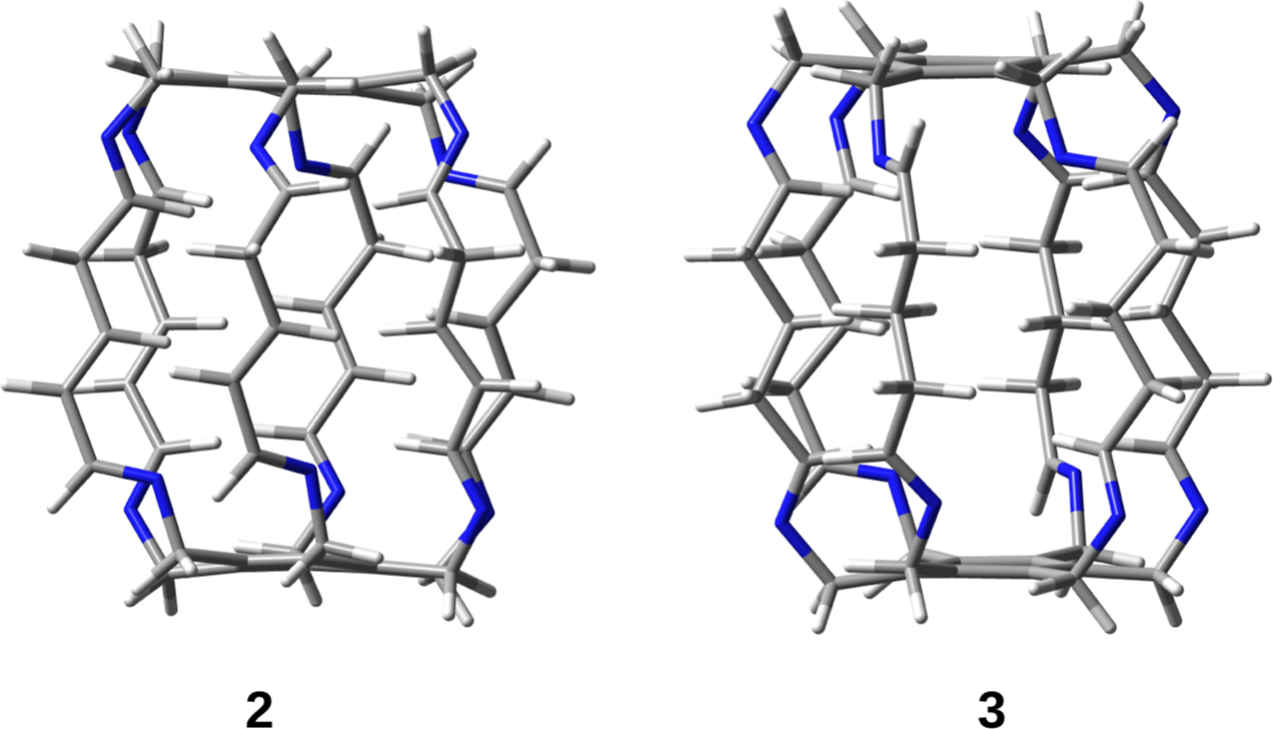
Superphanes **2** and **3**.

As can be seen from Figure 4, superphanes **2** and **3**, created from **1**, are characterized by the presence of structurally simplified side chains connecting opposite benzene rings. Namely, the **1** → **2** transformation involves replacing the benzene ring in the side chains with the –CH=CH–CH_2_– fragment having a double bond, while the **2** → **3** replacement involves saturating this bond, i.e., introducing the –CH_2_–CH_2_–CH_2_– fragment instead. As a result of these changes to all side chains of superphane **1**, the resulting superphanes **2** and **3** (Figure 5) are characterized by a simplified structure that nevertheless retains all the important binding sites present in superphane **1**.

The aim of the current paper is to study the behavior of M molecules trapped in superphanes **1**, **2** and **3** using molecular dynamics (MD) methods. In particular, the aim of interest is the dynamics of hydrogen bonds formed with the participation of imine nitrogen atoms of the side chains of the considered superphane molecules, as well as the impact of the change **1** → **2** → **3** on these dynamics. We will also investigate how the conformations of the superphane molecule depend on the type of the side chains and how these structural differences affect the mobility of the encapsulated molecule M inside the cage.

## Computational Methodology

Simulations of the evolution of the M@**1**, M@**2**, and M@**3** (M = H_2_O, NH_3_, HF, HCN, MeOH) endohedral complexes performed using MD methods were based on their previously obtained structures.^52^ These were determined at the ωB97X-D/6-31G(d) level of theory, i.e., using the ωB97X-D exchange–correlation functional^53^ of Density Functional Theory^54,55^ and the 6-31G(d) basis set.^56,57^ In the current work, ab initio molecular dynamics (AIMD) simulations were performed with a time step of 1 fs at the ωB97X/6-31G(d) level at *T* = 350 K, employing the Langevin thermostat. TeraChem v. 1.9 package^58^ running on Tesla V100 GPUs was used for AIMD. Because of high computational cost, only empty superphane cages **1–3**, H_2_O and HF molecules in all superphanes **1–3**, and NH_3_, HCN, and MeOH in superphane **1** were studied via ab initio dynamics. For each structure, 80 ps of the trajectory were computed with frames saved at each step.

Classical MD was applied to empty superphanes **1–3** and all 15 complexes M@**1**–M@**3**. Force field (FF) parameters for bonded interactions and Lennard-Jones potential for superphane molecules were based on the OPLS-AA parametrization.^59^ Values of some missing dihedral angles were adapted from the MM3 field.^60^ Partial atomic charges were obtained from the fit to the electrostatic potential, calculated at the ωB97X-D/6-31G(d) level using Gaussian 09 software.^61^ The TIP3P potential^62^ was used for H_2_O. For other molecules OPLS-AA parameters were used, with some adjustment of partial charges in order to improve the agreement between N–H radial distribution functions calculated from AIMD and FF based MD (FF MD) trajectories. Tinker v. 7.1 package^63^ was used for all FF MD simulations. For endohedral complexes in vacuum, 200 ns long trajectories were obtained with a time step of 1 fs at *T* = 350 K using the Bussi thermostat.^64^ The relatively high temperature was set in order to increase the speed of dynamics in the system. Frames of the trajectory were recorded for further analysis in the 5 ps intervals, except where noted otherwise. Three independent simulations were performed for each system using different seeds for random number generator.

Additional classical simulations were performed for H_2_O@**1** and H_2_O@**2** complexes and empty cages of **1** and **2** carcerands soaked in cubic boxes of 500 TIP3P water molecules. The *NVT* ensemble at *T* = 350 K was used with the size of the periodic box set to the value maintaining the pressure at 1 atm (all structures) or 10 000 atm (empty cages only). The length of trajectories simulated for solvated systems was 80 ns.

## Results and Discussion

### Conformation of Superphane Cages

**Figure 6:**
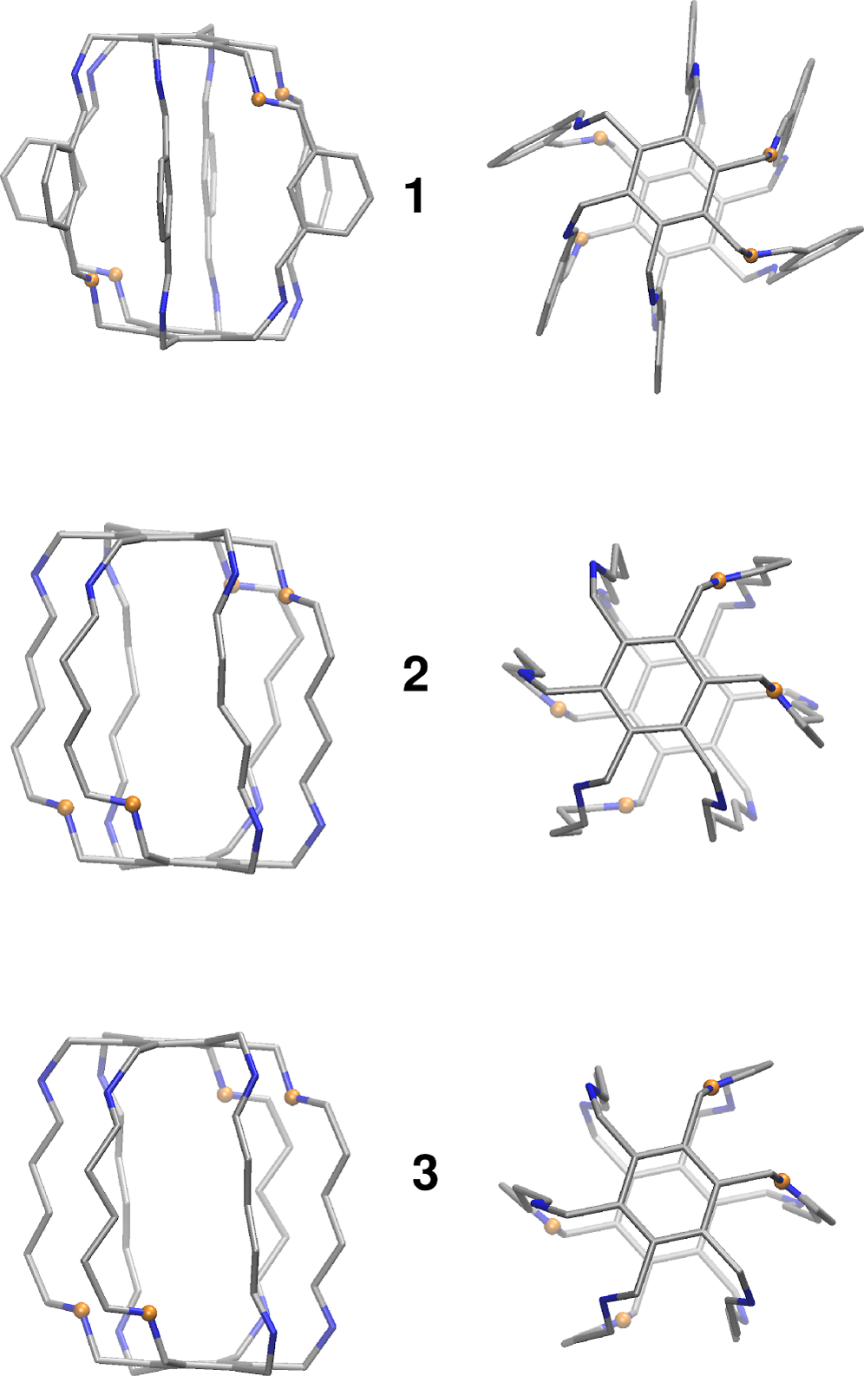
Positions of inward pointing N atoms (marked by orange spheres) in the initial structures of superphanes **1–3**. Hydrogen atoms are not shown.

The initial geometries for MD simulations were taken from the structures of endohedral complexes optimized in a recent quantum-chemical study of intermolecular interactions between guest molecules and superphanes.^52^ Accordingly, conformations at the nitrogen atoms in **1** corresponded to the structure determined experimentally for the crystal,^42^ that is, four out of 12 nitrogen atoms point inside of the cavity of the superphane. The four inward-pointing N atoms are located in pairs at the opposite sides of the carcerand cage, consistent with *C*_i_ symmetry. This arrangement of nitrogen atoms resulted from the presence of hydrogen bonds stabilizing the trapped water dimer.^42^ In the case of **2** and **3** the conformations at N atoms were the same as in **1**; initial structures with inward-pointing N atoms are shown in Figure 6.

Full optimization of the geometry of the considered M@**1**–M@**3** complexes led to positions of the M molecules in which they are stabilized by the presence of hydrogen bonds and other intermolecular interactions involving the inwardly directed nitrogen atoms as well as the imine and central (Figure 4) hydrogen atoms.^52^

**Figure 7:**
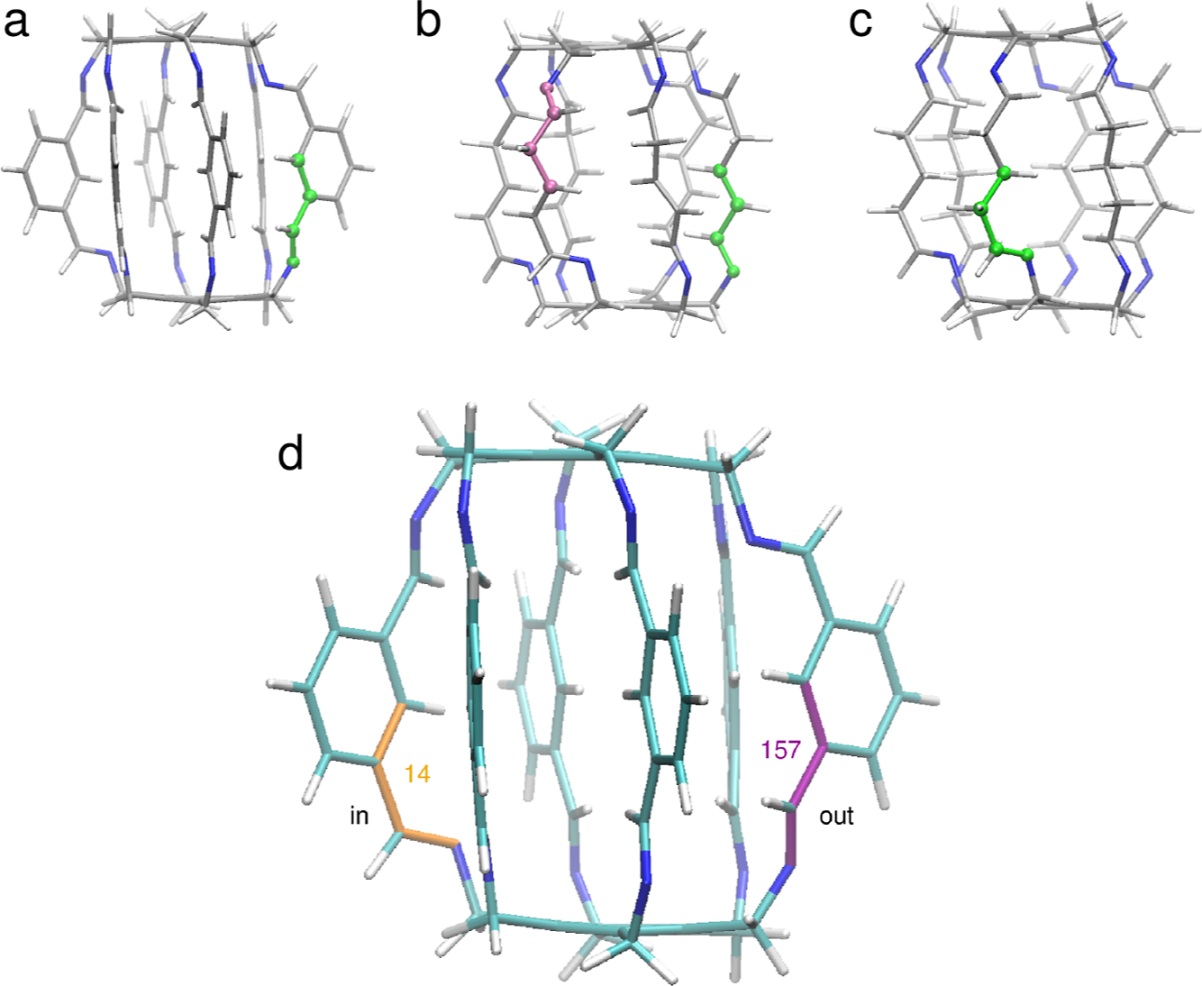
Definitions of C–C–C–N dihedral angles used to trace the conformations at N atoms in superphane **1** (a), superphane **2** (b), and superphane **3** (c); small values of the angle correspond to in conformations, whereas large absolute values indicate out conformations (d).

Conformations at the N atoms in **1** were monitored during MD simulations through the values of the Θ(C_b_–C_b_–C–N) dihedral angle, where C_b_ is the carbon atom from the side-chain benzene ring (Figure 7a). In **2** and **3**, the carbon atoms replacing the C_b_ atoms in the side chains were used in the definition of Θ (Figure 7b,c). Values of Θ close to 0° indicate that the nitrogen atom points inside the cavity, whereas large absolute values of the angle correspond to the nitrogens pointing outside (Figure 7d).

**Figure 8:**
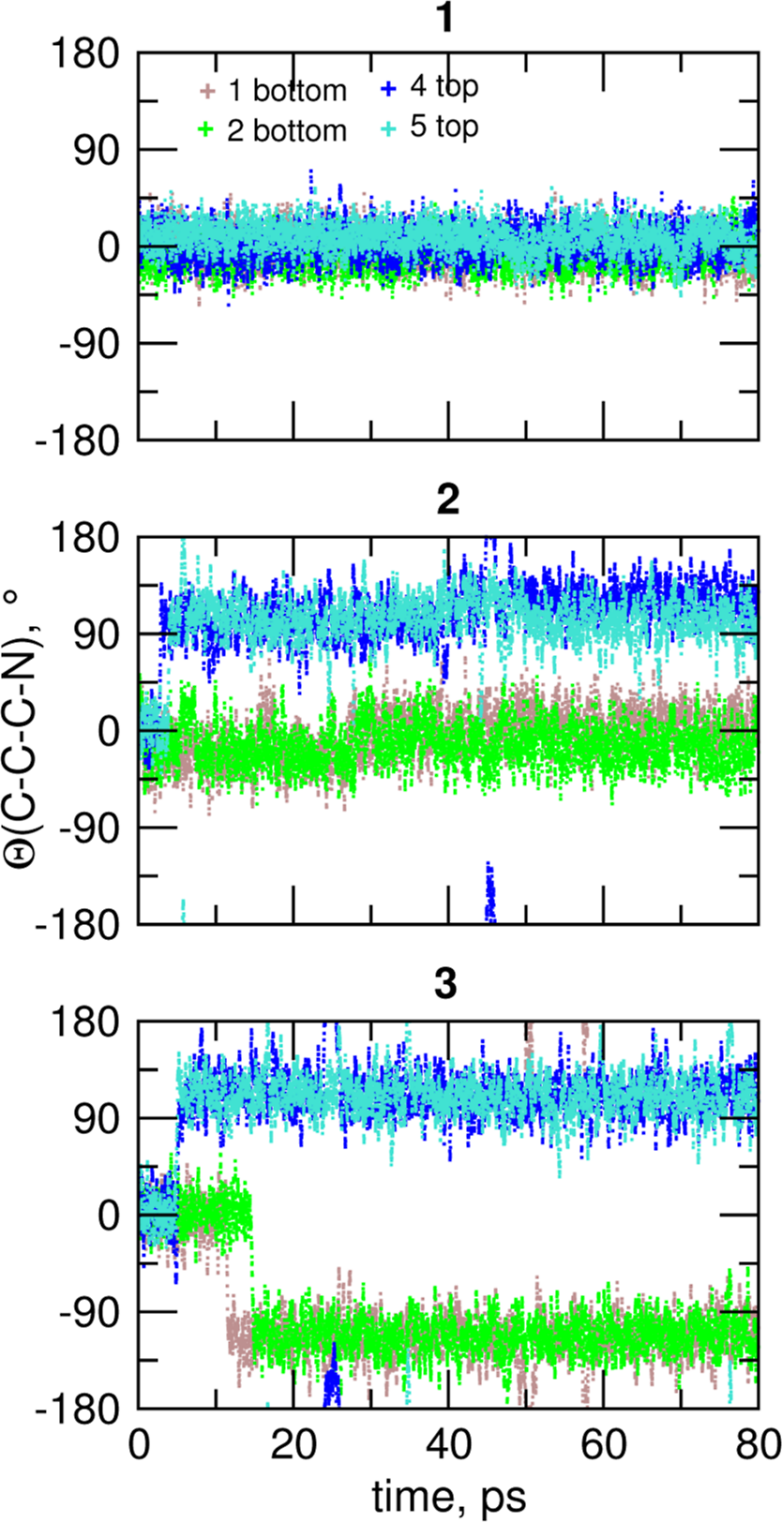
Evolution of conformations at the four nitrogen atoms inward-pointing in the initial structures during the AIMD simulations for empty superphane cages **1–3**. Nitrogen atoms at the bottom and the top of the cage are labeled “b” and “t”, respectively.

In Figure 8 we trace the conformations of the four N atoms pointing inside in the initial structures of empty superphane cages during the AIMD simulations. In the symmetric **1** cage there are always two pairs of inward-pointing N atoms: one at the bottom and one at the top of the cage and this configuration remains unchanged during the simulations. The cage **2** with the –CH=CH–CH_2_– sequence of atoms in the side chain is asymmetric; for the purpose of our analysis we define the bottom of the cage as the side closer to the double bond, whereas the opposite end is the top. The conformation at the pair of inward-pointing N atoms at the bottom is preserved within the time-scale of simulations. Contrarily, within 10 ps, the inward-pointing pair at the opposite end rotates and all N atoms at the top of **2** point outside the cavity. Most likely, this rotation results from the presence of a single C–C bond in the top part of the side chains, while in the bottom part, such rotation is strongly inhibited due to the presence of a conjugated –CH=CH–CH=N– fragment. Finally, the cage **3** is symmetric with all single C–C bonds in the side chains, that is, the same as on the top side of **2**. In this case fast reorientations of N atoms took place at both ends of the cage and after 20 ps all four nitrogens are pointing outside, with similar absolute values of the Θ dihedral angle.

**Figure 9:**
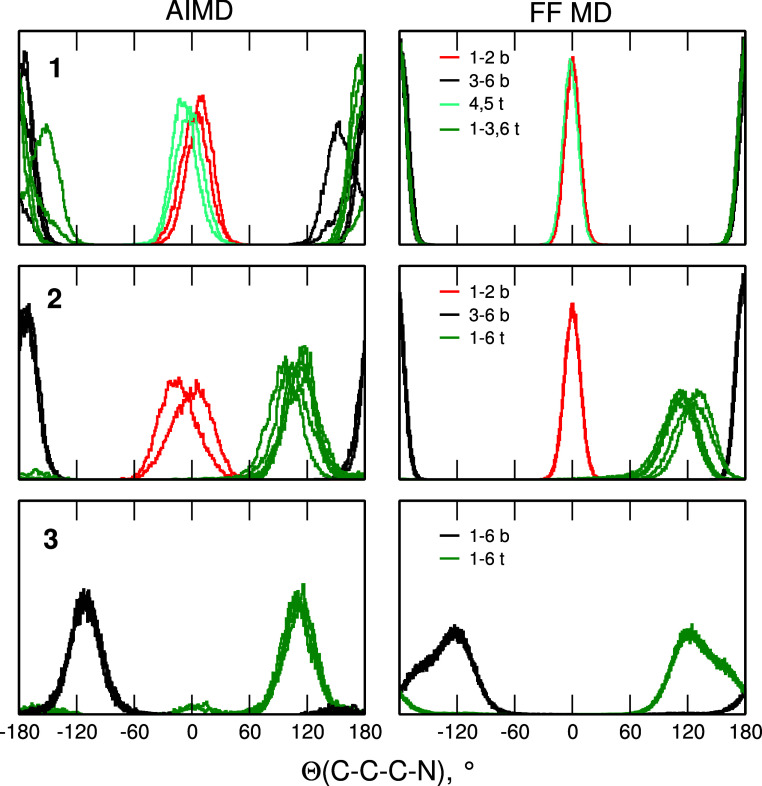
Conformations at the nitrogen atoms in MD simulations for empty superphane cages **1–3**. Each line corresponds to one N atom. Nitrogen atoms at the bottom and the top of the cage are labeled “b” and “t”, respectively.

The above conclusions are supported by Figure 9, in which we compare the distributions of Θ angles in simulations of empty cages **1**, **2** and **3**. The AIMD data were averaged over the last 40 ps of trajectories. In the symmetric **1** cage the two pairs of inward-pointing N atoms at the bottom and at the top of the cage are easily noticeable; the eight other N atoms point outside the cage—the initial configuration was preserved in the course of the simulations. In **2** only the pair of N atoms at the bottom remains in the inward-pointing conformation and all N atoms at the top of **2** point outside the cavity, although the values of Θ at about 120° are smaller than the angles for outward-pointing nitrogens in **1**. In the symmetric **3** superphane there is no difference between the bottom and the top with all 12 N atoms spending most of the time in the outward-pointing conformation. However, in the AIMD data for **3** there is also small probability of dihedral angles close to 0°. These values result from temporary changes of conformation—some N atoms reoriented from outward-pointing to inward-pointing geometry and after few ps returned to the outward-pointing orientation. This effect was not observed in the FF MD results, suggesting that at the level of quantum-chemical calculations, cage **3** is less rigid and some conformational changes are possible even though the all-out structure is preferred. There is a general agreement between AIMD and FF MD results; however, the distribution of angles for inward-pointing N atoms in the FF simulations are narrower, as another indication that the cage is more rigid in the classical simulations. In all classical simulations of empty cages or superphanes with encapsulated molecules behavior of the system was the same in all three simulated replicas.

**Figure 10:**
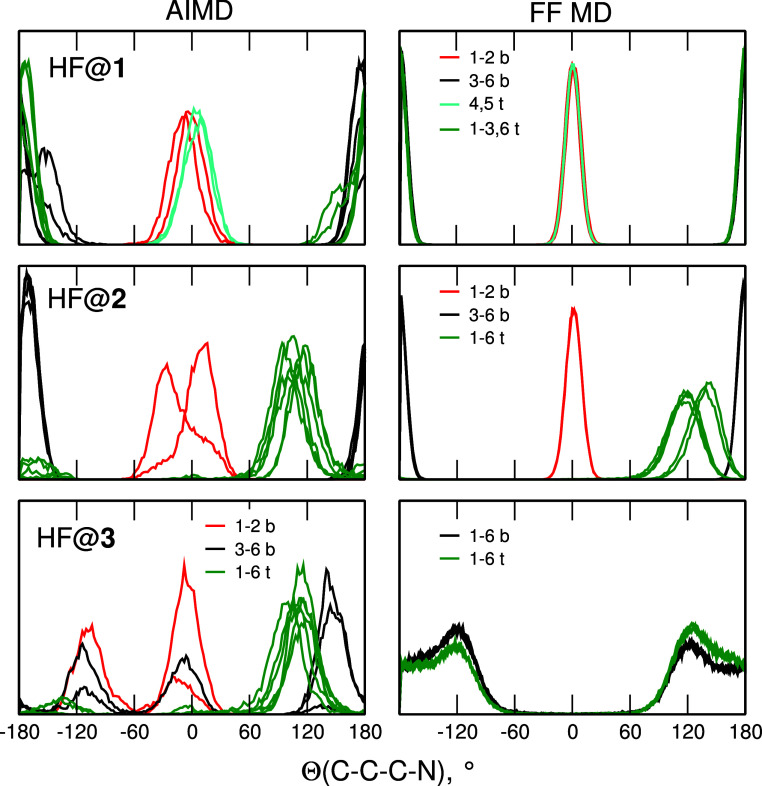
Conformations at the nitrogen atoms in MD simulations for HF encapsulated in **1–3**. Each line corresponds to one N atom. Nitrogen atoms at the bottom and the top of the cage are labeled “b” and “t”, respectively.

For AIMD and FF MD simulations of H_2_O inside **1–3**, changes of conformations at nitrogen atoms were the same as recorded for empty superphane cages (Figures S1 and S2). The same pattern of conformations was observed in the MD simulations for HF inside **1** and **2** (Figure 10). In HF@**1** there is always one inward-pointing pair of N atoms at the bottom and another one at the top of the cage. Conformations at the two nitrogen atoms at the top of **2** changed within the first 40 ps of simulations and all six N atoms at the top of the cage point outside, whereas the pair of atoms at the bottom remains in the inward-pointing geometry, as in the case of H_2_O@**2**. In all structures discussed up to now, results of the FF MD simulations are in agreement with the AIMD data. The case of HF@**3** is different: at the end of the AIMD trajectory there is still at least one N atom (at the bottom) pointing inside the cage. On the other hand, in FF-based simulations, all N atoms at both ends of the superphane molecule are pointing outward, as observed for H_2_O@**3**. Similarity of the AIMD results obtained for the top nitrogens in **2** and all N atoms in **3** together with the observation that the HF molecule is located in the cavity at the inward-pointing N atoms, suggest that the interactions between HF and the nitrogen atoms hinder the conformational changes, preserving the conformation more favorable for hydrogen bonding. The effect is observed in AIMD results only for HF, for which the binding is the strongest of all M molecules.^52^ In the FF MD, even for HF molecule, the cage adopts the conformation with all N atoms pointing outward, because of more rigid cage or/and weaker molecule···cage interactions in classical simulations. Closer inspection of the FF MD results reveals that at several points of the trajectory one of N atoms turns toward the HF molecule and points inside the cavity. Nevertheless, such events are not frequent enough to make the probability of Θ values close to 0° noticeable in Figure 10.

For NH_3_, HCN, and MeOH molecules inside **2** or **3**, the results of both types of MD are the same as for H_2_O@**2** and H_2_O@**3** in Figure S2. This leads us to the conclusion that the preferable conformation of the cage with single C–C bonds in the side chains is with all nitrogen atoms pointing outward and only strong interactions with the encapsulated molecule (the HF case) can stabilize the inward-pointing configuration.

There were no conformational changes observed neither in AIMD nor in FF MD for H_2_O, HCN, and HF in **1** and at both ends of the cage one pair of N atoms is always turned toward the interior of the cavity. There is no reason to expect that this configuration is energetically preferred. Instead, the favorable structure should be rather that with all outward-pointing nitrogens. Therefore, the structures observed for these carceplexes in MD resulted from the initial geometry and lack of conformational changes within the length of the simulations. However, some changes were found in the AIMD simulations for NH_3_@**1** and MeOH@**1**. In the first case, after about 40 ps of simulations, one of the N atoms at the bottom of the cage turned inward the cavity and after another 5 ps such change took place at the opposite N atom at the top. In the case of MeOH@**1** only one such event happened at about 50 ps, therefore the final conformation of the superphane was with three inward-pointing N atoms at the bottom and two at the top side of the cage. It would be tempting to attribute these changes to the presence of the encapsulated molecule, yet two out of three changes took place at the end of the cage at which there was no molecule. Moreover, interactions of NH_3_@**1** are the weakest,^52^ thus rather unlikely to induce the change in the conformation. Nevertheless, **1** is apparently not as rigid as it appeared in the FF MD simulations and there is a possibility to observe the conformational changes in AIMD. Conformational preferences of superphanes **1–3**, dynamics of structural changes and its sensitivity to the interactions with encapsulated molecules are interesting topics themselves, but requiring significant computational effort because of numerous possible configurations. Therefore, we will not delve into more detailed description here, leaving this issue for a future work.

### Dynamics of Encapsulated Molecules

**Figure 11:**
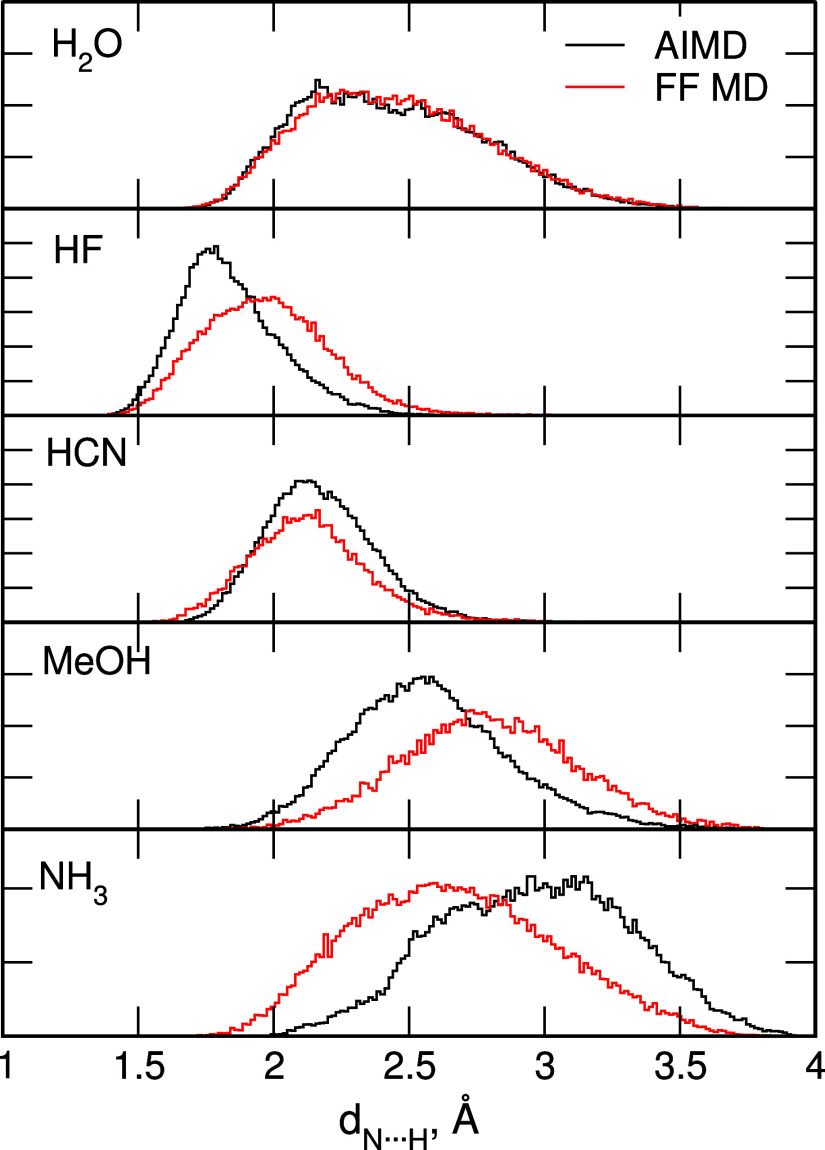
Distributions of N···H distances obtained in MD simulations for M@**1**.

We begin our discussion on the dynamics of molecules M with the analysis of interactions between H atoms of M and the superphane nitrogen atoms. In Figure 11 we compare the distributions of N···H distances obtained in the AIMD and the FF MD simulations for M@**1**. For this purpose, for each frame of the trajectory, we calculated the distance between the H atom involved in the hydrogen bond formation (the sole H atom in HF and HCN, the hydroxyl H in MeOH and any of the H atoms in H_2_O and NH_3_) and the closest N atom of the cage (an example is shown in Figure S3).

The most probable N···H distance in the AIMD trajectories depends on M and increases from 1.75 Å for HF through 2.1–2.2 Å for HCN and H_2_O to 2.5 Å for MeOH, i.e., in the order of hydrogen bonding strength predicted from the quantum chemical calculations.^52^ The distributions for strongly interacting HF and HCN are narrower, whereas those for H_2_O and MeOH are much broader. For the latter two molecules there are long tails at higher distances, in particular for H_2_O, resulting mainly from the increased mobility of these molecules inside the cage. The case of NH_3_@**1** is somewhat different: there is an increase of probability at 2.5–2.6 Å, as expected for the most weakly bound molecule,^52^ but the main maximum is observed at 3 Å. The NH_3_ molecule in a large part of the AIMD simulations approaches the cage center and its binding is so weak that even within the 80 ps of the recorded trajectory we are able to observe some events when the molecule detaches from the N atom and moves toward the other end of the cage. In the AIMD such jumps were relatively infrequent and the NH_3_ molecule spends most of the time in the bottom half of the cage, but within the 200 ns of the FF MD simulations, probabilities of finding the molecule at either end of the cage are approximately equal.

The trends observed for the N···H distances in Figure 11 generally agree between the AIMD and the FF MD simulations. The shape of distributions for H_2_O and HCN and the positions of the maxima are fairly well reproduced. For HF and MeOH, the maxima in the FF MD are wider and shifted to slightly longer distances, indicating somewhat weaker binding effect in classical dynamics. In the FF MD simulations for NH_3_@**1** the maximum of the distribution is at distances shorter than in the AIMD data and its position at about 2.5 Å coincides with the distance at which there is an increase in the AIMD-calculated probability. We relate this distance to the N···H interactions between the N atom of the cage and the ammonia hydrogen, and therefore the length of this hydrogen bond seems to be reproduced. In the AIMD simulations, the NH_3_ molecule often moves toward the center of the cage, and this behavior seems to be related to the possibility of the H···N interactions involving the N atom of ammonia molecule and the cage hydrogen. Such an effect apparently is underestimated in the FF-based dynamics. Nevertheless, the overall agreement between the AIMD and the FF MD calculated distances as well as conformational behavior of the cages discussed in the preceding section, suggest that the classical MD can be used to study the dynamics of the encapsulated molecule for long times, beyond the time scale attainable in the AIMD simulations.

**Figure 12:**
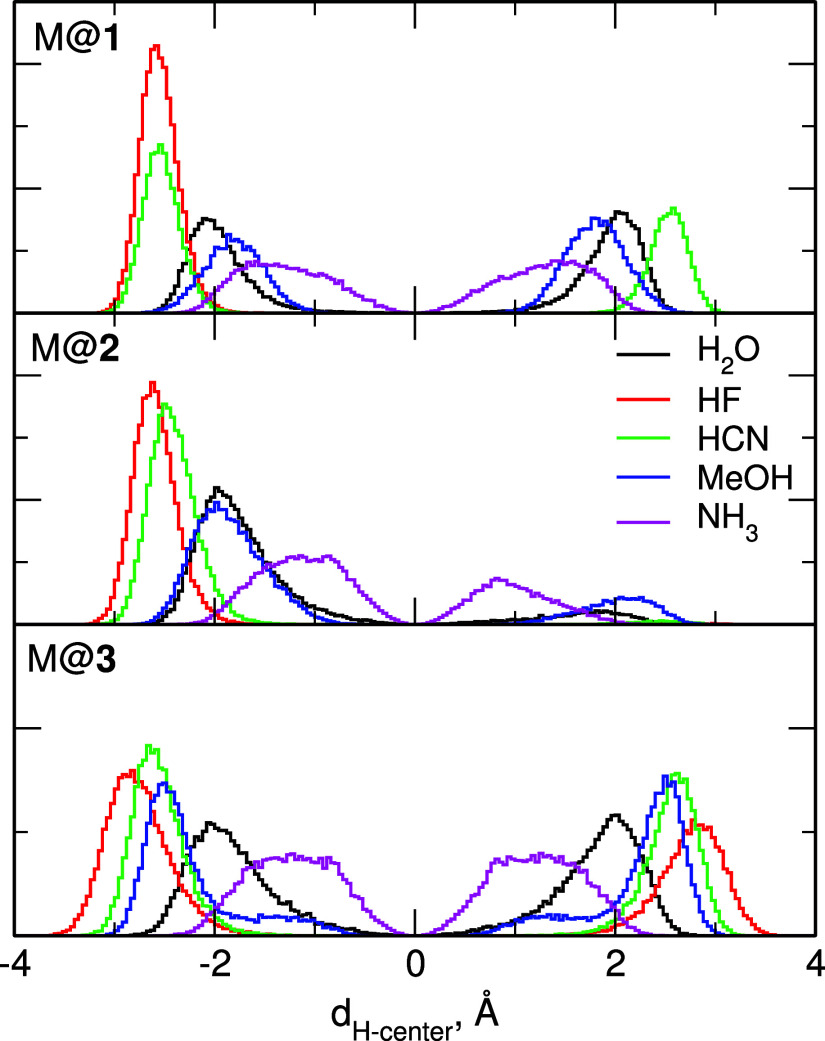
Distributions of the distances between the H atom of M and the center of the superphane cage obtained in the FF MD simulations for M@**1**–M@**3**.

To analyze the movements of M inside the superphane cages we defined the bottom and the top end of the cage as the centers of the benzene rings, and the center of the cage as the midpoint of the line segment connecting the cage ends. Then, using the FF MD trajectories, we calculated the distance *d*_H-center_ between the center of the cage and the H atom of the molecule M farthest (if more than one) from the cage center (Figure S4). In Figure 12 we displayed distributions of these distances; negative and positive values indicate that the H atom is closer to the bottom or to the top of the cage, respectively.

In superphane **1** the distributions for H_2_O, NH_3_, and MeOH are symmetric—the molecules were jumping between the ends of the cage and spent on average equal time at either end. On the other hand, the HF molecule remained at the bottom of the cage during all 200 ns of the simulation. The plot for HCN@**1** is somewhat misleading: the maxima at both ends are similar, suggesting several jumps of the molecule, but in fact, only one such event happened, approximately at halftime of the simulation. Therefore, HCN behaved rather like HF, and in **1** it was confined to the starting position. All the distributions obtained for **2** are asymmetric. Both HF and HCN remained at the bottom of the cage practically for the whole length of the simulation. The other molecules were jumping between the ends, but spent significantly more time at the bottom; the difference is the smallest for the most weakly bound NH_3_. Finally, in the cage **3** the distributions are symmetric for all molecules M, which move inside the cage with no preference toward binding at either end. We can note in Figure 12 that in all cages, the H atoms from the linear and strongly interacting HF and HCN molecules are closest to the cage end, whereas the most probable locations of the weakly bound NH_3_ are closer to the center of the cage.

**Figure 13:**
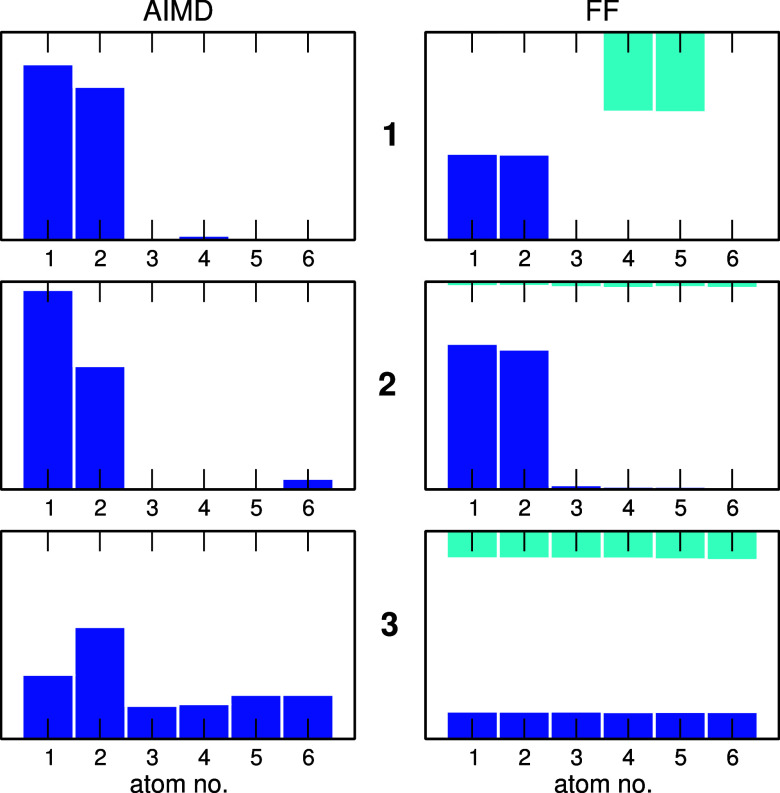
Probabilities of interaction with a water hydrogen atom for individual nitrogen atoms at the bottom (blue) or the top (cyan) of the **1–3** superphane cages.

Mobility of the encapsulated molecule and the preferred location inside the cage can be related to the conformation of superphane molecule. To that end we produced statistics of N atoms of the superphane interacting with H atoms of M. The results are shown in Figure 13 for the H_2_O molecule. In the AIMD simulations the molecule remains at the bottom of the cage. It is readily seen that in **1** and **2** it interacts almost exclusively with N atoms no. 1 and 2, that is, the atoms in an inward-pointing conformation. On the other hand, the **3** cage changed the geometry within the length of the AIMD simulations, so that at the end, all N atoms point outside the cage. Accordingly, the H_2_O molecule interacts with all nitrogen atoms and the increased probability at the atoms no. 1 and 2 is the remainder of the initial structure of the cage.

The FF MD results, with many jumps between the cage ends and therefore improved statistics, further corroborate these observations. In the symmetric **1** superphane, the water molecule interacts (with an equal probability) only with two pairs of inward-pointing N atoms (no. 1 and 2 at the bottom and no. 4 and 5 at the top). In the asymmetric **2** there is only one pair of nitrogen atoms pointing inward the cavity at the bottom of the cage and the H_2_O molecule spends almost all the time close to one of these atoms. The probability of interaction with a nitrogen atom at the top is very small and it is approximately equal for all these outward-pointing atoms. Finally, the superphane **3** is symmetric, with all N atoms in an outward-pointing conformation and the probability of N···H interaction is equal for all 12 nitrogen atoms in this superphane. From the data shown in Figure 13 we can conclude that the conformations at the nitrogen atoms determine the most probable location of the encapsulated molecule inside the cage. The inward-pointing N atoms allow for shorter N···H distances and stronger hydrogen bonds, therefore are the preferred interaction sites.

**Figure 14:**
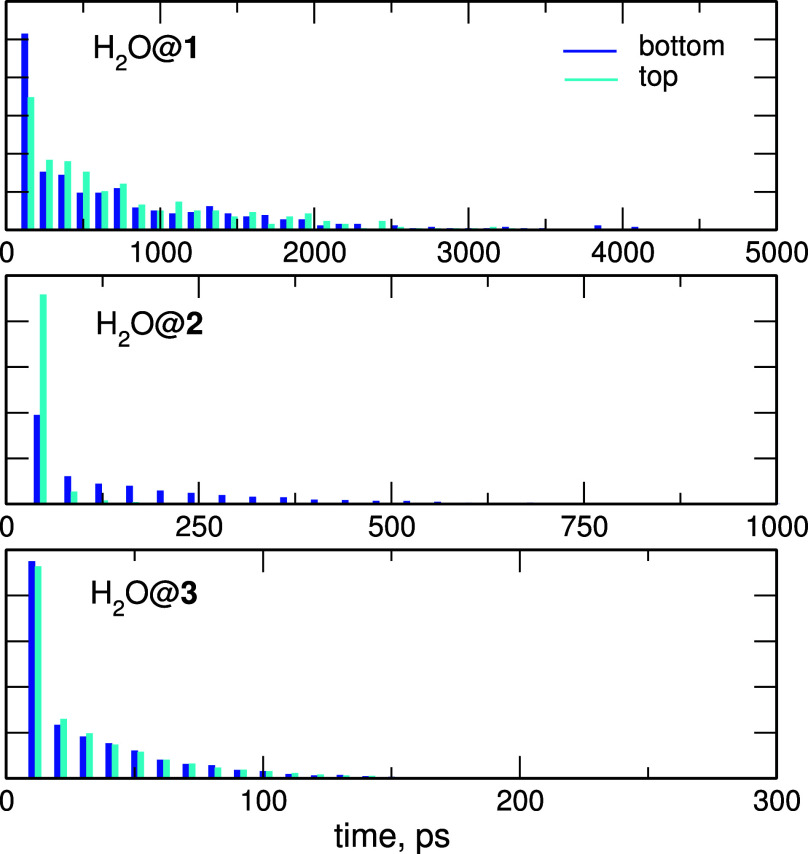
Statistics of residence times for H_2_O in the cages **1–3** obtained from the FF MD simulations. Note the scale difference between panels.

It is natural to ask how these structural differences between the superphanes will affect the mobility of M and the mean time between jumps. To analyze this issue, we calculated the time intervals after which the molecule M moves from one-half of the cage to the other. Sample distributions of such residence times at both ends of cages **1–3** are shown in Figure 14 for the H_2_O molecule.

As readily seen, residence times decrease in the order **1** > **2** > **3**, showing that in **1** the bound water molecule stays on one side of the superphane cage the longest before moving to the other side, while its mobility in **3** is the highest. For **1** and **3**, there is not much difference between the residence times at the bottom and the top, as expected for the symmetric cages. In superphane **2**, however, there is a significant increase in the probability of short times at the top of the cage, whereas the distribution for the bottom end has a tail at the long-time side. In this asymmetric cavity the water molecule spends most of the time at the inward-pointing N atoms at the bottom, thus the residence times confirm the observation made from Figure 13.

For an easy comparison between different molecules and the cages we computed the average residence times, wherever possible. The results are collected in Table 1. For three systems no jumps or only one were observed, prohibiting the calculations. The estimates for MeOH@**1** are subject to large statistical errors, because only about 20 jumps occurred during the 200 ns of the simulations. Values obtained for NH_3_ are probably underestimated, because the ammonia molecule is located closest to the cage center (cf. Figure 12), and this increases the number of false positive events when only a small change in the position of the molecule moves it to the other half of the cage. Residence times for NH_3_ are very small, therefore for better time resolution we performed additional 40 ns long FF MD simulations with frames of the trajectory saved each 0.1 ps.

**Table 1:** Mean Residence Times of Molecules M at the Bottom and the Top of the **1–3** Superphanes Calculated from the FF MD Simulations^a^

system	*t*_bottom_, ps	*t*_top_, ps
H_2_O@**1**	684	703
HF@**1**		
HCN@**1**		
MeOH@**1**	6890	8220
NH_3_@**1**	9.2	9.3
H_2_O@**2**	142	10
HF@**2**		
HCN@**2**	2387	31
MeOH@**2**	248	57
NH_3_@**2**	2	1
H_2_O@**3**	27	27
HF@**3**	1334	1224
HCN@**3**	510	553
MeOH@**3**	200	198
NH_3_@**3**	4.2	4.1

a Results averaged over three trajectories.

Several trends are easily noticeable in Table 1. In all the cages, mobilities of encapsulated molecules increase in the order HF < HCN < MeOH < H_2_O < NH_3_, reflecting the strength of bonding interactions with the nitrogen atoms.^52^ With a minor exception of NH_3_, residence times for a given M decrease from **1** through **2** to **3**. In both symmetric superphanes **1** and **3** residence times at both ends are approximately equal, whereas in **2** the molecule M is located at the bottom of the cage for significantly longer time intervals than those at the top. The differences between the superphanes can be rationalized based on the conformations of the cages. In **1**, pairs of inward-pointing N atoms are available at both ends of the cage. Therefore, residence times are similar at the top and at the bottom and strong interaction increases the mean time between hops. In **2**, a pair of N atoms in a favorable conformation is located only at the bottom. The molecule M can escape to the top of the cage, but with weaker bonding to the top nitrogen atoms it returns quickly to the bottom, where the hydrogen bond formation is more efficient. Finally, in **3**, there are no inward-pointing N atoms and the interactions are weak at both ends of the cage, leading to more frequent hops with no preference to either end.

As a measure of the strength of the interaction M···superphane we calculated the encapsulation energy *E*_int_, defined as
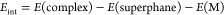
1Technically, *E*_int_ was obtained as the “intermolecular energy” reported by Tinker software, which for two molecules in a nonpolarizable field with pairwise additive energy terms is equivalent to our definition of the encapsulation energy. The *E*_int_ values averaged over 50 ns of the FF MD trajectories are presented in Table 2. For all molecules, interaction with the superphane becomes weaker in the sequence from **1** to **3**, that is, in the same order as the increasing mobility. For a given cage, the binding strength is similar for HF and HCN, decreases for H_2_O and is always the weakest for NH_3_—correlating with the residence times. The exception is MeOH, for which the interaction energies are the most negative (suggesting the strongest binding), yet the MeOH is not the least mobile molecule. However, the *E*_int_ values include interactions of all atoms of the molecule (thus the effect is enlarged for MeOH, being the largest molecule), whereas the strength of N···H interaction is the most important for the mobility. As shown in quantum chemical analysis of MeOH@**n** binding^52^ the sum of individual binding interactions for MeOH is about 5–6 kcal/mol less negative than the encapsulation energy. Therefore, in the case of MeOH, its mobility is not as small as could be expected from the *E*_int_ value.

**Table 2:** Encapsulation Energies (in kcal/mol) of Molecule **M** in Superphane **n** Calculated from the FF MD Simulations^a^

**M**	**1**	**2**	**3**
H_2_O	–12.1	–10.3	–8.0
HF	–15.7	–14.9	–9.8
HCN	–15.2	–14.9	–11.4
MeOH	–16.9	–15.6	–13.4
HH_3_	–7.8	–6.8	–6.0

a Results averaged over three trajectories.

### Tightness of the Cage

In all FF MD simulations, the molecule M remained encapsulated inside the cage for 200 ns, with no events of an escape to the outside. This result is in full agreement with the result previously obtained by Li et al. for the superphane **1** encapsulating a water dimer.^42^ Moreover, other very similar superphanes obtained earlier by the Qing He group also showed long-time and high temperature (ca. 100 °C) integrity of their endohedral complexes with various types of oxyanions (e.g., ReO_4_^–^, H_2_PO_4_^–^, AsO_4_^3–^).^44,45^ With this respect, the superphanes investigated in this work are really carcerand molecules.

**Figure 15:**
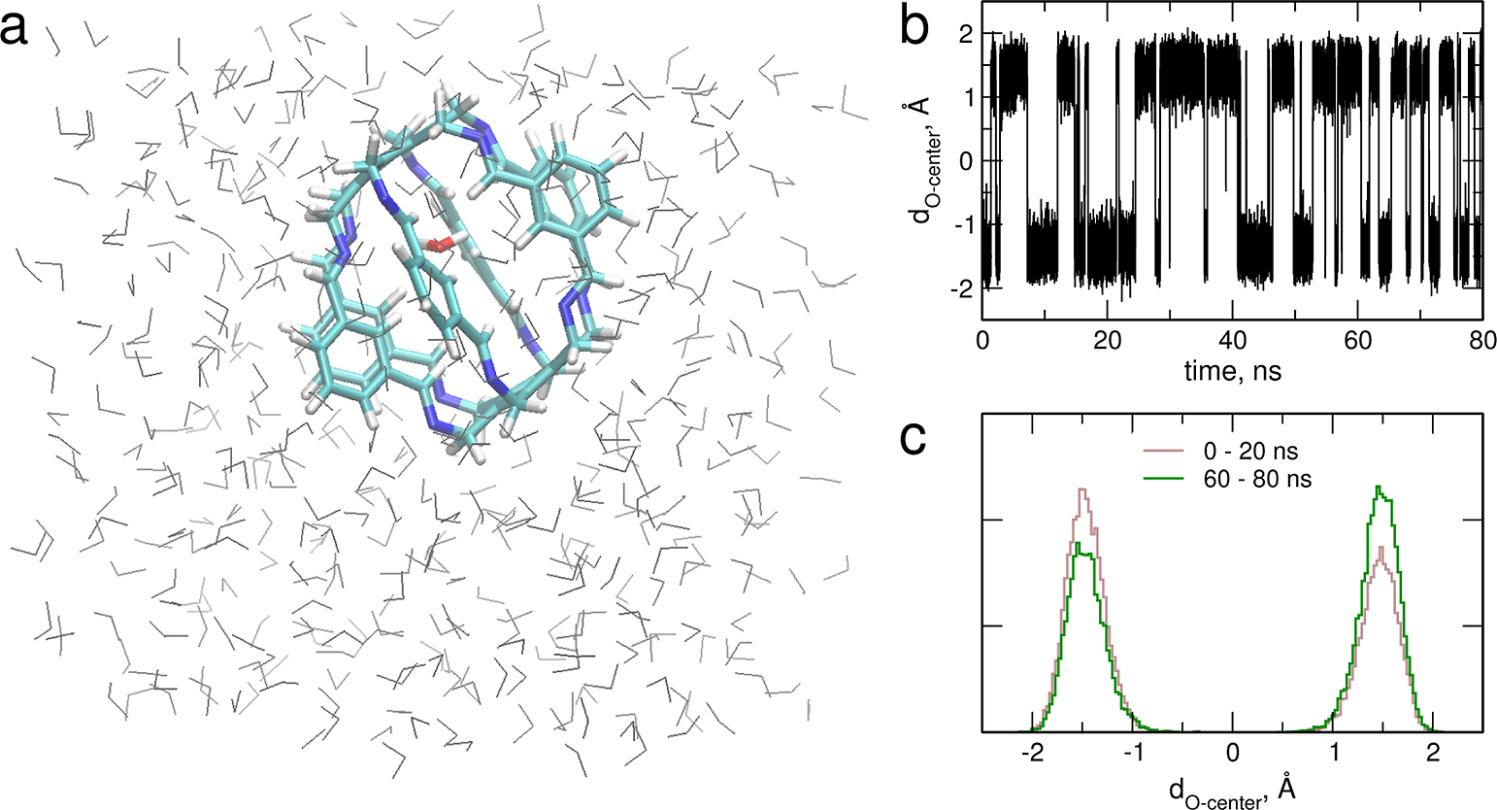
A sample snapshot of the solvated H_2_O@**1** at the end of the FF MD simulations with the encapsulated water molecule shown as sticks and the solvating molecules shown as gray lines (a); evolution of the *d*_O-center_ distance (b); and its distributions at the beginning and at the end of the trajectory (c).

The simulations discussed so far were performed for isolated carceplexes in a vacuum. To check whether the presence of a solvent can facilitate the exchange between the cage and the solution, we computed the MD trajectories for H_2_O@**1** and H_2_O@**2** in the TIP3P water box. The simulations for hydrated **1** and **2** (including those with pressure increased to 10 000 atm) were used to examine the possibility of a reverse process, in which a solvent molecule penetrates into an empty cage; elevated temperature and high pressure were used to increase the mobility of water and the possibility of pushing a water molecule inside the cage. In all cases no exchange event was observed during 80 ns of the simulation. An example is shown in Figure 15 for H_2_O@**1** in a water box. As readily seen, at the end of the 80 ns trajectory the encapsulated water molecule is still located inside the cage. It can also be noted that the cage remains in its initial conformation, with two pairs of inward-pointing N atoms: one is visible at top left and the other at bottom right. The distance *d*_O-center_ between the O atom of the water molecule and the cage center (Figure S5), calculated for each frame of the trajectory, oscillates in the range approximately −2 to 2 Å, confirming that the molecule remained in the cage for the whole length of the simulation. Finally, the distributions of the *d*_O-center_ distances at the first and the last 20 ns of the trajectory are the same, apart from the different heights of the maxima due to slightly different probabilities of finding the molecule at the bottom or at the top of the cage. Therefore, the configuration of the H_2_O@**1** carceplex was unchanged during the simulation. We can conclude that the hydrophobic side chains and their pinwheel arrangement (Figure 2) in the studied superphanes make the walls of the carcerand fairly impenetrable to small molecules.

## Conclusions

Using MD methods, we have studied the behavior of five fundamental molecules M (M = H_2_O, NH_3_, HF, HCN, MeOH) trapped in the cavity of the experimentally obtained lantern-like carcerand superphane **1** and its two derivatives (**2** and **3**) featuring somewhat simplified side chains. The main goal was to investigate the dynamics of hydrogen bonds between the hydrogen atoms of the trapped M molecule and the nitrogen atoms of the imino groups of the side chains of the host superphane molecule and the dependence of these dynamics on the **1** → **2** → **3** structural change. The MD-based simulations have shown that the length of the N···H hydrogen bond depends on the M molecule and increases in the order of its decreasing strength predicted by previous^52^ quantum chemical calculations (i.e., HF > HCN > H_2_O > MeOH > NH_3_). While the distance distributions for HF and HCN are quite narrow, those for H_2_O and MeOH are much wider with long tails for larger distances, showing the greater mobility of these molecules inside the superphane cavity. The weakest bound NH_3_ molecule features the highest mobility.

Importantly, we have shown that there is general agreement between the N···H hydrogen bond lengths determined using AIMD and FF MD, as well as the conformational behavior of superphane cages, which allowed us to conclude that classical MD can be successfully used to describe the evolution of encapsulated molecules for long times, beyond the time scales available in AIMD simulations.

The mobility of the trapped molecule and its preferred position inside the superphane cage depend not only on the type of this molecule but also largely on the conformation of the side chains of the superphane. The most probable positions of the trapped molecule M are determined by the in/out conformational arrangement of the imine nitrogens. Their inward-pointing positions allow the formation of strong N···H hydrogen bonds. For this reason, these nitrogens are the preferred sites of interaction. While in **1** the H_2_O, MeOH and NH_3_ molecules do not show preferences for any side of the superphane, the more strongly bound HF and HCN definitely prefer to be on one side. For asymmetric **2** the obtained position distributions are also asymmetric—the bottom of the cage is preferred by all molecules. In symmetric cage **3**, the position distributions are symmetric for all M molecules, indicating no preference for any of the ends of the cage.

The residence times, i.e. the time intervals after which the molecule M jumps from one-half of the cage to the other, decrease in the following order: **1** > **2** > **3**, showing that in **1** the bound molecule stays on one side of the superphane cage the longest before moving to the other side, while its mobility in **3** is the highest. The mobility of the molecules and their residence times on each side of the superphane have been explained by referring to the symmetry and conformation of the given superphane cage.

A slightly side topic was the study of possible in/out conformational arrangement of nitrogen atoms of imine groups in the side chains, which depends on the symmetry of the side chains and the type of the trapped molecule. For example, the H_2_O@**1** endohedral complex is characterized by two pairs of nitrogens directed to the center of the superphane cage, one on each side, i.e. “at the top” and “bottom”. The H_2_O@**2** complex with asymmetric –CH=CH–CH_2_– fragments in the side chains has only one such pair at the bottom of the cage, while in the H_2_O@**3** complex with symmetrical –CH_2_–CH_2_–CH_2_– fragments in the side chains, all nitrogen atoms after some time of simulation time accept the out position. The inward orientation of nitrogen atoms is favored by the possible presence of a strong hydrogen bond, as, for example, in HF@**3** where the trapped HF molecule is stabilized by N···H–F interaction. Therefore it seems possible that a strongly interacting molecule could become self-trapped in a superphane cage sufficiently flexible to allow for an easy change of in/out nitrogen atoms orientation.

All FF MD simulations have shown that the encapsulated molecule remained inside the superphane cage for 200 ns without any escape event to the outside. Moreover, our simulations based on H_2_O@**1** and H_2_O@**2** in a water box also showed no exchange event. Thus, the superphanes we studied are true carcerand molecules. We attribute this property to the hydrophobic side chains and their pinwheel arrangement, which makes the side walls of the studied superphanes fairly impenetrable to small molecules.

## Supporting Information Available

Figures showing evolution of conformations at the four initially inward pointing N atoms in H_2_O@**n** during the AIMD simulations, conformations at the nitrogen atoms in MD simulations for H_2_O encapsulated in **1–3**, definitions of *d*_N···H_, *d*_H-center_ and *d*_O-center_ distances, and FF parameters and input structures (in the Tinker v7 format) for M@**n** (**n** = **1**, **2**, **3**; M = H_2_O, HF, HCN, NH_3_, MeOH) (PDF)
