# Supplementary material for: Behavior of Trapped Molecules in Lantern-Like Carcerand Superphanes
Source: J Chem Inf Model. 2024 Oct 11;64(20):7925–37. doi: 10.1021/acs.jcim.4c01040 (PMC11523074; doi:10.1021/acs.jcim.4c01040)
Supplement: Supplementary file 1 — ci4c01040_si_001.pdf [file ci4c01040_si_001.pdf]

Supporting Information

Behavior of Trapped Molecules in Lantern-Like Carcerand Superphanes

Andrzej Eilmes† and Mirosław Jabłoński*,‡

†*Faculty of Chemistry, Jagiellonian University in Kraków, Gronostajowa 2, PL–30 387 Krakow, Poland*

‡*Faculty of Chemistry, Nicolaus Copernicus University in Toruń, Gagarina 7, PL–87 100 Torun, Poland*

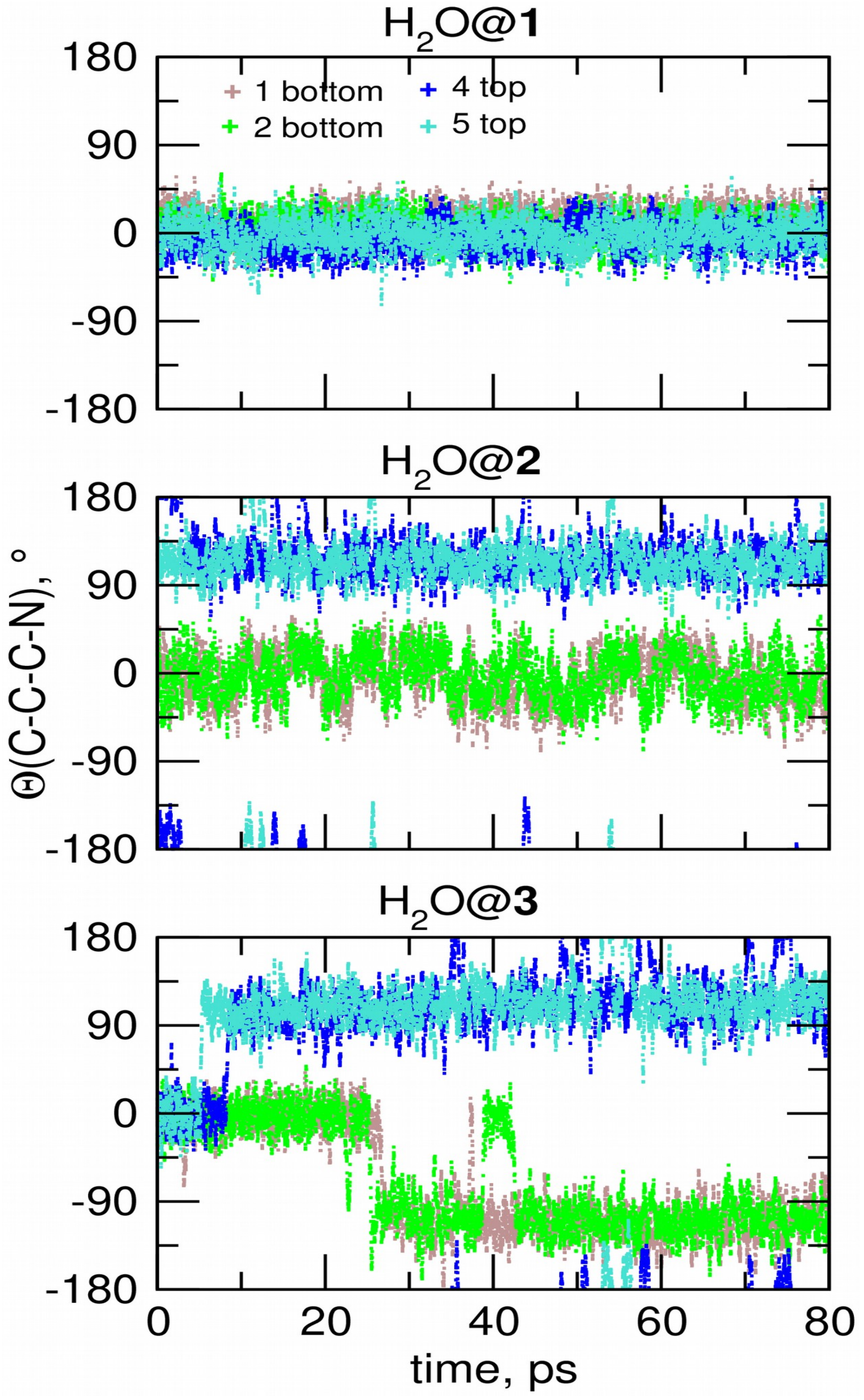

Figure S1: Evolution of conformations at the four initially inward pointing N atoms in H_2O@**n** during the AIMD simulations.

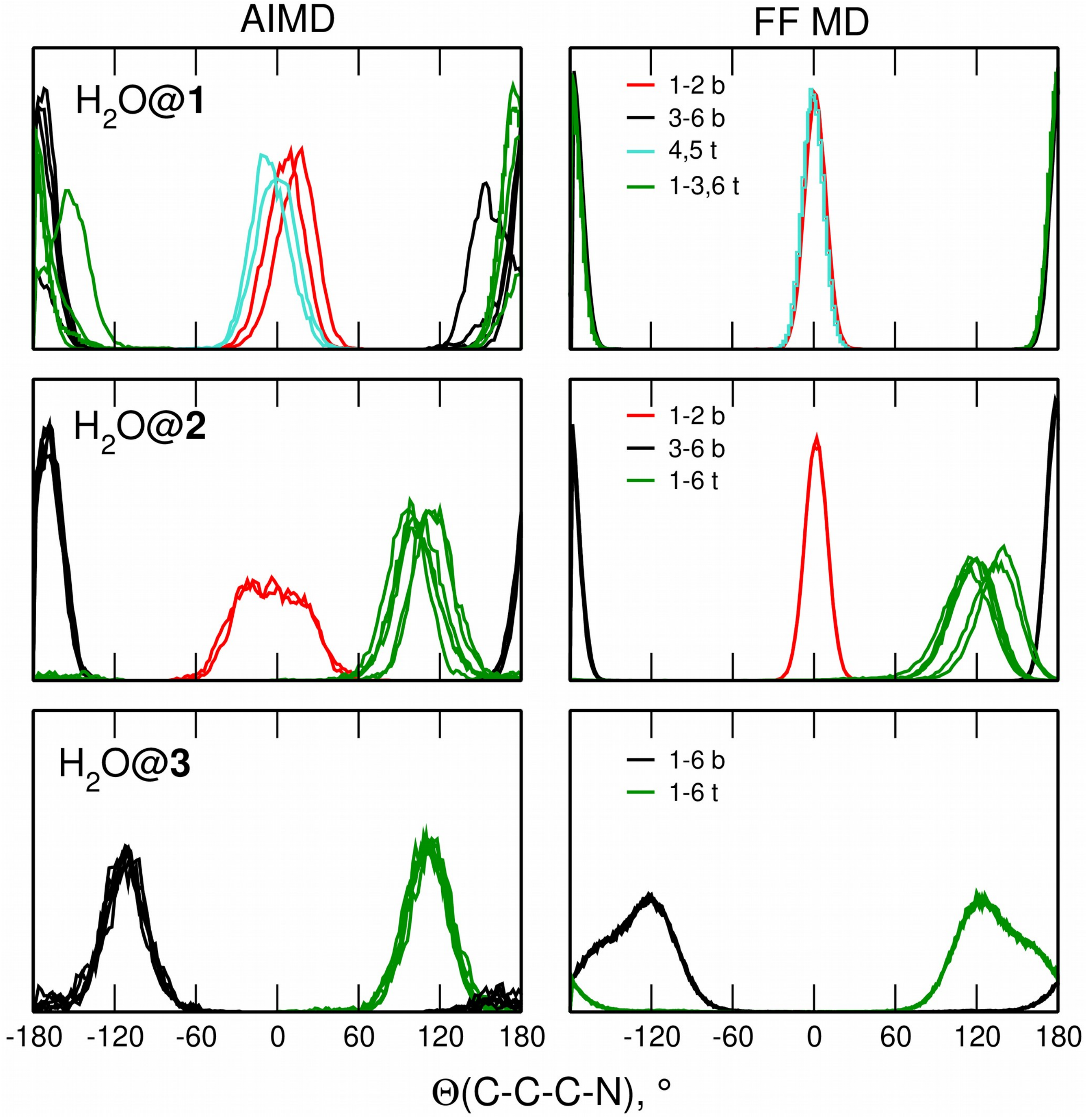

Figure S2: Conformations at the nitrogen atoms in MD simulations for H_2O encapsulated in **1**-**3**. Each line corresponds to one N atom. Nitrogen atoms at the bottom and the top of the cage are labeled “b” and “t”, respectively.

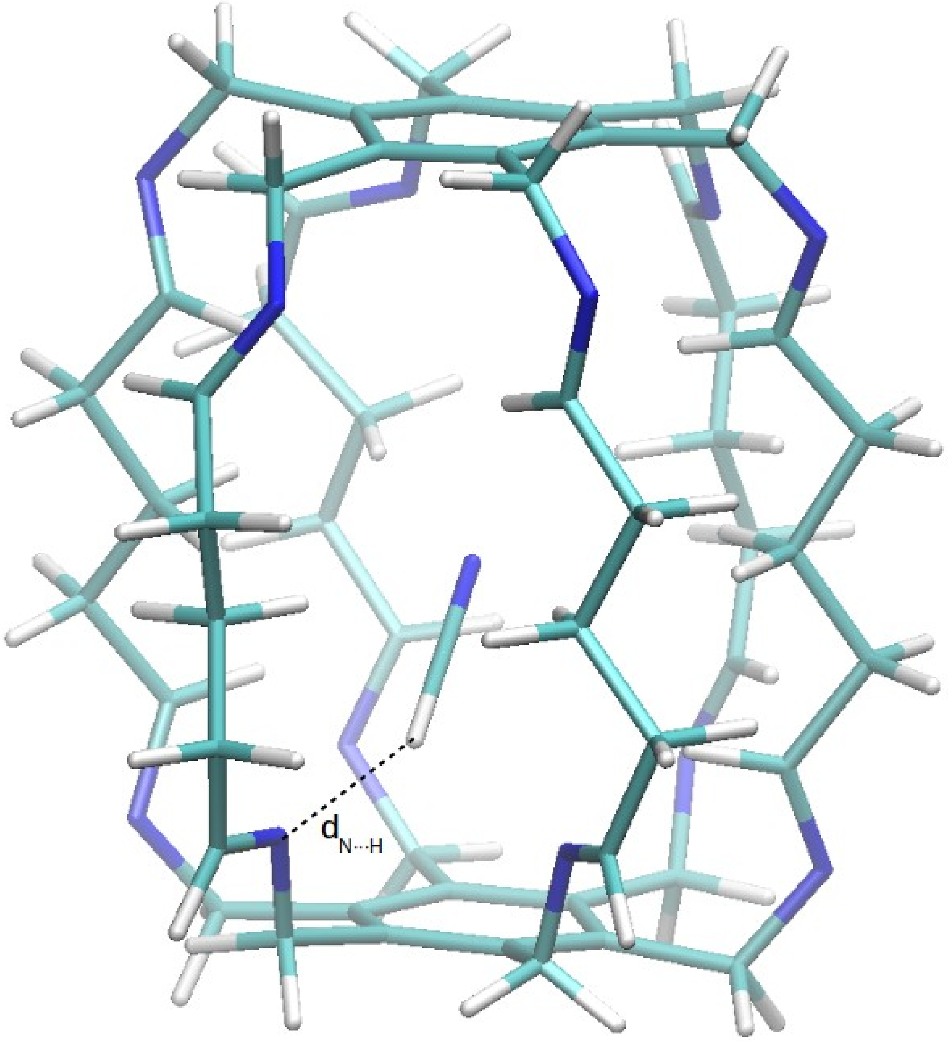

Figure S3: An example of the definition of the $d_{N\cdots H}$ distance shown for the HCN@**3** system.

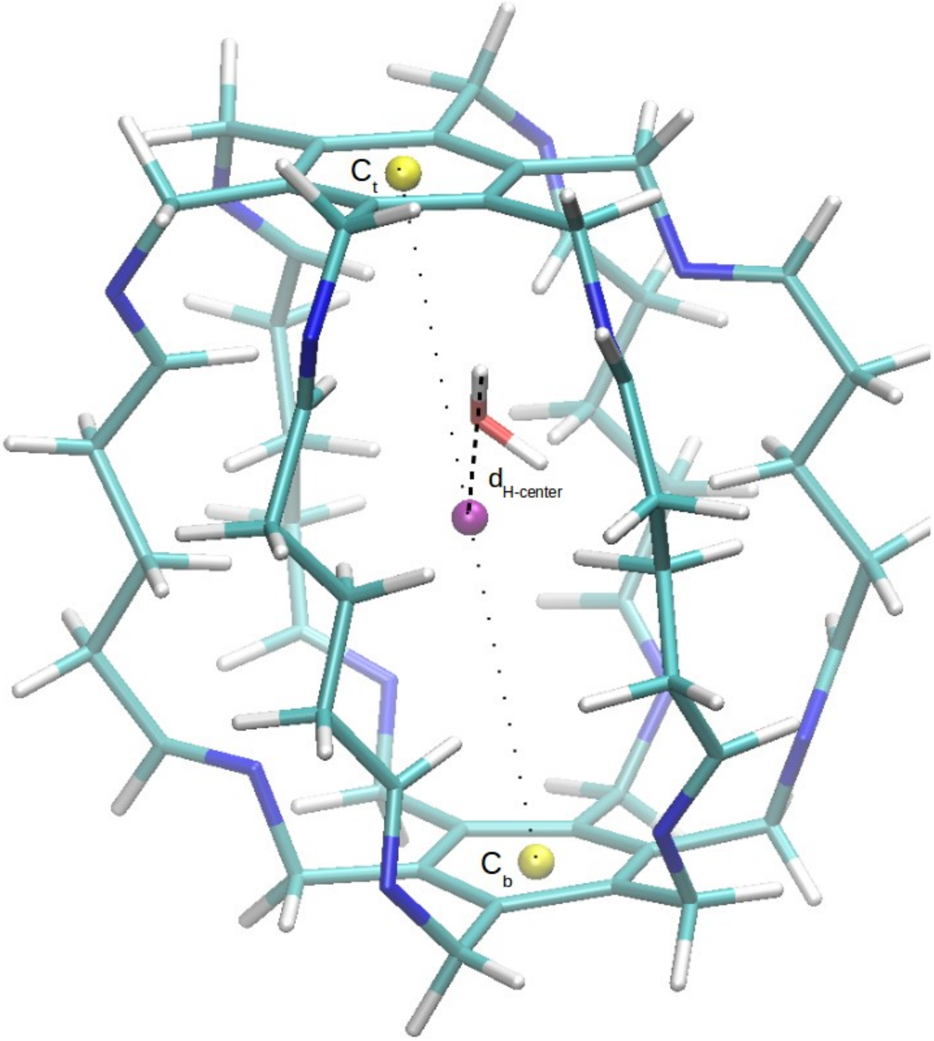

Figure S4: An example of the definition of the $d_{H\text{-center}}$ distance shown for the H_2O@**3** system. The cage center (purple sphere) is the midpoint of the line segment connecting the centers of the bottom and the top benzene rings (yellow spheres).

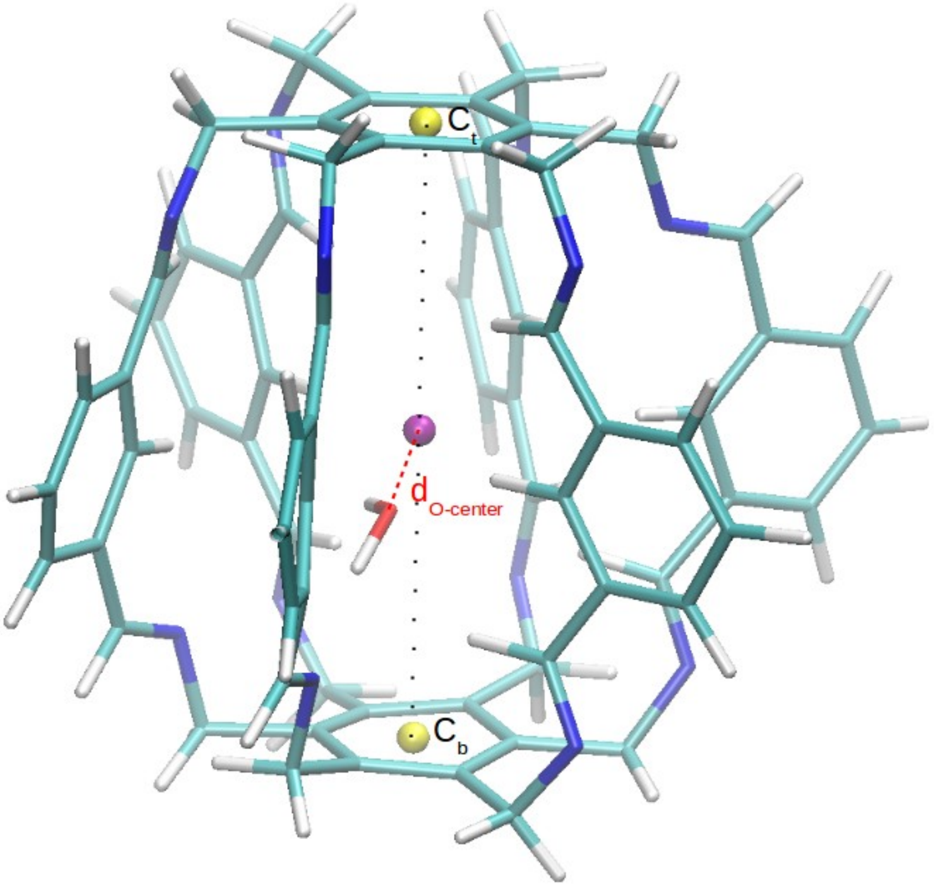

Figure S5: Definition of the $d_{O\text{-center}}$ distance in H_2O@**1**. The cage center (purple sphere) is the midpoint of the line segment connecting the centers of the bottom and the top benzene rings (yellow spheres).

Force field parameter files

Force field parameters for M@SPx; x = 1,2,3; M = H_2O, HF, HCN, NH_3, MeOH.
Parameter files are in the Tinker v7 format.

```
      ##  Force Field for SP-1
      ##  Tinker 7 parameter format



      ##############################
      ##                          ##
      ##  Force Field Definition  ##
      ##                          ##
      ##############################


forcefield               OPLS-AA

vdwtype                  LENNARD-JONES
radiusrule               GEOMETRIC
radiustype               SIGMA
radiussize               DIAMETER
epsilonrule              GEOMETRIC
torsionunit              0.5
vdw-14-scale             2.0
chg-14-scale             2.0
dielectric               1.0


      #############################
      ##                         ##
      ##  Atom Type Definitions  ##
      ##                         ##
      #############################


atom          6    2    HC      "HR Alkane"                 1      1.008     1
atom         10    4    HC      "H-C=C Alkene"              1      1.008     1
atom         11    5    CA      "CH Benzene"                6      12.01     3
atom        311    5    CA      "CH Benzene"                6      12.01     3
atom         12    6    HA      "H Benzene"                 1      1.008     1
atom        103    1    CT      "C"                         6      12.01     4
atom        209   51    CP      "CH"                        6      12.01     3
atom        214   54    NB      "N"                         7      14.01     2

atom        222   56    OW      "O Water (TIP3P)"           8      16.00     2
atom        223   57    HW      "H Water (TIP3P)"           1      1.008     1

atom        401   401   F       "F HF"                      9      19.00     1
atom        402   402   H       "H HF"                      1      1.008     1
```

```
atom        423  423    NT      "N Ammonia"                   7      14.01     3
atom        424  424    H2      "H Ammonia"                   1      1.008     1

atom        501   501   C       "C HCN"                       6      12.01     2
atom        502   502   N       "N HCN"                       7      14.01     1
atom        503   503   H       "H HCN"                       1      1.008     1

atom         38   12    OH      "OH Alcohol"                  8      16.00     2
atom         39   13    HO      "HO Alcohol"                  1      1.008     1
atom         40    2    HC      "CH3 Methanol"                1      1.008     1
atom         41    1    CT      "CH2 & CH3 Alcohol"           6      12.01     4

      ################################
      ##                            ##
      ##  Van der Waals Parameters  ##
      ##                            ##
      ################################


vdw          1             3.5000     0.0660
vdw          2             2.5000     0.0300
vdw          4             2.4200     0.0300
vdw          5             3.5500     0.0700
vdw          6             2.4200     0.0300
vdw         12             3.1200     0.1700
vdw         13             0.0000     0.0000
vdw         51             3.5500     0.0700
vdw         54             3.2500     0.1700
vdw         56        3.150656111     0.152072595
vdw         57             0.0000     0.0000
vdw        401             2.8500     0.0610
vdw        402             0.000      0.0300
vdw        423             3.2500     0.1700
vdw        424             0.0000     0.0000
vdw        501             3.3000     0.0660
vdw        502             3.2000     0.1700
vdw        503             0.0000     0.0000

      ##################################
      ##                              ##
      ##  Bond Stretching Parameters  ##
      ##                              ##
      ##################################

bond         1   54          382.0     1.4480
bond         5   51          317.0     1.5100
bond         4   51          367.0     1.0800
bond         1    1          268.0     1.5290
bond         1    2          340.0     1.0900
bond         1    5          317.0     1.5100
bond         5    5          469.0     1.4000
bond         5    6          367.0     1.0800
bond        51   54          488.0     1.3350
bond        56   57          529.6     0.9572
```

```
bond        401   402         704.0     0.922
bond        423   424         520.0     1.013
bond        501   502        1700.0     1.154
bond        501   503         430.0     1.070

bond          1   12          320.0     1.4100
bond         12   13          553.0     0.9450

      ################################
      ##                            ##
      ##  Angle Bending Parameters  ##
      ##                            ##
      ################################

angle         1    54   51    70.00     118.00
angle         5    5    51    63.00     120.00
angle         54   51   4     63.00     121.00
angle         5    51   54    63.00     120.00
angle         4    51   5     63.00     116.00
angle         54   1    2     63.00     106.50
angle         54   1    5     63.00     119.00
angle         1    1    1     58.35     112.70
angle         1    1    2     37.50     110.70
angle         1    1    5     63.00     114.00
angle         2    1    2     33.00     107.80
angle         2    1    5     35.00     109.50
angle         1    5    5     70.00     120.00
angle         5    5    5     63.00     120.00
angle         5    5    6     35.00     120.00
angle         6   51   54     35.00     120.00
angle         1   55    2     37.50     110.70
angle        57   56   57     34.05     104.52
angle       424  423  424     47.1      108.32
angle       502  501  503     32.0      180.0

angle         2    1   12     35.00     109.50
angle         1   12   13     55.00     108.50

      ###############################
      ##                           ##
      ##  Urey-Bradley Parameters  ##
      ##                           ##
      ###############################


ureybrad     57   56   57     38.25     1.5139


      #####################################
      ##                                 ##
      ##  Improper Torsional Parameters  ##
      ##                                 ##
      #####################################
```

```
imptors        5    5    5    1                    2.200 180.0 2
imptors        5    5    5    6                    2.200 180.0 2



      ############################
      ##                        ##
      ##  Torsional Parameters  ##
      ##                        ##
      ############################


torsion        2    1   54    51        0.000 0.0 1    0.000 180.0 2    0.000 0.0 3
torsion        5    1   54    51        0.000 0.0 1    0.000 180.0 2    0.000 0.0 3
torsion        1    54  51    5      0.880 0.0 1   10.000 180.0 2    0.000 0.0 3
torsion        1    54  51    4      0.000 0.0 1   12.200 180.0 2    0.000 0.0 3
torsion        51   5   5     6      0.250 0.0 1    9.000 180.0 2   -0.550 0.0 3
torsion        51   5   5     5     -0.670 0.0 1   10.000 180.0 2    0.000 0.0 3
torsion        54   51  5     5      1.000 0.0 1   10.000 180.0 2    0.000 0.0 3
torsion        54   1   5     5         0.000 0.0 1    0.000 180.0 2    0.300 0.0 3
torsion        4    51  5     5      0.250 0.0 1    9.000 180.0 2   -0.550 0.0 3
torsion        1    5   5     1     -0.300 0.0 1    9.500 180.0 2    0.000 0.0 3
torsion        1    1    1    1      1.300 0.0 1   -0.050 180.0 2    0.200 0.0 3
torsion        1    1    1    2                                     0.300 0.0 3
torsion        1    1    1    5      1.300 0.0 1   -0.050 180.0 2    0.200 0.0 3
torsion        2    1    1    2                                     0.300 0.0 3
torsion        2    1    1    5                                     0.462 0.0 3
torsion        1    1    5    5
torsion        2    1    5    5
torsion        1    5    5    5                    7.250 180.0 2
torsion        1    5    5    6                    7.250 180.0 2
torsion        5    5    5    5                    7.250 180.0 2
torsion        5    5    5    6                    7.250 180.0 2
torsion        6    5    5    6                    7.250 180.0 2
torsion        6   51   54   52                   10.000 180.0 2
torsion       50   51   54   52                   10.000 180.0 2

torsion        2    1   12   13                                     0.450 0.0 3


      ########################################
      ##                                    ##
      ##  Atomic Partial Charge Parameters  ##
      ##                                    ##
      ########################################

charge        6        0.1
charge       10        0.06
charge       11       -0.09
charge       12        0.1
charge       38       -0.8330
charge       39        0.5680
charge       40        0.0400
```

```
charge       41        0.1450
charge      103       -0.03
charge      209        0.26
charge      214       -0.41
charge      222       -0.8340
charge      223        0.4170
charge      311       -0.05
charge      401       -0.6
charge      402        0.6
charge      423       -1.044
charge      424        0.348
charge      501        0.117
charge      502       -0.348
charge      503        0.231
```

```
      ##  Force Field for SP-2
      ##  Tinker 7 parameter format


      ##############################
      ##                          ##
      ##  Force Field Definition  ##
      ##                          ##
      ##############################


forcefield              OPLS-AA

vdwtype                 LENNARD-JONES
radiusrule              GEOMETRIC
radiustype              SIGMA
radiussize              DIAMETER
epsilonrule             GEOMETRIC
torsionunit             0.5
vdw-14-scale            2.0
chg-14-scale            2.0
dielectric              1.0


      #############################
      ##                         ##
      ##  Atom Type Definitions  ##
      ##                         ##
      #############################

atom          3    1    CT      "R3CH Alkane"                 6      12.01     4
atom          6    2    HC      "HR Alkane"                   1      1.008     1
atom          8    3    CM      "RHC=C Alkene"                6      12.01     3
atom         10    4    HC      "H-C=C Alkene"                1      1.008     1
atom        311    5    CA      "CH Benzene"                  6      12.01     3
atom         12    6    HA      "H Benzene"                   1      1.008     1
atom        801    3    CM      "RHC=C Alkene"                6      12.01     3
atom        811    4    HC      "H-C=C Alkene"                1      1.008     1
atom        103    1    CT      "C"                           6      12.01     4
atom        209   51    CP      "CH"                          6      12.01     3
atom        214   54    NB      "N"                           7      14.01     2

atom        222   56    OW      "O Water (TIP3P)"             8      16.00     2
atom        223   57    HW      "H Water (TIP3P)"             1      1.008     1

atom        401   401   F       "F HF"                        9      19.00     1
atom        402   402   H       "H HF"                        1      1.008     1

atom        423  423    NT      "N Ammonia"                   7      14.01     3
atom        424  424    H2      "H Ammonia"                   1      1.008     1

atom        501   501   C       "C HCN"                       6      12.01     2
atom        502   502   N       "N HCN"                       7      14.01     1
atom        503   503   H       "H HCN"                       1      1.008     1
```

```
atom          38   12    OH      "OH Alcohol"              8      16.00     2
atom          39   13    HO      "HO Alcohol"              1      1.008     1
atom          40    2    HC      "CH3 Methanol"            1      1.008     1
atom          41    1    CT      "CH2 & CH3 Alcohol"       6      12.01     4


      ################################
      ##                            ##
      ##  Van der Waals Parameters  ##
      ##                            ##
      ################################


vdw            1             3.5000     0.0660
vdw            2             2.5000     0.0300
vdw            3             3.5500     0.0760
vdw            4             2.4200     0.0300
vdw            5             3.5500     0.0700
vdw           12             3.1200     0.1700
vdw           13             0.0000     0.0000
vdw           51             3.5500     0.0700
vdw           54             3.2500     0.1700
vdw           56        3.150656111     0.152072595
vdw           57             0.0000     0.0000
vdw          401             2.8500     0.0610
vdw          402             0.000      0.0300
vdw          423             3.2500     0.1700
vdw          424             0.0000     0.0000
vdw          501            3.3000     0.0660
vdw          502             3.2000     0.1700
vdw          503             0.0000     0.0000


      ##################################
      ##                              ##
      ##  Bond Stretching Parameters  ##
      ##                              ##
      ##################################

bond           1   54          382.0     1.4480
bond           5   51          317.0     1.5100
bond           4   51          367.0     1.0800
bond           1    1          268.0     1.5290
bond           1    2          340.0     1.0900
bond           1    5          317.0     1.5100
bond           5    5          469.0     1.4000
bond           5    6          367.0     1.0800
bond          51   54          488.0     1.3350
bond          56   57          529.6     0.9572
bond         401   402         704.0     0.922
bond         423   424         520.0     1.013
bond         501   502        1700.0     1.154
bond         501   503         430.0     1.070
```

```
bond          1   12          320.0     1.4100
bond         12   13          553.0     0.9450


bond          3   51          469.0     1.4000
bond          1   51         317.0     1.5100
bond          1    3          317.0     1.5100
bond          3    4          340.00     1.0800
bond          3    3          549.00     1.340


      ################################
      ##                            ##
      ##  Angle Bending Parameters  ##
      ##                            ##
      ################################

angle         1    54   51    70.00     118.00
angle         5    5    51    63.00     120.00
angle         54   51   4     63.00     121.00
angle         5    51   54    63.00     120.00
angle         4    51   5     63.00     116.00
angle         54   1    2     63.00     106.50
angle         54   1    5     63.00     119.00
angle         1    1    1     58.35     112.70
angle         1    1    2     37.50     110.70
angle         1    1    5     63.00     114.00
angle         2    1    2     33.00     107.80
angle         2    1    5     35.00     109.50
angle         1    5    5     70.00     120.00
angle         5    5    5     63.00     120.00
angle         5    5    6     35.00     120.00
angle         6   51   54     35.00     120.00
angle         1   55    2     37.50     110.70
angle        57   56   57     34.05     104.52
angle       424  423  424     47.1      108.32
angle       502  501  503     32.0      180.0
angle         54   51   3     63.00     120.0
angle         4    51   3     63.00     115.0
angle         54   51   1     63.00     121.0
angle         4    51   1     63.00     115.0
angle         2     1  51     63.00     109.0
angle         3     1  51     63.00     115.0
angle         2     1   3     63.00     110.0
angle         4     3  51     63.00     116.0
angle         3     3  51     63.00     120.0
angle         4     3   3     63.00     120.0
angle         1     3   3     63.00     123.0
angle         1     3   4     63.00     116.0
angle         2    1   12     35.00     109.50
angle         1   12   13     55.00     108.50

      ###############################
```

```
      ##                            ##
      ##  Urey-Bradley Parameters   ##
      ##                            ##
      ################################


ureybrad     57   56   57     38.25     1.5139

      ######################################
      ##                                  ##
      ##  Improper Torsional Parameters   ##
      ##                                  ##
      ######################################


imptors       5    5    5    1                     2.200 180.0 2
imptors       5    5    5    6                     2.200 180.0 2


      ############################
      ##                        ##
      ##  Torsional Parameters  ##
      ##                        ##
      ############################

torsion       2    1   54    51        0.000 0.0 1    0.000 180.0 2    0.000 0.0 3
torsion       5    1   54    51        0.000 0.0 1    0.000 180.0 2    0.000 0.0 3
torsion       1    54  51    5      0.880 0.0 1   10.000 180.0 2    0.000 0.0 3
torsion       1    54  51    4      0.000 0.0 1   12.200 180.0 2    0.000 0.0 3
torsion       51   5   5     6      0.250 0.0 1    9.000 180.0 2   -0.550 0.0 3
torsion       51   5   5     5     -0.670 0.0 1   10.000 180.0 2    0.000 0.0 3
torsion       54   51  5     5      1.000 0.0 1   10.000 180.0 2    0.000 0.0 3
torsion       54   1   5     5         0.000 0.0 1    0.000 180.0 2    0.300 0.0 3
torsion       4    51  5     5      0.250 0.0 1    9.000 180.0 2   -0.550 0.0 3
torsion       1    5   5     1     -0.300 0.0 1    9.500 180.0 2    0.000 0.0 3
torsion       1    1    1    1      1.300 0.0 1   -0.050 180.0 2    0.200 0.0 3
torsion       1    1    1    2                                      0.300 0.0 3
torsion       1    1    1    5      1.300 0.0 1   -0.050 180.0 2    0.200 0.0 3
torsion       2    1    1    2                                      0.300 0.0 3
torsion       2    1    1    5                                      0.462 0.0 3
torsion       1    1    5    5
torsion       2    1    5    5
torsion       1    5    5    5                     7.250 180.0 2
torsion       1    5    5    6                     7.250 180.0 2
torsion       5    5    5    5                     7.250 180.0 2
torsion       5    5    5    6                     7.250 180.0 2
torsion       6    5    5    6                     7.250 180.0 2
torsion       6   51   54   52                    10.000 180.0 2
torsion      50   51   54   52                    10.000 180.0 2

torsion       2    1   12   13                                      0.450 0.0 3

torsion       54   51  3     4      0.755 0.0 1   10.000 180.0 2    0.000 0.0 3
torsion       54   51  3     3      1.000 0.0 1   10.000 180.0 2    0.000 0.0 3
```

```
torsion       54   51  1     2       0.000 0.0 1    0.000 180.0 2    0.000 0.0 3
torsion       54   51  1     3       0.000 0.0 1    0.000 180.0 2    0.000 0.0 3
torsion        4   51  3     4       0.000 0.0 1   11.500 180.0 2    0.000 0.0 3
torsion        1   54  51    3       0.000 0.0 1   10.000 180.0 2    0.000 0.0 3
torsion        1   54  51    1       0.880 0.0 1   10.000 180.0 2    0.000 0.0 3
torsion        4   51  3     3       0.250 0.0 1    9.000 180.0 2   -0.550 0.0 3
torsion        4   51  1     2       0.000 0.0 1    0.000 180.0 2    0.580 0.0 3
torsion        4   51  1     3       0.000 0.0 1    0.000 180.0 2    0.800 0.0 3
torsion       51    1  3     3       0.250 0.0 1   -0.650 180.0 2    0.600 0.0 3
torsion       51    1  3     4       0.000 0.0 1    0.000 180.0 2    0.800 0.0 3
torsion        2    1  3     3       0.000 0.0 1    0.000 180.0 2   -0.090 0.0 3
torsion        2    1  3     4       0.000 0.0 1    0.000 180.0 2    0.580 0.0 3
torsion       51    3  3     1      -0.610 0.0 1    7.000 180.0 2    0.000 0.0 3
torsion       51    3  3     4       0.250 0.0 1    9.000 180.0 2   -0.550 0.0 3
torsion        4    3  3     1       0.000 0.0 1   10.000 180.0 2    0.000 0.0 3
torsion        4    3  3     4       0.000 0.0 1   11.500 180.0 2    0.000 0.0 3


      ########################################
      ##                                    ##
      ##  Atomic Partial Charge Parameters  ##
      ##                                    ##
      ########################################


charge        3        -0.32
charge        6         0.11
charge        8         0.07
charge       10         0.05
charge       38        -0.8330
charge       39         0.5680
charge       40         0.0400
charge       41         0.1450
charge      103        -0.03
charge      209         0.35
charge      214        -0.46
charge      222        -0.8340
charge      223         0.4170
charge      311        -0.04
charge      401        -0.6
charge      402         0.6
charge      423        -1.044
charge      424         0.348
charge      501         0.117
charge      502        -0.348
charge      503         0.231
charge      801        -0.35
charge      811         0.15
```

```
##  Force Field for SP-3
##  Tinker 7 parameter format


##############################
##                          ##
##  Force Field Definition  ##
##                          ##
##############################


forcefield              OPLS-AA

vdwtype                 LENNARD-JONES
radiusrule              GEOMETRIC
radiustype              SIGMA
radiussize              DIAMETER
epsilonrule             GEOMETRIC
torsionunit             0.5
vdw-14-scale            2.0
chg-14-scale            2.0
dielectric              1.0


#############################
##                         ##
##  Atom Type Definitions  ##
##                         ##
#############################


atom          3    1    CT      "R3CH Alkane"                6      12.01     4
atom        303    1    CT      "R3CH Alkane"                6      12.01     4
atom          6    2    HC      "HR Alkane"                  1      1.008     1
atom        603    2    HC      "HR Alkane"                  1      1.008     1
atom         10    4    HC      "H-C=C Alkene"               1      1.008     1
atom        311    5    CA      "CH Benzene"                 6      12.01     3
atom        103    1    CT      "C"                          6      12.01     4
atom        209   51    CP      "CH"                         6      12.01     3
atom        214   54    NB      "N"                          7      14.01     2

atom        222   56    OW      "O Water (TIP3P)"            8      16.00     2
atom        223   57    HW      "H Water (TIP3P)"            1      1.008     1

atom        401   401   F       "F HF"                       9      19.00     1
atom        402   402   H       "H HF"                       1      1.008     1

atom        423  423    NT      "N Ammonia"                  7      14.01     3
atom        424  424    H2      "H Ammonia"                  1      1.008     1

atom        501   501   C       "C HCN"                      6      12.01     2
atom        502   502   N       "N HCN"                      7      14.01     1
atom        503   503   H       "H HCN"                      1      1.008     1
```

```
atom          38   12    OH      "OH Alcohol"                 8       16.00     2
atom          39   13    HO      "HO Alcohol"                 1       1.008     1
atom          40    2    HC      "CH3 Methanol"               1       1.008     1
atom          41    1    CT      "CH2 & CH3 Alcohol"          6       12.01     4


      ################################
      ##                            ##
      ##  Van der Waals Parameters  ##
      ##                            ##
      ################################

vdw           1             3.5000     0.0660
vdw           2             2.5000     0.0300
vdw           4             2.4200     0.0300
vdw           5             3.5500     0.0700
vdw           6             2.4200     0.0300
vdw          12             3.1200     0.1700
vdw          13             0.0000     0.0000
vdw          51             3.5500     0.0700
vdw          54             3.2500     0.1700
vdw          56        3.150656111     0.152072595
vdw          57             0.0000     0.0000
vdw         401             2.8500     0.0610
vdw         402             0.000      0.0300
vdw         423             3.2500     0.1700
vdw         424             0.0000     0.0000
vdw         501             3.3000     0.0660
vdw         502             3.2000     0.1700
vdw         503             0.0000     0.0000


      ##################################
      ##                              ##
      ##  Bond Stretching Parameters  ##
      ##                              ##
      ##################################

bond          1   51          317.0     1.5100
bond          1   54          382.0     1.4480
bond          5   51          317.0     1.5100
bond          4   51          367.0     1.0800
bond          1    1          268.0     1.5290
bond          1    2          340.0     1.0900
bond          1    5          317.0     1.5100
bond          5    5          469.0     1.4000
bond          5    6          367.0     1.0800
bond         51   54          488.0     1.3350
bond         56   57          529.6     0.9572
bond        401   402         704.0     0.922
bond        423   424         520.0     1.013
```

```
bond        501   502        1700.0     1.154
bond        501   503         430.0     1.070

bond          1   12          320.0     1.4100
bond         12   13          553.0     0.9450


      ################################
      ##                            ##
      ##  Angle Bending Parameters  ##
      ##                            ##
      ################################

angle         1    54   51    70.00     118.00
angle         5    5    51    63.00     120.00
angle         54   51   4     63.00     121.00
angle         5    51   54    63.00     120.00
angle         4    51   5     63.00     116.00
angle         54   1    2     63.00     106.50
angle         54   1    5     63.00     119.00
angle         1    1    1     58.35     112.70
angle         1    1    2     37.50     110.70
angle         1    1    5     63.00     114.00
angle         2    1    2     33.00     107.80
angle         2    1    5     35.00     109.50
angle         1    5    5     70.00     120.00
angle         5    5    5     63.00     120.00
angle         5    5    6     35.00     120.00
angle         6   51   54     35.00     120.00
angle         1   55    2     37.50     110.70
angle        57   56   57     34.05     104.52
angle       424  423  424     47.1      108.32
angle       502  501  503     32.0      180.0
angle         2    1   12     35.00     109.50
angle         1   12   13     55.00     108.50

angle         54   51   1     63.00     121.0
angle         4    51   1     63.00     115.0
angle         2     1  51     63.00     109.0
angle        51    1    1     63.00     110.0


      ###############################
      ##                           ##
      ##  Urey-Bradley Parameters  ##
      ##                           ##
      ###############################


ureybrad     57   56   57     38.25     1.5139


      ####################################
      ##                                ##
```

```
      ##  Improper Torsional Parameters  ##
      ##                                 ##
      #####################################

imptors       5    5    5    1                    2.200 180.0 2
imptors       5    5    5    6                    2.200 180.0 2



      ############################
      ##                        ##
      ##  Torsional Parameters  ##
      ##                        ##
      ############################


torsion       2    1   54    51         0.000 0.0 1    0.000 180.0 2    0.000 0.0 3
torsion       5    1   54    51         0.000 0.0 1    0.000 180.0 2    0.000 0.0 3
torsion       1    54  51    5      0.880 0.0 1   10.000 180.0 2    0.000 0.0 3
torsion       1    54  51    4      0.000 0.0 1   12.200 180.0 2    0.000 0.0 3
torsion       51   5   5     6      0.250 0.0 1    9.000 180.0 2   -0.550 0.0 3
torsion       51   5   5     5     -0.670 0.0 1   10.000 180.0 2    0.000 0.0 3
torsion       54   51  5     5      1.000 0.0 1   10.000 180.0 2    0.000 0.0 3
torsion       54   1   5     5         0.000 0.0 1    0.000 180.0 2    0.300 0.0 3
torsion       4    51  5     5      0.250 0.0 1    9.000 180.0 2   -0.550 0.0 3
torsion       1    5   5     1     -0.300 0.0 1    9.500 180.0 2    0.000 0.0 3
torsion       1    1    1    1      1.300 0.0 1   -0.050 180.0 2    0.200 0.0 3
torsion       1    1    1    2                                      0.300 0.0 3
torsion       1    1    1    5      1.300 0.0 1   -0.050 180.0 2    0.200 0.0 3
torsion       2    1    1    2                                      0.300 0.0 3
torsion       2    1    1    5                                      0.462 0.0 3
torsion       1    1    5    5
torsion       2    1    5    5
torsion       1    5    5    5                    7.250 180.0 2
torsion       1    5    5    6                    7.250 180.0 2
torsion       5    5    5    5                    7.250 180.0 2
torsion       5    5    5    6                    7.250 180.0 2
torsion       6    5    5    6                    7.250 180.0 2
torsion       6   51   54   52                   10.000 180.0 2
torsion      50   51   54   52                   10.000 180.0 2

torsion       2    1   12   13                                      0.450 0.0 3
torsion       54   51  1     2      0.000 0.0 1    0.000 180.0 2    0.000 0.0 3
torsion        1   54  51    1      0.880 0.0 1   10.000 180.0 2    0.000 0.0 3
torsion        4   51  1     2      0.000 0.0 1    0.000 180.0 2    0.580 0.0 3

torsion       54   51  1     1      0.000 0.0 1    0.000 180.0 2   -0.250 0.0 3
torsion        4   51  1     1      0.000 0.0 1    0.000 180.0 2    0.010 0.0 3
torsion       51    1  1     1      0.200 0.0 1   -0.200 180.0 2    1.300 0.0 3
torsion       51    1  1     2      0.000 0.0 1    0.000 180.0 2    0.500 0.0 3


      ########################################
      ##                                    ##
```

```
      ##  Atomic Partial Charge Parameters  ##
      ##                                    ##
      ########################################



charge        3       -0.42
charge        6        0.11
charge       10        0.03
charge       38       -0.8330
charge       39        0.5680
charge       40        0.0400
charge       41        0.1450
charge      103       -0.05
charge      209        0.37
charge      214       -0.46
charge      222       -0.8340
charge      223        0.4170
charge      311       -0.03
charge      801       -0.35
charge      811        0.15
charge      303        0.34
charge      603       -0.05
charge      401       -0.6
charge      402        0.6
charge      423       -1.044
charge      424        0.348
charge      501        0.117
charge      502       -0.348
charge      503        0.231
```

Input geometries

Input structures for M@SPx; x = 1,2,3; M = H_2O, HF, HCN, NH_3, MeOH.
The files are in the Tinker .xyz format, extending the classical format with additional columns (type of the atom, list of the atoms bonded to).

H_2O@SP1

```
       147  molden generated tinker .xyz
   1  N    -0.423379    3.901419    1.500279    214   24   51
   2  N    -3.730650    1.403105   -2.985049    214    9   54
   3  N     4.463640    0.390798   -2.640686    214   11   33
   4  N     5.177225    0.665697    0.716512    214   39   41
   5  N     1.789069   -1.769865   -3.368789    214   18   35
   6  C     3.542353    2.416790    0.633710    311   20   22    39
   7  C    -4.586560    1.794565   -0.177243    311    8   46   113
   8  C    -3.910124    2.750166   -0.948733    311    7   50    54
   9  C    -2.558464    1.154034   -3.403092    209    2   10    53
  10  H    -1.762688    1.911183   -3.439385     10    9
  11  C     5.170891   -0.723905   -2.023125    103    3   12    13   118
  12  H     5.982043   -0.270039   -1.449022      6   11
  13  H     5.664106   -1.277758   -2.831708      6   11
  14  C    -3.175440   -1.166520   -4.088758     11   15   48    53
  15  H    -4.216441   -0.867599   -4.027188     12   14
  16  C    -1.478668   -2.829933   -4.453785     11   17   32    48
  17  H    -1.199550   -3.854740   -4.686268     12   16
  18  C     0.905382   -2.368083   -4.055655    209    5   19    32
  19  H     1.119135   -3.325404   -4.557524     10   18
  20  C     4.415978    3.309488   -0.000969     11    6   21    44
  21  H     5.402896    2.953635   -0.277166     12   20
  22  C     2.256998    2.835350    0.973206     11    6   23    29
  23  H     1.557426    2.150844    1.447320     12   22
  24  C    -1.745456    4.484353    1.680363    103    1   25    26    38
  25  H    -1.799481    5.508865    1.285252      6   24
  26  H    -1.926358    4.571377    2.753840      6   24
  27  C    -0.842807   -0.580370   -3.852662     11   28   32    53
  28  H    -0.066711    0.147789   -3.629880     12   27
  29  C     1.839752    4.140318    0.679777    311   22   30    51
  30  C     2.719913    5.011421    0.036496     11   29   31    44
  31  H     2.387587    6.014770   -0.219390     12   30
  32  C    -0.484776   -1.898446   -4.151025    311   16   18    27
  33  C     3.309704    0.691555   -2.206119    209    3   34    61
  34  H     2.811365    0.119838   -1.415484     10   33
  35  C     3.099809   -2.391882   -3.234017    103    5   36    37   119
  36  H     3.817549   -1.728316   -3.717868      6   35
  37  H     3.150832   -3.355716   -3.765485      6   35
  38  C    -2.805406    3.591052    1.049588    311   24   47    50
  39  C     3.960250    1.012933    0.846322    209    4    6    40
  40  H     3.152288    0.304308    1.065298     10   39
  41  C     5.536183   -0.742849    0.871439    103    4   42    43    79
  42  H     5.646622   -0.934833    1.938979      6   41
  43  H     6.530555   -0.870244    0.428758      6   41
  44  C     4.005848    4.598567   -0.300378     11   20   30    45
```

45	H	4.678105	5.279188	-0.813444	12	44			
46	C	-4.383955	1.748804	1.214072	311	7	47	83	
47	C	-3.452365	2.607689	1.819377	311	38	46	107	
48	C	-2.821823	-2.465404	-4.419118	11	14	16	49	
49	H	-3.590699	-3.200861	-4.632440	12	48			
50	C	-3.083552	3.700677	-0.323739	311	8	38	70	
51	C	0.474356	4.618889	0.961325	209	1	29	52	
52	H	0.268168	5.651970	0.634470	10	51			
53	C	-2.185279	-0.214331	-3.812387	311	9	14	27	
54	C	-4.028435	2.734880	-2.475112	103	2	8	55	56
55	H	-5.051313	2.987745	-2.780133	6	54			
56	H	-3.361482	3.478258	-2.920442	6	54			
57	N	-1.309646	4.621229	-1.939366	214	68	70		
58	C	0.591534	3.256911	-2.487970	311	59	66	68	
59	C	1.316518	2.125736	-2.118044	11	58	60	61	
60	H	0.930210	1.470455	-1.338571	12	59			
61	C	2.549306	1.847198	-2.705185	311	33	59	62	
62	C	3.042987	2.695850	-3.698397	11	61	63	64	
63	H	4.007249	2.470573	-4.142728	12	62			
64	C	2.309504	3.811168	-4.089528	11	62	65	66	
65	H	2.696536	4.467675	-4.863313	12	64			
66	C	1.091777	4.100545	-3.483042	11	58	64	67	
67	H	0.519413	4.980659	-3.758640	12	66			
68	C	-0.659533	3.542694	-1.769679	209	57	58	69	
69	H	-0.975700	2.768788	-1.057262	10	68			
70	C	-2.515968	4.844925	-1.155232	103	50	57	71	72
71	H	-3.283825	5.200009	-1.851389	6	70			
72	H	-2.312217	5.713668	-0.522955	6	70			
73	N	0.398915	-3.819925	-1.343184	214	96	123		
74	N	3.716066	-1.358875	3.040970	214	81	126		
75	N	-4.443105	-0.379998	2.643855	214	83	105		
76	N	-5.229692	-0.606275	-0.671522	214	111	113		
77	N	-1.830078	1.794571	3.457510	214	90	107		
78	C	-3.612324	-2.376885	-0.596172	311	92	94	111	
79	C	4.580872	-1.744936	0.234282	311	41	80	118	
80	C	3.913132	-2.707446	1.006357	311	79	122	126	
81	C	2.540200	-1.108707	3.449895	209	74	82	125	
82	H	1.744225	-1.865991	3.480656	10	81			
83	C	-5.157388	0.750887	2.069077	103	46	75	84	85
84	H	-5.977204	0.309482	1.497531	6	83			
85	H	-5.641050	1.280829	2.898857	6	83			
86	C	3.157149	1.200130	4.162118	11	87	120	125	
87	H	4.196630	0.892168	4.124388	12	86			
88	C	1.465755	2.875724	4.491000	11	89	104	120	
89	H	1.190055	3.902884	4.716822	12	88			
90	C	-0.916632	2.435973	4.056084	209	77	91	104	
91	H	-1.092328	3.440162	4.476821	10	90			
92	C	-4.498949	-3.289637	-0.011587	11	78	93	116	
93	H	-5.490117	-2.942210	0.260298	12	92			
94	C	-2.322909	-2.784589	-0.928506	11	78	95	101	
95	H	-1.622667	-2.069637	-1.352924	12	94			
96	C	1.710745	-4.409219	-1.598826	103	73	97	98	110
97	H	1.761741	-5.444721	-1.237083	6	96			
98	H	1.840526	-4.465921	-2.681906	6	96			

99	C	0.823529	0.636127	3.866748	11	100	104	125	
100	H	0.041351	-0.074388	3.610576	12	99			
101	C	-1.912883	-4.101913	-0.682211	311	94	102	123	
102	C	-2.809921	-4.998732	-0.098653	11	101	103	116	
103	H	-2.489238	-6.015932	0.113175	12	102			
104	C	0.469801	1.954847	4.166261	311	88	90	99	
105	C	-3.260836	-0.630163	2.257123	209	75	106	133	
106	H	-2.719474	-0.001255	1.541631	10	105			
107	C	-3.121724	2.445552	3.301398	103	47	77	108	109
108	H	-3.862758	1.807273	3.784415	6	107			
109	H	-3.162410	3.419806	3.816174	6	107			
110	C	2.803930	-3.544733	-0.987507	311	96	119	122	
111	C	-4.015911	-0.966300	-0.783895	209	76	78	112	
112	H	-3.197599	-0.263491	-0.983051	10	111			
113	C	-5.562952	0.810695	-0.811699	103	7	76	114	115
114	H	-5.679994	1.009080	-1.877554	6	113			
115	H	-6.551674	0.951754	-0.360954	6	113			
116	C	-4.098140	-4.592808	0.238029	11	92	102	117	
117	H	-4.781592	-5.292407	0.708752	12	116			
118	C	4.383900	-1.704916	-1.157476	311	11	79	119	
119	C	3.447863	-2.562613	-1.758267	311	35	110	118	
120	C	2.806686	2.500659	4.486912	11	86	88	121	
121	H	3.576335	3.229567	4.719731	12	120			
122	C	3.098026	-3.665311	0.380783	311	80	110	142	
123	C	-0.551163	-4.579226	-0.972127	209	73	101	124	
124	H	-0.387957	-5.657267	-0.818299	10	123			
125	C	2.164325	0.258599	3.857865	311	81	86	99	
126	C	4.020534	-2.689167	2.533903	103	74	80	127	128
127	H	5.041463	-2.940883	2.846678	6	126			
128	H	3.351501	-3.434748	2.973345	6	126			
129	N	1.298771	-4.627802	1.935845	214	140	142		
130	C	-0.583181	-3.245420	2.501341	311	131	138	140	
131	C	-1.276288	-2.084118	2.162552	11	130	132	133	
132	H	-0.854495	-1.399775	1.426221	12	131			
133	C	-2.524325	-1.816242	2.720813	311	105	131	134	
134	C	-3.066452	-2.707955	3.648841	11	133	135	136	
135	H	-4.042597	-2.487939	4.068950	12	134			
136	C	-2.366422	-3.854912	4.007550	11	134	137	138	
137	H	-2.791515	-4.543037	4.732358	12	136			
138	C	-1.131914	-4.131880	3.431674	11	130	136	139	
139	H	-0.581153	-5.032847	3.682648	12	138			
140	C	0.691352	-3.516897	1.822891	209	129	130	141	
141	H	1.067783	-2.699303	1.193335	10	140			
142	C	2.537836	-4.821923	1.197999	103	122	129	143	144
143	H	3.290742	-5.155047	1.921013	6	142			
144	H	2.383426	-5.696692	0.559154	6	142			
145	O	0.576769	-0.952392	-0.822655	222	146	147		
146	H	1.000502	-0.850870	-1.692996	223	145			
147	H	0.435301	-1.918430	-0.831879	223	145			

HF@SP1

```
        146  molden generated tinker .xyz
   1  N    -0.457485   -3.772227   -1.167500    214   24   51
   2  N    -3.832597   -1.166927    3.035328    214  9   54
   3  N     4.381953   -0.366137    2.718417    214   11   33
   4  N     5.279222   -0.655165   -0.615624    214   39   41
   5  N     1.791727    1.875087    3.435917    214   18   35
   6  C     3.638489   -2.403374   -0.546258    311   20   22   39
   7  C    -4.638697   -1.656129    0.220216    311  8   46  113
   8  C    -4.013970   -2.596452    1.053536    311  7   50   54
   9  C    -2.650256   -0.925702    3.430759    209  2   10   53
  10  H    -1.868548   -1.696836    3.482093     10  9
  11  C     5.128888    0.738415    2.133111    103  3   12   13  118
  12  H     5.949595    0.268210    1.586170      6   11
  13  H     5.608797    1.274050    2.961338      6   11
  14  C    -3.222355    1.420543    4.048846     11   15   48   53
  15  H    -4.268158    1.137277    3.997093     12   14
  16  C    -1.496249    3.061964    4.366990     11   17   32   48
  17  H    -1.198995    4.088110    4.568782     12   16
  18  C     0.882372    2.550705    4.002296    209  5   19   32
  19  H     1.074137    3.559451    4.404613     10   18
  20  C     4.551081   -3.376851   -0.122919     11  6   21   44
  21  H     5.572373   -3.070843    0.078232     12   20
  22  C     2.316121   -2.756908   -0.794730     11  6   23   29
  23  H     1.601780   -1.997186   -1.097670     12   22
  24  C    -1.756926   -4.411370   -1.406350    103  1   25   26   38
  25  H    -1.776218   -5.415568   -0.968473      6   24
  26  H    -1.865353   -4.551894   -2.483409      6   24
  27  C    -0.897878    0.789050    3.824115     11   28   32   53
  28  H    -0.128918    0.051198    3.607535     12   27
  29  C     1.900269   -4.083654   -0.632314    311   22   30   51
  30  C     2.823275   -5.044090   -0.210382     11   29   31   44
  31  H     2.500690   -6.073083   -0.071113     12   30
  32  C    -0.517398    2.106766    4.093428    311   16   18   27
  33  C     3.202057   -0.609733    2.319825    209  3   34   61
  34  H     2.679832    0.008882    1.581159     10   33
  35  C     3.099937    2.494648    3.290395    103  5   36   37  119
  36  H     3.818179    1.852221    3.801363      6   35
  37  H     3.152107    3.479195    3.784170      6   35
  38  C    -2.871358   -3.535571   -0.870123    311   24   47   50
  39  C     4.057396   -0.994533   -0.683975    209  4    6   40
  40  H     3.244013   -0.272946   -0.823822     10   39
  41  C     5.618707    0.760064   -0.737396    103  4   42   43   79
  42  H     5.777680    0.954160   -1.799106      6   41
  43  H     6.591757    0.898819   -0.253182      6   41
  44  C     4.144251   -4.690949    0.047014     11   20   30   45
  45  H     4.850573   -5.441569    0.387147     12   44
  46  C    -4.410999   -1.686015   -1.167693    311  7   47   83
  47  C    -3.481437   -2.588738   -1.707300    311   38   46  107
  48  C    -2.846031    2.721317    4.341556     11   14   16   49
  49  H    -3.601647    3.476324    4.533534     12   48
  50  C    -3.200296   -3.594052    0.493738    311  8   38   70
```

51	C	0.525309	-4.529541	-0.876269	209	1	29	52	
52	H	0.368661	-5.613229	-0.780931	10	51			
53	C	-2.247454	0.445252	3.797123	311	9	14	27	
54	C	-4.159607	-2.508876	2.574769	103	2	8	55	56
55	H	-5.193642	-2.722189	2.872182	6	54			
56	H	-3.519989	-3.250579	3.061921	6	54			
57	N	-1.442420	-4.532636	2.112665	214	68	70		
58	C	0.478334	-3.178087	2.621981	311	59	66	68	
59	C	1.201125	-2.049001	2.236272	11	58	60	61	
60	H	0.805748	-1.410060	1.447983	12	59			
61	C	2.438851	-1.768529	2.811931	311	33	59	62	
62	C	2.943875	-2.619301	3.797937	11	61	63	64	
63	H	3.911765	-2.391291	4.232641	12	62			
64	C	2.217121	-3.735685	4.198216	11	62	65	66	
65	H	2.614122	-4.390813	4.968128	12	64			
66	C	0.989990	-4.022248	3.610828	11	58	64	67	
67	H	0.416107	-4.897098	3.899797	12	66			
68	C	-0.794345	-3.452211	1.938951	209	57	58	69	
69	H	-1.126413	-2.665878	1.250530	10	68			
70	C	-2.680701	-4.725292	1.371839	103	50	57	71	72
71	H	-3.449585	-5.000412	2.101930	6	70			
72	H	-2.551021	-5.635381	0.777580	6	70			
73	N	0.458112	3.951296	1.401788	214	96	123		
74	N	3.766653	1.246070	-2.924689	214	81	126		
75	N	-4.384776	0.357862	-2.713409	214	83	105		
76	N	-5.177156	0.789394	0.578116	214	111	113		
77	N	-1.789795	-1.902399	-3.333471	214	90	107		
78	C	-3.497109	2.492724	0.446028	311	92	94	111	
79	C	4.624720	1.750338	-0.143740	311	41	80	118	
80	C	3.958183	2.681134	-0.952337	311	79	122	126	
81	C	2.592843	0.982106	-3.331591	209	74	82	125	
82	H	1.800042	1.739469	-3.401846	10	81			
83	C	-5.141222	-0.718035	-2.090705	103	46	75	84	85
84	H	-5.958581	-0.224521	-1.559799	6	83			
85	H	-5.623926	-1.283046	-2.898262	6	83			
86	C	3.205739	-1.351072	-3.970751	11	87	120	125	
87	H	4.246218	-1.047315	-3.924788	12	86			
88	C	1.510765	-3.022796	-4.305107	11	89	104	120	
89	H	1.233257	-4.049563	-4.532385	12	88			
90	C	-0.880173	-2.557349	-3.924479	209	77	91	104	
91	H	-1.065973	-3.559477	-4.348632	10	90			
92	C	-4.328049	3.369093	-0.264124	11	78	93	116	
93	H	-5.307342	3.015289	-0.568374	12	92			
94	C	-2.220235	2.906773	0.820961	11	78	95	101	
95	H	-1.552775	2.234953	1.355659	12	94			
96	C	1.785166	4.517326	1.597823	103	73	97	98	110
97	H	1.865666	5.531185	1.180312	6	96			
98	H	1.946848	4.625856	2.672248	6	96			
99	C	0.871832	-0.770956	-3.717294	11	100	104	125	
100	H	0.089137	-0.053741	-3.484273	12	99			
101	C	-1.770253	4.191964	0.491220	311	94	102	123	
102	C	-2.608495	5.047159	-0.225225	11	101	103	116	
103	H	-2.249104	6.033271	-0.509863	12	102			
104	C	0.514849	-2.091568	-4.008950	311	88	90	99	

105	C	-3.199278	0.589736	-2.322286	209	75	106	133	
106	H	-2.680244	-0.016535	-1.571747	10	105			
107	C	-3.101686	-2.512592	-3.185476	103	47	77	108	109
108	H	-3.810109	-1.880119	-3.722331	6	107			
109	H	-3.152958	-3.510933	-3.652190	6	107			
110	C	2.840542	3.591384	1.007437	311	96	119	122	
111	C	-3.952970	1.109186	0.707365	209	76	78	112	
112	H	-3.167218	0.389385	0.968033	10	111			
113	C	-5.579119	-0.600106	0.787715	103	7	76	114	115
114	H	-5.718379	-0.738557	1.860291	6	113			
115	H	-6.566876	-0.720087	0.328626	6	113			
116	C	-3.884879	4.637974	-0.600238	11	92	102	117	
117	H	-4.522964	5.304374	-1.172175	12	116			
118	C	4.392232	1.734234	1.243673	311	11	79	119	
119	C	3.460110	2.617569	1.811649	311	35	110	118	
120	C	2.853533	-2.654950	-4.281316	11	86	88	121	
121	H	3.623067	-3.390620	-4.492248	12	120			
122	C	3.135242	3.657902	-0.364993	311	80	110	142	
123	C	-0.409180	4.661471	0.806089	209	73	101	124	
124	H	-0.174824	5.677843	0.447655	10	123			
125	C	2.214447	-0.398678	-3.696971	311	81	86	99	
126	C	4.071323	2.599904	-2.477197	103	74	80	127	128
127	H	5.093108	2.835116	-2.799452	6	126			
128	H	3.404773	3.326051	-2.950871	6	126			
129	N	1.393290	4.547335	-2.030715	214	140	142		
130	C	-0.492652	3.165962	-2.587943	311	131	138	140	
131	C	-1.203818	2.020240	-2.236302	11	130	132	133	
132	H	-0.817081	1.350300	-1.469771	12	131			
133	C	-2.431012	1.736813	-2.830262	311	105	131	134	
134	C	-2.927763	2.588973	-3.818695	11	133	135	136	
135	H	-3.890420	2.360309	-4.264571	12	134			
136	C	-2.202370	3.712670	-4.200208	11	134	137	138	
137	H	-2.590746	4.370218	-4.972491	12	136			
138	C	-0.993891	4.011363	-3.581183	11	130	136	139	
139	H	-0.429825	4.900748	-3.843572	12	138			
140	C	0.743509	3.469760	-1.854198	209	129	130	141	
141	H	1.052099	2.709619	-1.123591	10	140			
142	C	2.591062	4.782223	-1.238067	103	122	129	143	144
143	H	3.372159	5.104578	-1.935886	6	142			
144	H	2.391263	5.672891	-0.635822	6	142			
145	F	-0.563759	-1.192104	-0.547558	401	146			
146	H	-0.541590	-2.092179	-0.917528	402	145			

HCN@SP1

```
       147  molden generated tinker .xyz
  1  N     0.415591   -3.854050    1.594557    214   24   51
  2  N     3.386943   -1.547153   -3.212905    214  9   54
  3  N    -4.646949   -0.336496   -2.387176    214   11   33
  4  N    -5.132027   -0.502462    0.953945    214   39   41
  5  N    -2.034477    1.785156   -3.417104    214   18   35
  6  C    -3.554181   -2.306723    0.834498    311   20   22   39
  7  C     4.429470   -1.857876   -0.457582    311  8   46  113
  8  C     3.670338   -2.825217   -1.137247    311  7   50   54
  9  C     2.198677   -1.250346   -3.546917    209  2   10   53
 10  H     1.364638   -1.963685   -3.486174     10  9
 11  C    -5.257464    0.832319   -1.766530    103  3   12   13  118
 12  H    -6.045155    0.431140   -1.124668      6   11
 13  H    -5.785938    1.375164   -2.559714      6   11
 14  C     2.869920    1.000522   -4.389864     11   15   48   53
 15  H     3.896288    0.649574   -4.407434     12   14
 16  C     1.230036    2.735885   -4.670036     11   17   32   48
 17  H     0.982137    3.766078   -4.913579     12   16
 18  C    -1.136322    2.401597   -4.063001    209  5   19   32
 19  H    -1.301304    3.412439   -4.471060     10   18
 20  C    -4.414288   -3.148031    0.118298     11  6   21   44
 21  H    -5.373952   -2.753615   -0.198756     12   20
 22  C    -2.301043   -2.775541    1.221294     11  6   23   29
 23  H    -1.639832   -2.132237    1.794617     12   22
 24  C     1.740286   -4.450936    1.729912    103  1   25   26   38
 25  H     1.748376   -5.494017    1.387558      6   24
 26  H     1.986708   -4.481641    2.792623      6   24
 27  C     0.540471    0.548973   -3.923807     11   28   32   53
 28  H    -0.250774   -0.115166   -3.586063     12   27
 29  C    -1.887367   -4.067831    0.874153    311   22   30   51
 30  C    -2.757251   -4.890602    0.155021     11   29   31   44
 31  H    -2.430103   -5.882960   -0.145022     12   30
 32  C     0.221768    1.869391   -4.249647    311   16   18   27
 33  C    -3.442921   -0.627989   -2.114598    209  3   34   61
 34  H    -2.811140   -0.008487   -1.467366     10   33
 35  C    -3.289875    2.475298   -3.172465    103  5   36   37  119
 36  H    -4.085034    1.855433   -3.588949      6   35
 37  H    -3.342642    3.446171   -3.692085      6   35
 38  C     2.758563   -3.602878    0.977570    311   24   47   50
 39  C    -3.931514   -0.898522    1.083717    209  4    6   40
 40  H    -3.103781   -0.224116    1.333124     10   39
 41  C    -5.419204    0.921731    1.139435    103  4   42   43   79
 42  H    -5.428366    1.119605    2.211548      6   41
 43  H    -6.440115    1.091638    0.779831      6   41
 44  C    -4.018246   -4.433975   -0.215466     11   20   30   45
 45  H    -4.677267   -5.073028   -0.794154     12   44
 46  C     4.364508   -1.773301    0.944775    311  7   47   83
 47  C     3.488724   -2.610200    1.655388    311   38   46  107
 48  C     2.552138    2.303206   -4.738948     11   14   16   49
 49  H     3.333954    2.989972   -5.047182     12   48
 50  C     2.894191   -3.749377   -0.415265    311  8   38   70
 51  C    -0.533028   -4.573404    1.146313    209  1   29   52
```

52	H	-0.363962	-5.627171	0.878049	10	51			
53	C	1.860920	0.114805	-3.987173	311	9	14	27	
54	C	3.658898	-2.868231	-2.668154	103	2	8	55	56
55	H	4.639385	-3.189046	-3.041966	6	54			
56	H	2.921571	-3.594618	-3.022937	6	54			
57	N	0.970847	-4.771654	-1.821837	214	68	70		
58	C	-0.904014	-3.367914	-2.419990	311	59	66	68	
59	C	-1.531375	-2.160932	-2.114910	11	58	60	61	
60	H	-1.039432	-1.450913	-1.457224	12	59			
61	C	-2.799251	-1.860559	-2.603365	311	33	59	62	
62	C	-3.430851	-2.770110	-3.454204	11	61	63	64	
63	H	-4.421464	-2.533316	-3.829483	12	62			
64	C	-2.796547	-3.962742	-3.791838	11	62	65	66	
65	H	-3.290136	-4.664328	-4.458075	12	64			
66	C	-1.544305	-4.273569	-3.269534	11	58	64	67	
67	H	-1.053492	-5.212647	-3.505622	12	66			
68	C	0.395569	-3.639387	-1.785244	209	57	58	69	
69	H	0.832979	-2.785548	-1.252886	10	68			
70	C	2.253626	-4.929771	-1.146396	103	50	57	71	72
71	H	2.956019	-5.303316	-1.900494	6	70			
72	H	2.134119	-5.773063	-0.460686	6	70			
73	N	-0.436743	3.919381	-1.599511	214	96	123		
74	N	-3.441448	1.548326	3.191673	214	81	126		
75	N	4.640267	0.356760	2.374464	214	83	105		
76	N	5.077022	0.516175	-1.043539	214	111	113		
77	N	2.013196	-1.733143	3.370273	214	90	107		
78	C	3.513646	2.331618	-0.903874	311	92	94	111	
79	C	-4.475913	1.878277	0.417718	311	41	80	118	
80	C	-3.731715	2.836583	1.121952	311	79	122	126	
81	C	-2.244484	1.290232	3.525992	209	74	82	125	
82	H	-1.431301	2.026240	3.461664	10	81			
83	C	5.251798	-0.788375	1.708358	103	46	75	84	85
84	H	6.008237	-0.363759	1.044484	6	83			
85	H	5.813194	-1.340775	2.472027	6	83			
86	C	-2.867176	-0.962576	4.399108	11	87	120	125	
87	H	-3.899345	-0.628837	4.407969	12	86			
88	C	-1.196314	-2.658228	4.736248	11	89	104	120	
89	H	-0.928525	-3.673898	5.016850	12	88			
90	C	1.164108	-2.298186	4.122755	209	77	91	104	
91	H	1.379353	-3.252400	4.631493	10	90			
92	C	4.445715	3.203253	-0.325681	11	78	93	116	
93	H	5.433195	2.819779	-0.091456	12	92			
94	C	2.226403	2.786208	-1.186024	11	78	95	101	
95	H	1.480271	2.114372	-1.602412	12	94			
96	C	-1.760818	4.515189	-1.689274	103	73	97	98	110
97	H	-1.776470	5.544942	-1.304498	6	96			
98	H	-2.022807	4.591782	-2.746276	6	96			
99	C	-0.545788	-0.477304	3.928659	11	100	104	125	
100	H	0.235140	0.199692	3.588682	12	99			
101	C	1.866333	4.107325	-0.890272	311	94	102	123	
102	C	2.804056	4.958611	-0.304028	11	101	103	116	
103	H	2.515970	5.974684	-0.044506	12	102			
104	C	-0.204068	-1.784051	4.291983	311	88	90	99	
105	C	3.465734	0.698810	2.036607	209	75	106	133	

106	H	2.879922	0.136946	1.302616	10	105			
107	C	3.289444	-2.394476	3.152244	103	47	77	108	109
108	H	4.063639	-1.734995	3.546083	6	107			
109	H	3.368643	-3.344304	3.706068	6	107			
110	C	-2.774463	3.637997	-0.965466	311	96	119	122	
111	C	3.870070	0.912424	-1.120580	209	76	78	112	
112	H	3.026109	0.236527	-1.299000	10	111			
113	C	5.362149	-0.909396	-1.203459	103	7	76	114	115
114	H	5.366068	-1.127889	-2.271265	6	113			
115	H	6.384973	-1.074098	-0.845733	6	113			
116	C	4.091653	4.509038	-0.025361	11	92	102	117	
117	H	4.809488	5.173981	0.444704	12	116			
118	C	-4.384116	1.812003	-0.985148	311	11	79	119	
119	C	-3.494557	2.655153	-1.670207	311	35	110	118	
120	C	-2.526570	-2.249478	4.785214	11	86	88	121	
121	H	-3.296453	-2.940913	5.112278	12	120			
122	C	-2.939257	3.766559	0.424471	311	80	110	142	
123	C	0.499571	4.616695	-1.101195	209	73	101	124	
124	H	0.328510	5.649710	-0.753938	10	123			
125	C	-1.875362	-0.065846	3.978442	311	81	86	99	
126	C	-3.753968	2.865156	2.652325	103	74	80	127	128
127	H	-4.753231	3.141279	3.010820	6	126			
128	H	-3.052676	3.612482	3.032494	6	126			
129	N	-1.073866	4.721854	1.933913	214	140	142		
130	C	0.863096	3.367629	2.413996	311	131	138	140	
131	C	1.548200	2.221151	2.021700	11	130	132	133	
132	H	1.124905	1.593396	1.244541	12	131			
133	C	2.786546	1.892463	2.567711	311	105	131	134	
134	C	3.338635	2.725094	3.543358	11	133	135	136	
135	H	4.307166	2.471133	3.962627	12	134			
136	C	2.651203	3.864616	3.954202	11	134	137	138	
137	H	3.084013	4.507815	4.714906	12	136			
138	C	1.420532	4.194114	3.392874	11	130	136	139	
139	H	0.885061	5.088555	3.695711	12	138			
140	C	-0.413729	3.653752	1.739283	209	129	130	141	
141	H	-0.748093	2.876145	1.038334	10	140			
142	C	-2.313247	4.927286	1.195096	103	122	129	143	144
143	H	-3.045958	5.311003	1.913636	6	142			
144	H	-2.131861	5.774062	0.527671	6	142			
145	C	0.553460	-1.022194	0.583776	501	146	147		
146	N	0.546200	-0.130614	-0.152664	502	145			
147	H	0.549511	-1.910838	1.218879	503	145			

NH_3@SP1

```
       148  molden generated tinker .xyz
   1  N     0.319747   -3.767333    1.651851     214   24   51
   2  N     3.479087   -1.686304   -3.098088     214  9   54
   3  N    -4.606045   -0.369162   -2.441457     214   11   33
   4  N    -5.191559   -0.400419    0.901127     214   39   41
   5  N    -1.939649    1.653792   -3.467004     214   18   35
   6  C    -3.629298   -2.222870    0.851719     311   20   22   39
   7  C     4.452631   -1.924633   -0.312007     311  8   46  113
   8  C     3.706518   -2.901937   -0.990032     311  7   50   54
   9  C     2.296974   -1.412140   -3.470643     209  2   10   53
  10  H     1.470304   -2.135819   -3.420622      10  9
  11  C    -5.225561    0.816130   -1.860793     103  3   12   13  118
  12  H    -6.022231    0.433291   -1.218911       6   11
  13  H    -5.740154    1.337623   -2.677067       6   11
  14  C     2.979725    0.827706   -4.326440      11   15   48   53
  15  H     4.006659    0.478896   -4.307583      12   14
  16  C     1.345568    2.553516   -4.683051      11   17   32   48
  17  H     1.102739    3.578959   -4.950025      12   16
  18  C    -1.033712    2.231360   -4.137763     209  5   19   32
  19  H    -1.192728    3.218392   -4.602055      10   18
  20  C    -4.511520   -3.085505    0.188449      11  6   21   44
  21  H    -5.477262   -2.697952   -0.118210      12   20
  22  C    -2.367329   -2.680858    1.222842      11  6   23   29
  23  H    -1.670466   -2.023343    1.736817      12   22
  24  C     1.624847   -4.388287    1.841442     103  1   25   26   38
  25  H     1.624059   -5.440796    1.525860       6   24
  26  H     1.837362   -4.400123    2.911757       6   24
  27  C     0.637911    0.376475   -3.922816      11   28   32   53
  28  H    -0.163924   -0.287684   -3.607955      12   27
  29  C    -1.976534   -3.992126    0.927740     311   22   30   51
  30  C    -2.866619   -4.837292    0.263482      11   29   31   44
  31  H    -2.555380   -5.847059    0.007152      12   30
  32  C     0.327136    1.691982   -4.277507     311   16   18   27
  33  C    -3.420465   -0.675307   -2.113190     209  3   34   61
  34  H    -2.812182   -0.055596   -1.443092      10   33
  35  C    -3.196265    2.362123   -3.281551     103  5   36   37  119
  36  H    -3.985969    1.730291   -3.690062       6   35
  37  H    -3.228195    3.309553   -3.844061       6   35
  38  C     2.687085   -3.585376    1.106332     311   24   47   50
  39  C    -3.997634   -0.806838    1.057258     209  4    6   40
  40  H    -3.168417   -0.131961    1.301734      10   39
  41  C    -5.460010    1.032766    1.034048     103  4   42   43   79
  42  H    -5.487868    1.268701    2.098068       6   41
  43  H    -6.469629    1.207246    0.645531       6   41
  44  C    -4.130998   -4.385449   -0.104003      11   20   30   45
  45  H    -4.807750   -5.043420   -0.639734      12   44
  46  C     4.334139   -1.793568    1.083943     311  7   47   83
  47  C     3.413322   -2.589283    1.783540     311   38   46  107
  48  C     2.669943    2.123384   -4.705733      11   14   16   49
  49  H     3.459164    2.807087   -5.001350      12   48
  50  C     2.880819   -3.784939   -0.271739     311  8   38   70
```

51	C	-0.625243	-4.497808	1.219401	209	1	29	52	
52	H	-0.466158	-5.563662	0.989480	10	51			
53	C	1.961258	-0.055210	-3.940458	311	9	14	27	
54	C	3.754601	-2.991400	-2.517262	103	2	8	55	56
55	H	4.753404	-3.301242	-2.848239	6	54			
56	H	3.042941	-3.737896	-2.881551	6	54			
57	N	1.020090	-4.802506	-1.753655	214	68	70		
58	C	-0.873222	-3.420388	-2.341789	311	59	66	68	
59	C	-1.522244	-2.227707	-2.030690	11	58	60	61	
60	H	-1.066891	-1.552504	-1.312568	12	59			
61	C	-2.761726	-1.909311	-2.575522	311	33	59	62	
62	C	-3.349423	-2.798093	-3.478348	11	61	63	64	
63	H	-4.319913	-2.551052	-3.896806	12	62			
64	C	-2.696749	-3.980481	-3.816437	11	62	65	66	
65	H	-3.157253	-4.665078	-4.522637	12	64			
66	C	-1.467077	-4.301308	-3.248477	11	58	64	67	
67	H	-0.960164	-5.230434	-3.489405	12	66			
68	C	0.396262	-3.701205	-1.653671	209	57	58	69	
69	H	0.759708	-2.886297	-1.015214	10	68			
70	C	2.254269	-4.975910	-0.996169	103	50	57	71	72
71	H	2.990061	-5.395042	-1.691220	6	70			
72	H	2.065232	-5.790498	-0.291337	6	70			
73	N	-0.349715	3.818215	-1.693912	214	96	123		
74	N	-3.499642	1.689191	3.088547	214	81	126		
75	N	4.603327	0.387720	2.437486	214	83	105		
76	N	5.147027	0.421777	-0.945686	214	111	113		
77	N	1.908274	-1.657221	3.451707	214	90	107		
78	C	3.585310	2.244985	-0.872502	311	92	94	111	
79	C	-4.483562	1.943651	0.298275	311	41	80	118	
80	C	-3.734935	2.914570	0.980561	311	79	122	126	
81	C	-2.313417	1.418638	3.452055	209	74	82	125	
82	H	-1.487250	2.140312	3.388014	10	81			
83	C	5.209823	-0.798954	1.846936	103	46	75	84	85
84	H	6.005481	-0.421754	1.200482	6	83			
85	H	5.725937	-1.332245	2.655334	6	83			
86	C	-2.987459	-0.802189	4.365552	11	87	120	125	
87	H	-4.013206	-0.449085	4.358510	12	86			
88	C	-1.351643	-2.520765	4.750974	11	89	104	120	
89	H	-1.105529	-3.535525	5.054074	12	88			
90	C	1.023758	-2.212664	4.167816	209	77	91	104	
91	H	1.202872	-3.174929	4.676345	10	90			
92	C	4.506979	3.131561	-0.301121	11	78	93	116	
93	H	5.487756	2.753049	-0.033395	12	92			
94	C	2.305328	2.692517	-1.192014	11	78	95	101	
95	H	1.568212	2.013188	-1.612999	12	94			
96	C	-1.661601	4.430431	-1.847844	103	73	97	98	110
97	H	-1.668325	5.476885	-1.511685	6	96			
98	H	-1.892993	4.461803	-2.914344	6	96			
99	C	-0.651932	-0.369744	3.907157	11	100	104	125	
100	H	0.145132	0.280526	3.553615	12	99			
101	C	1.940744	4.021708	-0.945560	311	94	102	123	
102	C	2.867986	4.888405	-0.365515	11	101	103	116	
103	H	2.576101	5.911672	-0.141478	12	102			
104	C	-0.338597	-1.674078	4.302004	311	88	90	99	

105	C	3.416981	0.698961	2.106727	209	75	106	133	
106	H	2.799172	0.090097	1.439238	10	105			
107	C	3.176364	-2.341960	3.270479	103	47	77	108	109
108	H	3.952957	-1.689912	3.671938	6	107			
109	H	3.228658	-3.285756	3.838669	6	107			
110	C	-2.711727	3.607133	-1.114061	311	96	119	122	
111	C	3.944000	0.821445	-1.040501	209	76	78	112	
112	H	3.101288	0.139772	-1.204143	10	111			
113	C	5.422936	-1.010601	-1.052034	103	7	76	114	115
114	H	5.462508	-1.259237	-2.112735	6	113			
115	H	6.431535	-1.172754	-0.655628	6	113			
116	C	4.148355	4.445537	-0.046233	11	92	102	117	
117	H	4.856321	5.122562	0.421275	12	116			
118	C	-4.358041	1.814781	-1.097287	311	11	79	119	
119	C	-3.434257	2.609695	-1.794438	311	35	110	118	
120	C	-2.675022	-2.087570	4.778054	11	86	88	121	
121	H	-3.460747	-2.758776	5.109721	12	120			
122	C	-2.908756	3.799235	0.264420	311	80	110	142	
123	C	0.582800	4.529510	-1.207319	209	73	101	124	
124	H	0.414298	5.578655	-0.912719	10	123			
125	C	-1.973978	0.066857	3.937409	311	81	86	99	
126	C	-3.779116	2.995668	2.508564	103	74	80	127	128
127	H	-4.777275	3.302741	2.844119	6	126			
128	H	-3.067561	3.741116	2.873776	6	126			
129	N	-1.040042	4.792147	1.744996	214	140	142		
130	C	0.860256	3.422379	2.320812	311	131	138	140	
131	C	1.508585	2.225518	2.024845	11	130	132	133	
132	H	1.060734	1.503063	1.343359	12	131			
133	C	2.761627	1.936938	2.562376	311	105	131	134	
134	C	3.357936	2.849592	3.434416	11	133	135	136	
135	H	4.338249	2.622117	3.841532	12	134			
136	C	2.700327	4.034492	3.755926	11	134	137	138	
137	H	3.165724	4.740753	4.437457	12	136			
138	C	1.460454	4.330366	3.197733	11	130	136	139	
139	H	0.950496	5.262248	3.421793	12	138			
140	C	-0.416497	3.689629	1.640854	209	129	130	141	
141	H	-0.781479	2.867959	1.008476	10	140			
142	C	-2.275930	4.979701	0.997838	103	122	129	143	144
143	H	-3.007682	5.394689	1.699933	6	142			
144	H	-2.088002	5.800765	0.300447	6	142			
145	N	0.606270	-0.532059	0.440801	423	146	147	148	
146	H	-0.381834	-0.350760	0.284622	424	145			
147	H	0.677011	-1.346515	1.052383	424	145			
148	H	1.009421	-0.787703	-0.456438	424	145			

MeOH@SP1

```
   150  molden generated tinker .xyz
  1  N     0.132606   3.711557  -1.785951     214   24   51
  2  N     3.527089   1.928754   2.954970     214  9   54
  3  N    -4.579172   0.319991   2.514496     214   11   33
  4  N    -5.232788   0.173442  -0.875637     214   39   41
  5  N    -1.748673  -1.536624   3.525499     214   18   35
  6  C    -3.741667   2.049879  -0.896808     311   20   22   39
  7  C     4.423947   2.081259   0.091153     311  8   46  113
  8  C     3.663911   3.046166   0.772563     311  7   50   54
  9  C     2.364529   1.683627   3.403326     209  2   10   53
 10  H     1.534196   2.401267   3.342033      10  9
 11  C    -5.162307  -0.907053   1.985179     103  3   12   13  118
 12  H    -5.988769  -0.581485   1.349508       6   11
 13  H    -5.635087  -1.425035   2.829330       6   11
 14  C     3.098992  -0.458530   4.449931      11   15   48   53
 15  H     4.113753  -0.075458   4.427500      12   14
 16  C     1.517631  -2.209663   4.910309      11   17   32   48
 17  H     1.303164  -3.217452   5.257301      12   16
 18  C    -0.862852  -2.006785   4.299729     209  5   19   32
 19  H    -1.024597  -2.933044   4.876132      10   18
 20  C    -4.694335   2.929752  -0.367051      11  6   21   44
 21  H    -5.658631   2.528268  -0.074119      12   20
 22  C    -2.483635   2.530230  -1.251598      11  6   23   29
 23  H    -1.727607   1.857138  -1.648930      12   22
 24  C     1.411926   4.367099  -2.016163     103  1   25   26   38
 25  H     1.387424   5.426090  -1.722898       6   24
 26  H     1.598330   4.365009  -3.091817       6   24
 27  C     0.752635  -0.119945   3.975333      11   28   32   53
 28  H    -0.065704   0.491760   3.601023      12   27
 29  C    -2.170612   3.883974  -1.078903     311   22   30   51
 30  C    -3.128565   4.745341  -0.542569      11   29   31   44
 31  H    -2.877992   5.791259  -0.381718      12   30
 32  C     0.479918  -1.416877   4.419977     311   16   18   27
 33  C    -3.422689   0.666245   2.122601     209  3   34   61
 34  H    -2.815346   0.061634   1.438919      10   33
 35  C    -3.000114  -2.267695   3.401315     103  5   36   37  119
 36  H    -3.785163  -1.622116   3.796754       6   35
 37  H    -3.007497  -3.184508   4.014276       6   35
 38  C     2.523189   3.616649  -1.297388     311   24   47   50
 39  C    -4.041091   0.604408  -0.983362     209  4    6   40
 40  H    -3.169142  -0.052719  -1.087788      10   39
 41  C    -5.462631  -1.270170  -0.891273     103  4   42   43   79
 42  H    -5.532725  -1.576226  -1.935392       6   41
 43  H    -6.450756  -1.442562  -0.449880       6   41
 44  C    -4.388188   4.269656  -0.189127      11   20   30   45
 45  H    -5.121696   4.943436   0.242520      12   44
 46  C     4.247417   1.898102  -1.293355     311  7   47   83
 47  C     3.258114   2.626041  -1.973686     311   38   46  107
 48  C     2.825542  -1.731993   4.924169      11   14   16   49
 49  H     3.630541  -2.360390   5.291456      12   48
 50  C     2.770482   3.869556   0.062585     311  8   38   70
```

51	C	-0.835838	4.426329	-1.381468	209	1	29	52	
52	H	-0.716982	5.507076	-1.197176	10	51			
53	C	2.061129	0.357450	3.979000	311	9	14	27	
54	C	3.777575	3.201237	2.291420	103	2	8	55	56
55	H	4.789575	3.523440	2.564935	6	54			
56	H	3.083303	3.964822	2.651833	6	54			
57	N	0.931577	4.859206	1.584269	214	68	70		
58	C	-0.930264	3.457972	2.218379	311	59	66	68	
59	C	-1.588069	2.265981	1.922927	11	58	60	61	
60	H	-1.191099	1.594638	1.169419	12	59			
61	C	-2.786573	1.925892	2.541872	311	33	59	62	
62	C	-3.333911	2.795496	3.486795	11	61	63	64	
63	H	-4.272752	2.529538	3.962248	12	62			
64	C	-2.676499	3.982844	3.799174	11	62	65	66	
65	H	-3.103219	4.655549	4.537440	12	64			
66	C	-1.481555	4.320277	3.169973	11	58	64	67	
67	H	-0.968042	5.249020	3.398505	12	66			
68	C	0.308982	3.756088	1.483522	209	57	58	69	
69	H	0.654278	2.953784	0.820819	10	68			
70	C	2.127003	5.059056	0.773934	103	50	57	71	72
71	H	2.872542	5.532264	1.421661	6	70			
72	H	1.875709	5.840183	0.050977	6	70			
73	N	-0.111113	-3.629197	1.734068	214	96	123		
74	N	-3.562609	-1.945372	-2.968951	214	81	126		
75	N	4.562447	-0.316167	-2.578684	214	83	105		
76	N	5.280409	-0.201250	0.654733	214	111	113		
77	N	1.721273	1.580201	-3.554202	214	90	107		
78	C	3.828308	-2.104458	0.858545	311	92	94	111	
79	C	-4.430884	-2.104674	-0.143057	311	41	80	118	
80	C	-3.657423	-3.073210	-0.800835	311	79	122	126	
81	C	-2.396956	-1.621438	-3.352482	209	74	82	125	
82	H	-1.532044	-2.293598	-3.267472	10	81			
83	C	5.135984	0.923362	-2.066182	103	46	75	84	85
84	H	5.982380	0.612193	-1.450734	6	83			
85	H	5.577261	1.447190	-2.923344	6	83			
86	C	-3.180507	0.555430	-4.291628	11	87	120	125	
87	H	-4.194312	0.172781	-4.240940	12	86			
88	C	-1.612838	2.319609	-4.751393	11	89	104	120	
89	H	-1.410121	3.338876	-5.071091	12	88			
90	C	0.787381	2.096793	-4.236849	209	77	91	104	
91	H	0.912105	3.061810	-4.755933	10	90			
92	C	4.651003	-2.935693	0.088018	11	78	93	116	
93	H	5.585869	-2.532272	-0.286492	12	92			
94	C	2.601617	-2.582087	1.315166	11	78	95	101	
95	H	1.955022	-1.950056	1.918923	12	94			
96	C	-1.386785	-4.283691	2.014014	103	73	97	98	110
97	H	-1.351354	-5.352377	1.762312	6	96			
98	H	-1.551282	-4.239823	3.091817	6	96			
99	C	-0.819476	0.205171	-3.905070	11	100	104	125	
100	H	0.007648	-0.416484	-3.571393	12	99			
101	C	2.176659	-3.870989	0.981232	311	94	102	123	
102	C	3.012475	-4.691332	0.221925	11	101	103	116	
103	H	2.675690	-5.686531	-0.057454	12	102			
104	C	-0.559099	1.512386	-4.323801	311	88	90	99	

105	C	3.391424	-0.648587	-2.221282	209	75	106	133	
106	H	2.773139	-0.017129	-1.576243	10	105			
107	C	2.967070	2.321313	-3.440563	103	47	77	108	109
108	H	3.753803	1.692373	-3.858648	6	107			
109	H	2.956676	3.249407	-4.035922	6	107			
110	C	-2.516911	-3.580612	1.281746	311	96	119	122	
111	C	4.190833	-0.692984	1.088006	209	76	78	112	
112	H	3.438953	-0.086649	1.603093	10	111			
113	C	5.471842	1.243760	0.819879	103	7	76	114	115
114	H	5.486696	1.484612	1.882854	6	113			
115	H	6.461758	1.488539	0.418065	6	113			
116	C	4.247833	-4.225430	-0.219615	11	92	102	117	
117	H	4.878341	-4.860949	-0.832906	12	116			
118	C	-4.267538	-1.894104	1.237694	311	11	79	119	
119	C	-3.273564	-2.595030	1.937457	311	35	110	118	
120	C	-2.921535	1.843373	-4.732417	11	86	88	121	
121	H	-3.738528	2.485579	-5.045375	12	120			
122	C	-2.757410	-3.869737	-0.072579	311	80	110	142	
123	C	0.839788	-4.372395	1.333293	209	73	101	124	
124	H	0.690538	-5.452119	1.177602	10	123			
125	C	-2.126325	-0.273329	-3.883816	311	81	86	99	
126	C	-3.750937	-3.235519	-2.319951	103	74	80	127	128
127	H	-4.740945	-3.613249	-2.603307	6	126			
128	H	-3.010907	-3.959548	-2.673321	6	126			
129	N	-0.880580	-4.887955	-1.533069	214	140	142		
130	C	0.921011	-3.471187	-2.277324	311	131	138	140	
131	C	1.520739	-2.232353	-2.064292	11	130	132	133	
132	H	1.007476	-1.515223	-1.432633	12	131			
133	C	2.765978	-1.925695	-2.607571	311	105	131	134	
134	C	3.394758	-2.866864	-3.425965	11	133	135	136	
135	H	4.367161	-2.627603	-3.844906	12	134			
136	C	2.782461	-4.091927	-3.677721	11	134	137	138	
137	H	3.274988	-4.816593	-4.319738	12	136			
138	C	1.556822	-4.406259	-3.097218	11	130	136	139	
139	H	1.086934	-5.371298	-3.259853	12	138			
140	C	-0.333644	-3.740826	-1.555594	209	129	130	141	
141	H	-0.739590	-2.880497	-1.006472	10	140			
142	C	-2.096943	-5.067645	-0.751790	103	122	129	143	144
143	H	-2.826585	-5.546812	-1.414336	6	142			
144	H	-1.870874	-5.839948	-0.010482	6	142			
145	O	-0.478071	-0.681178	0.329658	38	146	147		
146	H	-0.400228	-1.353969	1.028582	39	145			
147	C	0.658165	0.158691	0.355690	41	145	148	149	150
148	H	0.544893	0.893673	-0.448995	40	147			
149	H	0.763517	0.689455	1.310688	40	147			
150	H	1.587793	-0.393960	0.184620	40	147			

H_2O@SP2

```
        129  molden generated tinker .xyz
   1  N     2.414653   -2.919900   -1.286573    214   15   32
   2  N     3.170251    2.896544   -1.885116    214    8   34
   3  N    -4.011525    0.167799   -3.471544    214   10   18
   4  N    -4.016560   -2.969566   -1.774912    214   24   26
   5  N    -2.985262    2.945266   -1.613026    214   13   20
   6  C     4.688800    1.250265   -0.034407    311    7   29    68
   7  C     4.421016    0.899440   -1.369111    311    6   31    34
   8  C     2.009235    2.933411   -2.404076    209    2    9  113
   9  H     1.729351    2.354831   -3.296452     10    8
  10  C    -5.062848   -0.088714   -2.490819    103    3   11    12   71
  11  H    -5.432142   -1.090404   -2.715938      6   10
  12  H    -5.892357    0.584603   -2.741272      6   10
  13  C    -2.675086    4.138654   -1.334409    209    5   14    97
  14  H    -3.413001    4.846552   -0.919304     10   13
  15  C     3.860588   -2.856254   -1.083202    103    1   16    17   23
  16  H     4.407201   -3.225290   -1.961470      6   15
  17  H     4.110538   -3.535388   -0.266880      6   15
  18  C    -2.827766    0.358877   -3.069187    209    3   19    94
  19  H    -2.547651    0.353386   -2.009937     10   18
  20  C    -4.365322    2.532644   -1.373845    103    5   21    22   72
  21  H    -4.792180    2.296024   -2.349496      6   20
  22  H    -4.974906    3.352854   -0.960527      6   20
  23  C     4.261784   -1.435055   -0.728840    311   15   30    31
  24  C    -2.820589   -2.559356   -1.718755    209    4   25    91
  25  H    -2.473112   -1.826062   -0.978647     10   24
  26  C    -4.981203   -2.460202   -0.803281    103    4   27    28   48
  27  H    -5.069761   -3.229110   -0.035600      6   26
  28  H    -5.953991   -2.432726   -1.306840      6   26
  29  C     4.749251    0.251790    0.955211    311    6   30    52
  30  C     4.478539   -1.084455    0.612967    311   23   29    62
  31  C     4.292820   -0.451811   -1.732332    311    7   23    40
  32  C     1.977493   -3.698374   -2.194844    209    1   33  109
  33  H     2.674932   -4.306035   -2.790517     10   32
  34  C     4.181791    1.996320   -2.409842    103    2    7    35   36
  35  H     5.099446    2.576082   -2.572613      6   34
  36  H     3.890407    1.565097   -3.374069      6   34
  37  N     2.981461   -0.754936   -3.949412    214   38   40
  38  C     1.929611   -0.380513   -3.338960    209   37   39  111
  39  H     1.910776   -0.149154   -2.267282     10   38
  40  C     4.231762   -0.841970   -3.208073    103   31   37    41   42
  41  H     4.956458   -0.229874   -3.759008      6   40
  42  H     4.596154   -1.865326   -3.337146      6   40
  43  N    -2.383143    2.882729    1.698254    214   57   74
  44  N    -3.228608   -3.051897    1.445920    214   50   76
  45  N     4.013419    0.502804    3.410657    214   52   60
  46  N     3.940802    3.286638    1.290358    214   66   68
  47  N     2.939108   -2.523894    1.835243    214   55   62
  48  C    -4.700730   -1.094284   -0.186722    311   26   49    71
  49  C    -4.424558   -0.961004    1.182290    311   48   73    76
  50  C    -2.236182   -3.407107    2.145570    209   44   51    88
```

51	H	-2.127534	-3.119908	3.203205	10	50			
52	C	5.067989	0.615337	2.406227	103	29	45	53	54
53	H	5.431274	1.642453	2.475608	6	52			
54	H	5.900195	-0.009918	2.754580	6	52			
55	C	2.666041	-3.749004	2.029195	209	47	56	107	
56	H	3.463041	-4.506807	2.117795	10	55			
57	C	-3.825656	2.795642	1.501376	103	43	58	59	65
58	H	-4.380880	2.988377	2.431528	6	57			
59	H	-4.110771	3.594621	0.817395	6	57			
60	C	2.827174	0.282906	3.014271	209	45	61	105	
61	H	2.577272	0.155658	1.957153	10	60			
62	C	4.339425	-2.142810	1.700847	103	30	47	63	64
63	H	4.654709	-1.735698	2.662778	6	62			
64	H	4.993301	-3.007477	1.499381	6	62			
65	C	-4.195975	1.442300	0.913293	311	57	72	73	
66	C	2.729933	2.903969	1.207800	209	46	67	103	
67	H	2.393461	2.150064	0.483789	10	66			
68	C	4.919557	2.701383	0.376300	103	6	46	69	70
69	H	4.964122	3.353591	-0.496468	6	68			
70	H	5.897877	2.778339	0.863745	6	68			
71	C	-4.732553	0.046685	-1.007186	311	10	48	72	
72	C	-4.434563	1.309514	-0.464951	311	20	65	71	
73	C	-4.244401	0.311702	1.748254	311	49	65	82	
74	C	-1.970743	3.117050	2.872006	209	43	75	100	
75	H	-2.667201	3.255322	3.715605	10	74			
76	C	-4.264862	-2.215682	2.036340	103	44	49	77	78
77	H	-5.196222	-2.796011	2.036228	6	76			
78	H	-4.043442	-1.967521	3.080472	6	76			
79	N	-2.966564	0.163933	4.005609	214	80	82		
80	C	-1.940653	-0.258336	3.395849	209	79	81	85	
81	H	-1.905971	-0.386455	2.303400	10	80			
82	C	-4.186973	0.457907	3.266244	103	73	79	83	84
83	H	-4.962128	-0.180738	3.707546	6	82			
84	H	-4.479894	1.468396	3.558559	6	82			
85	C	-0.702704	-0.655952	4.154670	3	80	86	87	115
86	H	-0.716694	-0.204282	5.151263	6	85			
87	H	-0.745214	-1.747680	4.301767	6	85			
88	C	-1.150031	-4.268791	1.554385	3	50	89	90	117
89	H	-1.137913	-5.234619	2.077178	6	88			
90	H	-1.425156	-4.455424	0.508943	6	88			
91	C	-1.799155	-3.012583	-2.730460	3	24	92	93	119
92	H	-2.065976	-4.000593	-3.117542	6	91			
93	H	-1.872153	-2.311624	-3.578149	6	91			
94	C	-1.709030	0.677478	-4.024799	3	18	95	96	121
95	H	-1.860355	0.170663	-4.982969	6	94			
96	H	-1.771674	1.758142	-4.223763	6	94			
97	C	-1.299805	4.715035	-1.506894	3	13	98	99	123
98	H	-0.940381	5.007337	-0.510866	6	97			
99	H	-1.406672	5.653544	-2.073478	6	97			
100	C	-0.517760	3.181986	3.245546	3	74	101	102	125
101	H	-0.377945	2.441900	4.050753	6	100			
102	H	-0.308835	4.154907	3.710517	6	100			
103	C	1.705103	3.398610	2.125168	801	66	104	125	
104	H	2.028078	4.146290	2.847293	811	103			

105	C	1.690845	0.108207	3.909646	801	60	106	115
106	H	1.824581	0.314468	4.969240	811	105		
107	C	1.293353	-4.270792	2.103463	801	55	108	117
108	H	1.189626	-5.270209	2.525101	811	107		
109	C	0.562116	-3.840263	-2.549895	801	32	110	119
110	H	0.316797	-4.693792	-3.179552	811	109		
111	C	0.659661	-0.203926	-4.038692	801	38	112	121
112	H	0.612120	-0.515506	-5.080170	811	111		
113	C	0.978754	3.750387	-1.764244	801	8	114	123
114	H	1.306957	4.284633	-0.874332	811	113		
115	C	0.546443	-0.347258	3.394739	8	85	105	116
116	H	0.515836	-0.547945	2.322004	10	115		
117	C	0.220024	-3.648375	1.604288	8	88	107	118
118	H	0.339696	-2.667879	1.145837	10	117		
119	C	-0.387037	-2.952152	-2.234718	8	91	109	120
120	H	-0.119803	-2.076554	-1.644817	10	119		
121	C	-0.375054	0.371890	-3.418939	8	94	111	122
122	H	-0.253274	0.660549	-2.372430	10	121		
123	C	-0.287804	3.837175	-2.178619	8	97	113	124
124	H	-0.613387	3.290640	-3.061378	10	123		
125	C	0.472772	2.879641	2.161395	8	100	103	126
126	H	0.177918	2.124920	1.432921	10	125		
127	O	0.994497	-0.646123	-0.109372	222	128	129	
128	H	1.705454	-0.028288	0.105141	223	127		
129	H	1.481760	-1.466721	-0.344472	223	127		

HF@SP2

```
        128  molden generated tinker .xyz
   1  N     2.383004   -2.840536   -1.344379   214   15   32
   2  N     3.254520    2.939324   -1.795733   214    8   34
   3  N    -3.998672    0.329082   -3.468952   214   10   18
   4  N    -4.072670   -2.862184   -1.882962   214   24   26
   5  N    -2.932725    3.018590   -1.512728   214   13   20
   6  C     4.743170    1.219082    0.015204   311    7   29    68
   7  C     4.479613    0.911979   -1.330173   311    6   31    34
   8  C     2.090243    2.991672   -2.305106   209    2    9  113
   9  H     1.801600    2.434055   -3.208221    10    8
  10  C    -5.067581    0.062011   -2.509872   103    3   11    12   71
  11  H    -5.456285   -0.922787   -2.773622     6   10
  12  H    -5.878791    0.761946   -2.746594     6   10
  13  C    -2.596133    4.190707   -1.179102   209    5   14    97
  14  H    -3.320949    4.895342   -0.736284    10   13
  15  C     3.835564   -2.829636   -1.143015   103    1   16    17   23
  16  H     4.352518   -3.187064   -2.041481     6   15
  17  H     4.069249   -3.543755   -0.352591     6   15
  18  C    -2.817954    0.490835   -3.045414   209    3   19    94
  19  H    -2.552414    0.450538   -1.983371    10   18
  20  C    -4.324241    2.629230   -1.298215   103    5   21    22   72
  21  H    -4.748784    2.436927   -2.284548     6   20
  22  H    -4.918185    3.448179   -0.860616     6   20
  23  C     4.276041   -1.433440   -0.752640   311   15   30    31
  24  C    -2.875324   -2.462488   -1.800676   209    4   25    91
  25  H    -2.533720   -1.752253   -1.036341    10   24
  26  C    -5.039639   -2.369019   -0.905026   103    4   27    28   48
  27  H    -5.148536   -3.162003   -0.164565     6   26
  28  H    -6.006654   -2.308721   -1.416504     6   26
  29  C     4.777713    0.192934    0.976541   311    6   30    52
  30  C     4.486929   -1.128065    0.598348   311   23   29    62
  31  C     4.330244   -0.425692   -1.729080   311    7   23    40
  32  C     1.910115   -3.651831   -2.208698   209    1   33  109
  33  H     2.593062   -4.316866   -2.753927    10   32
  34  C     4.256634    2.040951   -2.340878   103    2    7    35   36
  35  H     5.181530    2.613859   -2.486079     6   34
  36  H     3.962044    1.640054   -3.317498     6   34
  37  N     2.978569   -0.668105   -3.919819   214   38   40
  38  C     1.944143   -0.287330   -3.283123   209   37   39  111
  39  H     1.947567   -0.067828   -2.207860    10   38
  40  C     4.245756   -0.776424   -3.210785   103   31   37    41   42
  41  H     4.958458   -0.152030   -3.763120     6   40
  42  H     4.606123   -1.796410   -3.376136     6   40
  43  N    -2.359299    2.845813    1.771684   214   57   74
  44  N    -3.270151   -3.056243    1.313588   214   50   76
  45  N     3.964482    0.380504    3.401438   214   52   60
  46  N     3.977245    3.228609    1.370867   214   66   68
  47  N     2.903473   -2.595818    1.738230   214   55   62
  48  C    -4.740823   -1.030159   -0.240167   311   26   49    71
  49  C    -4.461689   -0.951700    1.132197   311   48   73    76
  50  C    -2.281438   -3.445799    2.000825   209   44   51    88
```

51	H	-2.174596	-3.202471	3.069288	10	50			
52	C	5.057035	0.512812	2.442747	103	29	45	53	54
53	H	5.427426	1.533631	2.555005	6	52			
54	H	5.870345	-0.130901	2.803095	6	52			
55	C	2.613323	-3.812481	1.957697	209	47	56	107	
56	H	3.398117	-4.575144	2.099111	10	55			
57	C	-3.804815	2.781482	1.586842	103	43	58	59	65
58	H	-4.348155	2.944878	2.529318	6	57			
59	H	-4.088467	3.608965	0.936611	6	57			
60	C	2.791889	0.169415	2.956684	209	45	61	105	
61	H	2.562610	0.065690	1.890490	10	60			
62	C	4.309955	-2.217789	1.650806	103	30	47	63	64
63	H	4.601544	-1.842349	2.632796	6	62			
64	H	4.963182	-3.081487	1.442397	6	62			
65	C	-4.197306	1.456513	0.950920	311	57	72	73	
66	C	2.771842	2.827828	1.275452	209	46	67	103	
67	H	2.448844	2.062081	0.558816	10	66			
68	C	4.975671	2.655783	0.471273	103	6	46	69	70
69	H	5.051699	3.330492	-0.382362	6	68			
70	H	5.940742	2.712191	0.987540	6	68			
71	C	-4.749638	0.139833	-1.019781	311	10	48	72	
72	C	-4.429131	1.376904	-0.432780	311	20	65	71	
73	C	-4.268251	0.297762	1.744435	311	49	65	82	
74	C	-1.934392	3.044995	2.947680	209	43	75	100	
75	H	-2.623776	3.166328	3.799890	10	74			
76	C	-4.304466	-2.239473	1.936955	103	44	49	77	78
77	H	-5.236552	-2.818058	1.916475	6	76			
78	H	-4.081208	-2.031895	2.989434	6	76			
79	N	-2.991507	0.052979	3.989734	214	80	82		
80	C	-1.975656	-0.357987	3.356024	209	79	81	85	
81	H	-1.953237	-0.443912	2.259292	10	80			
82	C	-4.211158	0.386960	3.266493	103	73	79	83	84
83	H	-4.991632	-0.261341	3.683725	6	82			
84	H	-4.494748	1.388297	3.596989	6	82			
85	C	-0.735479	-0.799827	4.083156	3	80	86	87	115
86	H	-0.728158	-0.379681	5.093474	6	85			
87	H	-0.792737	-1.894761	4.196628	6	85			
88	C	-1.199797	-4.298319	1.384929	3	50	89	90	117
89	H	-1.199355	-5.277107	1.883356	6	88			
90	H	-1.475851	-4.455286	0.334967	6	88			
91	C	-1.839416	-2.896836	-2.804179	3	24	92	93	119
92	H	-2.107352	-3.868373	-3.229994	6	91			
93	H	-1.879861	-2.167202	-3.629649	6	91			
94	C	-1.681862	0.822717	-3.975177	3	18	95	96	121
95	H	-1.819408	0.335975	-4.945811	6	94			
96	H	-1.735298	1.907315	-4.153361	6	94			
97	C	-1.208471	4.746550	-1.314826	3	13	98	99	123
98	H	-0.849778	4.973575	-0.301498	6	97			
99	H	-1.295324	5.718502	-1.825718	6	97			
100	C	-0.478862	3.091213	3.312637	3	74	101	102	125
101	H	-0.341751	2.335528	4.103736	6	100			
102	H	-0.261543	4.053703	3.795496	6	100			
103	C	1.740766	3.319755	2.189358	801	66	104	125	
104	H	2.061789	4.063538	2.916768	811	103			

105	C	1.647690	-0.024227	3.838330	801	60	106	115
106	H	1.774857	0.169134	4.901458	811	105		
107	C	1.230780	-4.317535	1.998254	801	55	108	117
108	H	1.102083	-5.303441	2.444449	811	107		
109	C	0.501839	-3.762359	-2.586235	801	32	110	119
110	H	0.258643	-4.597385	-3.240306	811	109		
111	C	0.670295	-0.091964	-3.971599	801	38	112	121
112	H	0.618115	-0.399209	-5.014262	811	111		
113	C	1.065762	3.793001	-1.635365	801	8	114	123
114	H	1.403539	4.299184	-0.732866	811	113		
115	C	0.507508	-0.480732	3.317906	8	85	105	116
116	H	0.479982	-0.668982	2.243802	10	115		
117	C	0.178626	-3.695853	1.453405	8	88	107	118
118	H	0.325593	-2.732916	0.967416	10	117		
119	C	-0.442036	-2.867696	-2.270284	8	91	109	120
120	H	-0.178451	-2.021990	-1.637559	10	119		
121	C	-0.358465	0.497760	-3.356017	8	94	111	122
122	H	-0.239607	0.792195	-2.310946	10	121		
123	C	-0.206275	3.891466	-2.030583	8	97	113	124
124	H	-0.544014	3.375105	-2.926822	10	123		
125	C	0.507914	2.801700	2.221206	8	100	103	126
126	H	0.216490	2.050865	1.488139	10	125		
127	F	1.032339	-0.899725	-0.190088	401	128		
128	H	1.581904	-1.646165	-0.520476	402	127		

HCN@SP2

```
     129  molden generated tinker .xyz
  1  N    2.587825   -3.132167   -0.905993     214   15   32
  2  N    3.387129    2.676610   -1.936094     214  8   34
  3  N   -3.608998   -0.533875   -3.691562     214   10   18
  4  N   -3.867538   -3.365039   -1.315104     214   24   26
  5  N   -2.744725    2.568651   -2.343075     214   13   20
  6  C    4.704007    1.195338    0.200981     311  7   29   68
  7  C    4.586413    0.744765   -1.124011     311  6   31   34
  8  C    2.306900    2.649642   -2.607870     209  2    9  113
  9  H    2.162581    2.001183   -3.485234      10  8
 10  C   -4.716067   -0.665076   -2.750893     103  3   11   12   71
 11  H   -5.016439   -1.713294   -2.790812       6   10
 12  H   -5.555936   -0.109057   -3.187872       6   10
 13  C   -2.492895    3.804968   -2.263793     209  5   14   97
 14  H   -3.289123    4.544695   -2.071160      10   13
 15  C    4.014411   -2.996120   -0.635652     103  1   16   17   23
 16  H    4.619680   -3.377191   -1.468767       6   15
 17  H    4.254465   -3.623589    0.223898       6   15
 18  C   -2.458356   -0.239302   -3.255566     209  3   19   94
 19  H   -2.235599   -0.082470   -2.193525      10   18
 20  C   -4.134934    2.156214   -2.164038     103  5   21   22   72
 21  H   -4.452436    1.704381   -3.104732       6   20
 22  H   -4.800173    3.019108   -1.996005       6   20
 23  C    4.337318   -1.538101   -0.334289     311   15   30   31
 24  C   -2.654695   -3.000042   -1.312556     209  4   25   91
 25  H   -2.288850   -2.106992   -0.787738      10   24
 26  C   -4.870260   -2.604672   -0.580473     103  4   27   28   48
 27  H   -5.055388   -3.173596    0.330705       6   26
 28  H   -5.798002   -2.669116   -1.161013       6   26
 29  C    4.638254    0.274244    1.261608     311  6   30   52
 30  C    4.384330   -1.082437    0.993906     311   23   29   62
 31  C    4.499823   -0.629921   -1.396127     311  7   23   40
 32  C    2.223902   -3.591854   -2.036960     209  1   33  109
 33  H    2.964470   -3.887494   -2.792956      10   32
 34  C    4.465915    1.760590   -2.263231     103  2    7   35   36
 35  H    5.390650    2.343670   -2.359216       6   34
 36  H    4.297665    1.257892   -3.222589       6   34
 37  N    3.411539   -1.165853   -3.672461     214   38   40
 38  C    2.328488   -0.677299   -3.216209     209   37   39  111
 39  H    2.255811   -0.240630   -2.209818      10   38
 40  C    4.603807   -1.121620   -2.835756     103   31   37   41   42
 41  H    5.337777   -0.509113   -3.372926       6   40
 42  H    5.034884   -2.126322   -2.857886       6   40
 43  N   -2.490876    3.193970    0.948961     214   57   74
 44  N   -3.453120   -2.728755    1.978877     214   50   76
 45  N    3.734004    0.754557    3.634830     214   52   60
 46  N    3.857029    3.364002    1.239400     214   66   68
 47  N    2.696909   -2.371482    2.199220     214   55   62
 48  C   -4.592677   -1.140235   -0.261050     311   26   49   71
 49  C   -4.485475   -0.705296    1.067992     311   48   73   76
 50  C   -2.534514   -2.839579    2.840531     209   44   51   88
```

51	H	-2.494337	-2.203333	3.738688	10	50		
52	C	4.852945	0.738915	2.701934	103	29	45	53	54
53	H	5.252408	1.755071	2.717042	6	52		
54	H	5.634838	0.117853	3.157974	6	52		
55	C	2.362016	-3.595372	2.150302	209	47	56	107
56	H	3.116149	-4.393841	2.055935	10	55		
57	C	-3.904438	3.041572	0.627411	103	43	58	59	65
58	H	-4.553666	3.414630	1.433353	6	57		
59	H	-4.117477	3.669574	-0.236662	6	57		
60	C	2.555836	0.583948	3.190494	209	45	61	105
61	H	2.338815	0.382368	2.134767	10	60		
62	C	4.116388	-2.049893	2.141309	103	30	47	63	64
63	H	4.373256	-1.584521	3.094112	6	62		
64	H	4.744224	-2.952077	2.051948	6	62		
65	C	-4.207442	1.584548	0.308468	311	57	72	73
66	C	2.651985	2.985933	1.072994	209	46	67	103
67	H	2.369593	2.139334	0.433934	10	66		
68	C	4.915975	2.672942	0.511980	103	6	46	69	70
69	H	5.080182	3.241603	-0.404476	6	68		
70	H	5.834725	2.782284	1.099262	6	68		
71	C	-4.518525	-0.207359	-1.308571	311	10	48	72
72	C	-4.283279	1.150467	-1.025596	311	20	65	71
73	C	-4.363813	0.663097	1.359080	311	49	65	82
74	C	-2.199756	3.632933	2.099924	209	43	75	100
75	H	-2.977905	3.913247	2.829198	10	74		
76	C	-4.491841	-1.728817	2.197596	103	44	49	77	78
77	H	-5.452379	-2.259701	2.219300	6	76		
78	H	-4.376066	-1.242381	3.171953	6	76		
79	N	-3.370759	1.038664	3.727821	214	80	82	
80	C	-2.281327	0.529062	3.332842	209	79	81	85
81	H	-2.126506	0.181842	2.300226	10	80		
82	C	-4.493799	1.138673	2.803954	103	73	79	83	84
83	H	-5.321080	0.597449	3.279220	6	82		
84	H	-4.813771	2.182159	2.830766	6	82		
85	C	-1.125730	0.332772	4.280334	3	80	86	87	115
86	H	-1.173348	1.067618	5.090560	6	85		
87	H	-1.255883	-0.650893	4.760699	6	85		
88	C	-1.466054	-3.888635	2.716047	3	50	89	90	117
89	H	-1.553096	-4.544808	3.596131	6	88		
90	H	-1.682859	-4.500357	1.834484	6	88		
91	C	-1.653505	-3.798656	-2.118161	3	24	92	93	119
92	H	-2.006622	-4.837512	-2.137381	6	91		
93	H	-1.689530	-3.439369	-3.155503	6	91		
94	C	-1.319510	0.020410	-4.206319	3	18	95	96	121
95	H	-1.383820	-0.635201	-5.080187	6	94		
96	H	-1.459283	1.051314	-4.565205	6	94		
97	C	-1.121754	4.406185	-2.372029	3	13	98	99	123
98	H	-0.890771	4.848601	-1.393242	6	97		
99	H	-1.187281	5.251043	-3.075406	6	97		
100	C	-0.791960	3.762818	2.606351	3	74	101	102	125
101	H	-0.764942	3.221789	3.566287	6	100		
102	H	-0.597349	4.812149	2.867596	6	100		
103	C	1.557128	3.649968	1.778119	801	66	104	125
104	H	1.842102	4.486579	2.414326	811	103		

105	C	1.386194	0.628801	4.062924	801	60	106	115
106	H	1.541470	0.896453	5.105928	811	105		
107	C	0.966973	-4.054286	2.178776	801	55	108	117
108	H	0.795327	-5.058600	1.792043	811	107		
109	C	0.818278	-3.681669	-2.447549	801	32	110	119
110	H	0.654942	-3.685996	-3.524093	811	109		
111	C	1.097041	-0.664591	-3.994799	801	38	112	121
112	H	1.115124	-1.148910	-4.969098	811	111		
113	C	1.182572	3.485724	-2.191910	801	8	114	123
114	H	1.366464	4.104457	-1.315511	811	113		
115	C	0.184807	0.348859	3.554470	8	85	105	116
116	H	0.135413	0.090636	2.497603	10	115		
117	C	-0.058048	-3.349241	2.667352	8	88	107	118
118	H	0.139745	-2.361540	3.083888	10	117		
119	C	-0.235731	-3.737752	-1.625082	8	91	109	120
120	H	-0.071091	-3.773724	-0.547467	10	119		
121	C	0.000955	-0.069901	-3.516395	8	94	111	122
122	H	0.047679	0.407659	-2.536171	10	121		
123	C	-0.010397	3.494288	-2.793072	8	97	113	124
124	H	-0.190820	2.857211	-3.657716	10	123		
125	C	0.293145	3.215519	1.725355	8	100	103	126
126	H	0.025685	2.373606	1.087165	10	125		
127	C	0.611430	-1.050799	0.025919	501	128	129	
128	N	-0.218616	-0.253268	0.142177	502	127		
129	H	1.384360	-1.815963	-0.113578	503	127		

NH_3@SP2

```
      130  molden generated tinker .xyz
  1  N    -2.652816    3.244838   -0.636550    214   15   32
  2  N    -3.212076   -2.500457   -2.223791    214    8   34
  3  N     3.739297    0.827966   -3.601644    214   10   18
  4  N     3.747975    3.414073   -1.156218    214   24   26
  5  N     2.888695   -2.370782   -2.463793    214   13   20
  6  C    -4.644473   -1.270441   -0.014373    311    7   29     68
  7  C    -4.487258   -0.687282   -1.283924    311    6   31     34
  8  C    -2.117099   -2.408868   -2.864281    209    2    9  113
  9  H    -1.960943   -1.692881   -3.685965     10    8
 10  C     4.814787    0.897356   -2.617922    103    3   11     12   71
 11  H     5.116127    1.945652   -2.587036      6   10
 12  H     5.668696    0.366048   -3.057583      6   10
 13  C     2.653980   -3.612765   -2.500883    209    5   14     97
 14  H     3.454073   -4.356684   -2.345075     10   13
 15  C    -4.070764    3.001928   -0.416995    103    1   16     17   23
 16  H    -4.691355    3.437374   -1.213235      6   15
 17  H    -4.362939    3.520524    0.497903      6   15
 18  C     2.582513    0.481162   -3.227850    209    3   19     94
 19  H     2.343669    0.221887   -2.189300     10   18
 20  C     4.265637   -1.957289   -2.205335    103    5   21     22   72
 21  H     4.615304   -1.451081   -3.106146      6   20
 22  H     4.933654   -2.821520   -2.057891      6   20
 23  C    -4.329020    1.510674   -0.268552    311   15   30     31
 24  C     2.553322    2.996429   -1.136291    209    4   25     91
 25  H     2.233250    2.127702   -0.541188     10   24
 26  C     4.764098    2.731578   -0.357414    103    4   27     28   48
 27  H     4.828885    3.280229    0.581541      6   26
 28  H     5.721785    2.888477   -0.866953      6   26
 29  C    -4.627999   -0.458230    1.134032    311    6   30     52
 30  C    -4.399125    0.923719    1.006658    311   23   29     62
 31  C    -4.423938    0.708941   -1.419000    311    7   23     40
 32  C    -2.315404    4.027742   -1.579695    209    1   33  109
 33  H    -3.067821    4.526889   -2.210437     10   32
 34  C    -4.298178   -1.580990   -2.510266    103    2    7     35   36
 35  H    -5.202266   -2.172000   -2.704043      6   34
 36  H    -4.101110   -0.982536   -3.407457      6   34
 37  N    -3.269930    1.509422   -3.589835    214   38   40
 38  C    -2.182398    1.032355   -3.137509    209   37   39  111
 39  H    -2.120544    0.530402   -2.164182     10   38
 40  C    -4.487318    1.343981   -2.805370    103   31   37     41   42
 41  H    -5.179786    0.770636   -3.433064      6   40
 42  H    -4.947628    2.333375   -2.742068      6   40
 43  N     2.493622   -3.161802    0.809929    214   57   74
 44  N     3.336927    2.648627    2.229161    214   50   76
 45  N    -3.794315   -1.140057    3.488495    214   52   60
 46  N    -3.833718   -3.542140    0.836749    214   66   68
 47  N    -2.751482    2.099830    2.356277    214   55   62
 48  C     4.572561    1.238296   -0.112563    311   26   49     71
 49  C     4.447224    0.726933    1.188027    311   48   73     76
 50  C     2.461371    2.707638    3.140621    209   44   51     88
```

51	H	2.488690	2.042898	4.018586	10	50			
52	C	-4.878873	-1.066167	2.516327	103	29	45	53	54
53	H	-5.250585	-2.088388	2.417412	6	52			
54	H	-5.694251	-0.509088	2.996080	6	52			
55	C	-2.414769	3.322634	2.322589	209	47	56	107	
56	H	-3.164545	4.124543	2.225352	10	55			
57	C	3.913677	-2.993643	0.521317	103	43	58	59	65
58	H	4.549096	-3.418290	1.312739	6	57			
59	H	4.137193	-3.569405	-0.375827	6	57			
60	C	-2.608222	-0.907960	3.092674	209	45	61	105	
61	H	-2.365141	-0.624410	2.065121	10	60			
62	C	-4.168585	1.776828	2.250117	103	30	47	63	64
63	H	-4.436936	1.217815	3.147466	6	62			
64	H	-4.802480	2.678588	2.236278	6	62			
65	C	4.226474	-1.521661	0.294999	311	57	72	73	
66	C	-2.631007	-3.128735	0.783887	209	46	67	103	
67	H	-2.340401	-2.208682	0.265475	10	66			
68	C	-4.860545	-2.774027	0.139184	103	6	46	69	70
69	H	-4.988137	-3.247565	-0.835683	6	68			
70	H	-5.802822	-2.938151	0.674295	6	68			
71	C	4.568921	0.360503	-1.210369	311	10	48	72	
72	C	4.352440	-1.014445	-1.009201	311	20	65	71	
73	C	4.340030	-0.658308	1.399166	311	49	65	82	
74	C	2.187560	-3.709938	1.909855	209	43	75	100	
75	H	2.958508	-4.056495	2.618203	10	74			
76	C	4.421086	1.684227	2.373437	103	44	49	77	78
77	H	5.357602	2.255493	2.409629	6	76			
78	H	4.345771	1.140367	3.321123	6	76			
79	N	3.294108	-1.185371	3.718802	214	80	82		
80	C	2.208298	-0.655917	3.341021	209	79	81	85	
81	H	2.073746	-0.232150	2.334369	10	80			
82	C	4.437521	-1.219385	2.816154	103	73	79	83	84
83	H	5.251427	-0.705560	3.341890	6	82			
84	H	4.763396	-2.260836	2.784821	6	82			
85	C	1.027784	-0.539604	4.269128	3	80	86	87	115
86	H	1.090434	-1.301581	5.052790	6	85			
87	H	1.111847	0.431239	4.785263	6	85			
88	C	1.359746	3.729064	3.102637	3	50	89	90	117
89	H	1.434100	4.314893	4.032404	6	88			
90	H	1.548606	4.410665	2.267876	6	88			
91	C	1.511465	3.626865	-2.026824	3	24	92	93	119
92	H	1.782861	4.667154	-2.231672	6	91			
93	H	1.584007	3.097434	-2.991077	6	91			
94	C	1.451506	0.326228	-4.210996	3	18	95	96	121
95	H	1.533998	1.065684	-5.013316	6	94			
96	H	1.574369	-0.665603	-4.670932	6	94			
97	C	1.293910	-4.210578	-2.712728	3	13	98	99	123
98	H	1.030144	-4.744647	-1.789648	6	97			
99	H	1.389392	-4.984073	-3.490525	6	97			
100	C	0.775656	-3.900158	2.385367	3	74	101	102	125
101	H	0.724387	-3.422380	3.377665	6	100			
102	H	0.602621	-4.968454	2.574599	6	100			
103	C	-1.543869	-3.834441	1.459024	801	66	104	125	
104	H	-1.807340	-4.762019	1.964591	811	103			

105	C	-1.454136	-0.971444	3.985032	801	60	106	115
106	H	-1.606939	-1.328495	5.001786	811	105		
107	C	-1.020503	3.782229	2.372419	801	55	108	117
108	H	-0.824932	4.727498	1.867473	811	107		
109	C	-0.911439	4.288667	-1.928893	801	32	110	119
110	H	-0.724670	5.135147	-2.587611	811	109		
111	C	-0.932440	1.104219	-3.889634	801	38	112	121
112	H	-0.915945	1.737573	-4.773834	811	111		
113	C	-0.998641	-3.271608	-2.487496	801	8	114	123
114	H	-1.195404	-3.940441	-1.651611	811	113		
115	C	-0.267450	-0.576724	3.516256	8	85	105	116
116	H	-0.234756	-0.235822	2.479001	10	115		
117	C	-0.032093	3.156933	3.015458	8	88	107	118
118	H	-0.251230	2.227984	3.541777	10	117		
119	C	0.094000	3.490473	-1.555741	8	91	109	120
120	H	-0.150775	2.632587	-0.931222	10	119		
121	C	0.127010	0.379813	-3.519806	8	94	111	122
122	H	0.041824	-0.261867	-2.639710	10	121		
123	C	0.196450	-3.261375	-3.083561	8	97	113	124
124	H	0.389709	-2.578359	-3.909684	10	123		
125	C	-0.310852	-3.320217	1.530407	8	100	103	126
126	H	-0.090645	-2.377820	1.029756	10	125		
127	N	-0.907471	0.238337	0.281548	423	128	129	130
128	H	-1.275718	1.121550	0.637716	424	127		
129	H	-1.665627	-0.173816	-0.258317	424	127		
130	H	-0.162972	0.454103	-0.376530	424	127		

MeOH@SP2

```
        132  molden generated tinker .xyz
   1  N    -2.556658   3.420341  -0.413424    214   15   32
   2  N    -3.565011  -2.342141  -2.165096    214  8   34
   3  N     3.510591   0.822837  -3.739422    214   10   18
   4  N     3.826012   3.325847  -1.264168    214   24   26
   5  N     2.473928  -2.436331  -2.496265    214   13   20
   6  C    -4.716591  -1.057181   0.188654    311  7   29   68
   7  C    -4.617264  -0.448132  -1.071077    311  6   31   34
   8  C    -2.467234  -2.251691  -2.800343    209  2    9  113
   9  H    -2.256868  -1.448569  -3.522919     10  8
  10  C     4.616749   0.781364  -2.790791    103  3   11   12   71
  11  H     5.005289   1.800219  -2.754316      6   10
  12  H     5.408304   0.189425  -3.269152      6   10
  13  C     2.237180  -3.654988  -2.749401    209  5   14   97
  14  H     3.055311  -4.388882  -2.835739     10   13
  15  C    -3.968092   3.195228  -0.141976    103  1   16   17   23
  16  H    -4.610958   3.659691  -0.902600      6   15
  17  H    -4.211897   3.700674   0.795435      6   15
  18  C     2.339273   0.551072  -3.347405    209  3   19   94
  19  H     2.106075   0.274068  -2.312215     10   18
  20  C     3.874320  -2.031546  -2.380801    103  5   21   22   72
  21  H     4.116248  -1.501864  -3.302688      6   20
  22  H     4.547135  -2.903085  -2.340760      6   20
  23  C    -4.259045   1.707681  -0.011249    311   15   30   31
  24  C     2.603169   3.015176  -1.155526    209  4   25   91
  25  H     2.252346   2.197669  -0.511503     10   24
  26  C     4.820816   2.570496  -0.503371    103  4   27   28   48
  27  H     4.955349   3.100990   0.438218      6   26
  28  H     5.769678   2.662888  -1.044703      6   26
  29  C    -4.549270  -0.285769   1.354039    311  6   30   52
  30  C    -4.241842   1.082574   1.249845    311   23   29   62
  31  C    -4.495286   0.947875  -1.169261    311  7   23   40
  32  C    -2.242378   4.181892  -1.380992    209  1   33  109
  33  H    -3.007267   4.678252  -1.998844     10   32
  34  C    -4.572182  -1.307138  -2.336658    103  2    7   35   36
  35  H    -5.537695  -1.800717  -2.506611      6   34
  36  H    -4.362705  -0.686640  -3.215450      6   34
  37  N    -3.491200   1.790367  -3.393919    214   38   40
  38  C    -2.383389   1.277528  -3.035167    209   37   39  111
  39  H    -2.258459   0.739822  -2.084041     10   38
  40  C    -4.650984   1.623794  -2.528116    103   31   37   41   42
  41  H    -5.398583   1.078854  -3.116816      6   40
  42  H    -5.090776   2.616980  -2.403747      6   40
  43  N     2.290963  -3.253223   0.657075    214   57   74
  44  N     3.580676   2.526582   2.178844    214   50   76
  45  N    -3.554286  -1.192167   3.564082    214   52   60
  46  N    -4.047559  -3.379200   0.989914    214   66   68
  47  N    -2.422931   2.105799   2.540393    214   55   62
  48  C     4.536241   1.089030  -0.267332    311   26   49   71
  49  C     4.473718   0.555457   1.031373    311   48   73   76
  50  C     2.801630   2.661250   3.166823    209   44   51   88
```

51	H	2.863415	2.008930	4.053491	10	50			
52	C	-4.719206	-0.938830	2.725431	103	29	45	53	54
53	H	-5.212691	-1.907072	2.621748	6	52			
54	H	-5.407390	-0.331885	3.328144	6	52			
55	C	-2.016688	3.298185	2.397966	209	47	56	107	
56	H	-2.717624	4.136113	2.260973	10	55			
57	C	3.702248	-3.120465	0.316103	103	43	58	59	65
58	H	4.352151	-3.588621	1.068842	6	57			
59	H	3.868766	-3.678900	-0.605453	6	57			
60	C	-2.398052	-0.898002	3.129912	209	45	61	105	
61	H	-2.221736	-0.443844	2.149137	10	60			
62	C	-3.866428	1.879318	2.497604	103	30	47	63	64
63	H	-4.125016	1.316269	3.395481	6	62			
64	H	-4.433031	2.823056	2.543493	6	62			
65	C	4.057486	-1.655104	0.115126	311	57	72	73	
66	C	-2.818650	-3.089742	0.824006	209	46	67	103	
67	H	-2.477587	-2.243963	0.213447	10	66			
68	C	-5.039891	-2.542545	0.320704	103 6	46	69	70	
69	H	-5.224588	-2.988221	-0.657685	6	68			
70	H	-5.976579	-2.644590	0.881708	6	68			
71	C	4.395205	0.239673	-1.378372	311	10	48	72	
72	C	4.107140	-1.122462	-1.182738	311	20	65	71	
73	C	4.297460	-0.825739	1.226605	311	49	65	82	
74	C	2.012453	-3.828012	1.751769	209	43	75	100	
75	H	2.802960	-4.210826	2.416907	10	74			
76	C	4.602298	1.489140	2.230238	103	44	49	77	78
77	H	5.577278	1.992497	2.204155	6	76			
78	H	4.560725	0.932858	3.173893	6	76			
79	N	3.352864	-1.445189	3.570754	214	80	82		
80	C	2.370551	-0.667474	3.391617	209	79	81	85	
81	H	2.294573	-0.007883	2.517158	10	80			
82	C	4.458256	-1.439833	2.616450	103	73	79	83	84
83	H	5.298250	-0.954406	3.128222	6	82			
84	H	4.775704	-2.479069	2.520890	6	82			
85	C	1.235486	-0.571170	4.376617	3	80	86	87	115
86	H	1.264369	-1.423507	5.061877	6	85			
87	H	1.401151	0.334685	4.983365	6	85			
88	C	1.756736	3.741891	3.192865	3	50	89	90	117
89	H	1.914367	4.334232	4.107153	6	88			
90	H	1.921850	4.398106	2.333546	6	88			
91	C	1.553846	3.706310	-1.990481	3	24	92	93	119
92	H	1.839938	4.748786	-2.163311	6	91			
93	H	1.585244	3.215980	-2.976869	6	91			
94	C	1.170475	0.523511	-4.299620	3	18	95	96	121
95	H	1.278772	1.294847	-5.068871	6	94			
96	H	1.211828	-0.447770	-4.817492	6	94			
97	C	0.855673	-4.214697	-2.934915	3	13	98	99	123
98	H	0.619701	-4.824983	-2.051977	6	97			
99	H	0.901678	-4.917503	-3.780599	6	97			
100	C	0.616826	-3.997893	2.274781	3	74	101	102	125
101	H	0.598717	-3.474412	3.245158	6	100			
102	H	0.452523	-5.056276	2.519141	6	100			
103	C	-1.765050	-3.829980	1.515942	801	66	104	125	
104	H	-2.086085	-4.630639	2.180366	811	103			

105	C	-1.197192	-1.120620	3.939398	801	60	106	115	
106	H	-1.267952	-1.822109	4.768494	811	105			
107	C	-0.598286	3.688907	2.373311	801	55	108	117	
108	H	-0.352896	4.469013	1.652760	811	107			
109	C	-0.844841	4.421061	-1.771848	801	32	110	119	
110	H	-0.657898	5.291364	-2.399161	811	109			
111	C	-1.192034	1.342784	-3.878242	801	38	112	121	
112	H	-1.236046	1.964516	-4.770152	811	111			
113	C	-1.408458	-3.230242	-2.556071	801	8	114	123	
114	H	-1.632650	-3.981981	-1.801529	811	113			
115	C	-0.077826	-0.449773	3.664234	8	85	105	116	
116	H	-0.113931	0.275149	2.851564	10	115			
117	C	0.343387	3.220268	3.193062	8	88	107	118	
118	H	0.075178	2.468431	3.934996	10	117			
119	C	0.149697	3.577967	-1.476165	8	91	109	120	
120	H	-0.092300	2.690925	-0.890718	10	119			
121	C	-0.120849	0.615871	-3.550103	8	94	111	122	
122	H	-0.164075	0.013237	-2.639789	10	121			
123	C	-0.225070	-3.203026	-3.171733	8	97	113	124	
124	H	-0.014453	-2.424315	-3.903951	10	123			
125	C	-0.486311	-3.452561	1.418486	8	100	103	126	
126	H	-0.228217	-2.654118	0.725999	10	125			
127	O	0.235262	-1.045219	-0.861126	38	128	129		
128	H	0.948968	-1.638250	-1.157094	39	127			
129	C	0.715534	-0.255963	0.200775	41	127	130	131	132
130	H	-0.047898	0.496871	0.423109	40	129			
131	H	0.885878	-0.862037	1.097595	40	129			
132	H	1.659528	0.250306	-0.045995	40	129			

H_2O@SP3

```
        141  molden generated tinker .xyz
   1  N     2.499490    3.128555    1.329490      214 15   32
   2  N     3.547587   -2.872188    1.628678      214  8   34
   3  N    -3.869960    0.011731    3.652280      214 10   18
   4  N    -4.028233    3.093083    1.658626      214 24   26
   5  N    -2.711870   -2.745991    1.815278      214 13   20
   6  C     4.793080   -1.014545   -0.303193      311  7   29   68
   7  C     4.618827   -0.762668    1.066293      311  6   31   34
   8  C     2.616612   -3.154231    2.438256      209  2    9   94
   9  H     2.574132   -2.722029    3.451379       10  8
  10  C    -4.955527    0.224621    2.697867      103  3   11   12   71
  11  H    -5.311721    1.240943    2.874960        6 10
  12  H    -5.777893   -0.430707    3.013031        6 10
  13  C    -2.372958   -3.962897    1.722089      209  5   14  109
  14  H    -3.133094   -4.750673    1.585086       10 13
  15  C     3.932516    2.996618    1.089000      103  1   16   17   23
  16  H     4.521636    3.265855    1.977689        6 15
  17  H     4.199304    3.731753    0.328653        6 15
  18  C    -2.684978   -0.052212    3.211815      209  3   19  103
  19  H    -2.455803    0.053034    2.144428       10 18
  20  C    -4.138741   -2.427447    1.740536      103  5   21   22   72
  21  H    -4.444593   -2.123389    2.741968        6 20
  22  H    -4.738516   -3.315693    1.486464        6 20
  23  C     4.284175    1.598737    0.602928      311 15   30   31
  24  C    -2.813008    2.755988    1.526636      209  4   25   97
  25  H    -2.476357    1.986439    0.817114       10 24
  26  C    -5.042367    2.465184    0.817904      103  4   27   28   48
  27  H    -5.221686    3.157068   -0.005562        6 26
  28  H    -5.971861    2.450000    1.398518        6 26
  29  C     4.725537    0.047464   -1.221184      311  6   30   52
  30  C     4.420182    1.345191   -0.772503      311 23   29   62
  31  C     4.438809    0.551694    1.529246      311  7   23   40
  32  C     2.137438    3.536849    2.472156      209  1   33  118
  33  H     2.870932    3.763564    3.264538       10 32
  34  C     4.583837   -1.934859    2.042360      103  2    7   35   36
  35  H     5.540816   -2.471835    2.023164        6 34
  36  H     4.430861   -1.592991    3.072226        6 34
  37  N     3.305663    0.601813    3.860634      214 38   40
  38  C     2.270611    0.079245    3.350533      209 37   39   88
  39  H     2.202924   -0.178433    2.282708       10 38
  40  C     4.478994    0.835736    3.027704      103 31   37   41   42
  41  H     5.288657    0.248161    3.477746        6 40
  42  H     4.773875    1.871471    3.207546        6 40
  43  N    -2.432581   -2.975882   -1.429149      214 57   74
  44  N    -3.492504    2.914967   -1.612513      214 50   76
  45  N     3.863567   -0.045111   -3.641237      214 52   60
  46  N     4.096428   -3.069794   -1.662367      214 66   68
  47  N     2.802851    2.810666   -1.904987      214 55   62
  48  C    -4.763305    1.059503    0.298546      311 26   49   71
  49  C    -4.598348    0.817971   -1.074210      311 48   73   76
  50  C    -2.532075    3.167719   -2.397939      209 44   51   91
```

51	H	-2.472955	2.725063	-3.405733	10	50			
52	C	4.972703	-0.206615	-2.705720	103	29	45	53	54
53	H	5.351820	-1.216713	-2.869239	6	52			
54	H	5.771865	0.460612	-3.053952	6	52			
55	C	2.461408	4.010153	-1.698201	209	47	56	112	
56	H	3.205884	4.784342	-1.441211	10	55			
57	C	-3.864583	-2.921805	-1.131474	103	43	58	59	65
58	H	-4.466079	-3.225864	-1.998950	6	57			
59	H	-4.056507	-3.671008	-0.361902	6	57			
60	C	2.699655	0.137644	-3.178425	209	45	61	106	
61	H	2.488820	0.178187	-2.103292	10	60			
62	C	4.217980	2.474783	-1.778487	103	30	47	63	64
63	H	4.558413	2.161713	-2.766231	6	62			
64	H	4.827555	3.351426	-1.503751	6	62			
65	C	-4.262966	-1.543157	-0.629845	311	57	72	73	
66	C	2.880855	-2.724709	-1.572938	209	46	67	100	
67	H	2.532291	-1.939862	-0.886315	10	66			
68	C	5.090635	-2.422071	-0.810853	103	6	46	69	70
69	H	5.265832	-3.108280	0.017904	6	68			
70	H	6.027083	-2.402356	-1.379877	6	68			
71	C	-4.692345	-0.007780	1.209777	311	10	48	72	
72	C	-4.384206	-1.299339	0.747453	311	20	65	71	
73	C	-4.435477	-0.493743	-1.547273	311	49	65	82	
74	C	-2.064753	-3.551177	-2.497293	209	43	75	115	
75	H	-2.804148	-3.963075	-3.202557	10	74			
76	C	-4.538845	1.996633	-2.042260	103	44	49	77	78
77	H	-5.487901	2.547603	-2.029139	6	76			
78	H	-4.381335	1.656804	-3.072412	6	76			
79	N	-3.293159	-0.571652	-3.862889	214	80	82		
80	C	-2.260527	-0.058793	-3.336556	209	79	81	85	
81	H	-2.206297	0.202619	-2.268562	10	80			
82	C	-4.480869	-0.776018	-3.044063	103	73	79	83	84
83	H	-5.271581	-0.166559	-3.498246	6	82			
84	H	-4.804045	-1.804240	-3.223910	6	82			
85	C	-1.033976	0.238920	-4.145072	3	80	86	87	124
86	H	-1.064772	-0.335920	-5.076787	6	85			
87	H	-1.051066	1.301567	-4.431091	6	85			
88	C	1.058066	-0.237653	4.176075	3	38	89	90	121
89	H	1.054360	-1.316828	4.392790	6	88			
90	H	1.128782	0.279034	5.138734	6	88			
91	C	-1.424805	4.102350	-2.007380	3	50	92	93	130
92	H	-1.429788	4.948003	-2.711222	6	91			
93	H	-1.656930	4.500930	-1.013763	6	91			
94	C	1.512872	-4.098080	2.060687	3	8	95	96	127
95	H	1.750536	-4.516254	1.076436	6	94			
96	H	1.501865	-4.927770	2.782638	6	94			
97	C	-1.754822	3.365829	2.409677	3	24	98	99	136
98	H	-2.061214	4.381919	2.681376	6	97			
99	H	-1.757255	2.791279	3.348571	6	97			
100	C	1.836676	-3.332014	-2.473752	3	66	101	102	133
101	H	1.813208	-2.715686	-3.385511	6	100			
102	H	2.168997	-4.327100	-2.787921	6	100			
103	C	-1.503771	-0.336504	4.095023	3	18	104	105	121
104	H	-1.644708	0.129509	5.076425	6	103			

105	H	-1.467176	-1.422886	4.260032	6 103
106	C	1.511424	0.374095	-4.060523	360 107 108 124
107	H	1.474378	1.449140	-4.285928	6 106
108	H	1.638124	-0.151208	-5.013604	6 106
109	C	-0.960043	-4.460681	1.755239	313 110 111 127
110	H	-0.775236	-4.944175	0.784042	6 109
111	H	-0.913634	-5.275013	2.493431	6 109
112	C	1.046373	4.503402	-1.742418	355 113 114 130
113	H	0.989806	5.311878	-2.486973	6 112
114	H	0.855365	4.987571	-0.773605	6 112
115	C	-0.629772	-3.700352	-2.913466	374 116 117 133
116	H	-0.496662	-3.063710	-3.802403	6 115
117	H	-0.489750	-4.725888	-3.282534	6 115
118	C	0.701587	3.732924	2.862639	332 119 120 136
119	H	0.568784	4.782771	3.163346	6 118
120	H	0.543972	3.157380	3.787563	6 118
121	C	-0.215362	0.119084	3.420604	30388 103 122 123
122	H	-0.165136	-0.312811	2.412769	603 121
123	H	-0.248113	1.201391	3.275716	603 121
124	C	0.231198	-0.033964	-3.344206	30385 106 125 126
125	H	0.176518	0.493153	-2.382671	603 124
126	H	0.274610	-1.098105	-3.096490	603 124
127	C	0.129649	-3.430699	2.038730	30394 109 128 129
128	H	-0.073551	-2.943777	3.001139	603 127
129	H	0.107324	-2.644324	1.277021	603 127
130	C	-0.029831	3.457256	-2.017534	30391 112 131 132
131	H	0.163462	2.980134	-2.986232	603 130
132	H	0.029242	2.667611	-1.260365	603 130
133	C	0.431437	-3.366135	-1.863302	303 100 115 134 135
134	H	0.208500	-2.401094	-1.397044	603 133
135	H	0.389347	-4.102959	-1.051489	603 133
136	C	-0.342564	3.343398	1.816852	30397 118 137 138
137	H	-0.279343	4.024022	0.960203	603 136
138	H	-0.109627	2.347371	1.422062	603 136
139	O	-1.118933	-0.837536	0.141587	222 140 141
140	H	-1.526487	-1.276809	0.908640	223 139
141	H	-1.450412	-1.419442	-0.566532	223 139

HF@SP3

```
      140  molden generated tinker .xyz
  1  N     2.468969   -2.909262   -1.371254     214 15   32
  2  N     3.401362    2.954834   -1.648053     214  8   34
  3  N    -3.916316   -0.125301   -3.598322     214 10   18
  4  N    -4.083099   -3.146063   -1.588631     214 24   26
  5  N    -2.894042    2.766204   -1.889233     214 13   20
  6  C     4.749349    1.147249    0.257006     311  7   29   68
  7  C     4.569634    0.892898   -1.111573     311  6   31   34
  8  C     2.430099    3.187174   -2.426217     209  2    9   94
  9  H     2.371429    2.741510   -3.432625      10  8
 10  C    -5.016469   -0.293484   -2.652183     103  3   11   12   71
 11  H    -5.382985   -1.310086   -2.804134       6 10
 12  H    -5.827004    0.359492   -3.000899       6 10
 13  C    -2.568332    3.971639   -1.692091     209  5   14  109
 14  H    -3.321396    4.737038   -1.434140      10 13
 15  C     3.913466   -2.853198   -1.117978     103  1   16   17   23
 16  H     4.477055   -3.158482   -2.007854       6 15
 17  H     4.134174   -3.598263   -0.353866       6 15
 18  C    -2.753316    0.092213   -3.148148     209  3   19  103
 19  H    -2.536066    0.157909   -2.075711      10 18
 20  C    -4.302118    2.408485   -1.747739     103  5   21   22   72
 21  H    -4.647391    2.084405   -2.730308       6 20
 22  H    -4.923304    3.276093   -1.470350       6 20
 23  C     4.307521   -1.469822   -0.641459     311 15   30   31
 24  C    -2.877128   -2.765758   -1.523758     209  4   25   97
 25  H    -2.544177   -1.951383   -0.865262      10 24
 26  C    -5.077417   -2.498692   -0.738185     103  4   27   28   48
 27  H    -5.238184   -3.177327    0.100257       6 26
 28  H    -6.019462   -2.496645   -1.298139       6 26
 29  C     4.726716    0.084157    1.176615     311  6   30   52
 30  C     4.451817   -1.220486    0.731356     311 23   29   62
 31  C     4.430895   -0.425378   -1.571985     311  7   23   40
 32  C     2.078193   -3.536123   -2.404356     209  1   33  118
 33  H     2.816507   -3.982338   -3.086544      10 32
 34  C     4.464980    2.061340   -2.086890     103  2    7   35   36
 35  H     5.399075    2.637546   -2.090760       6 34
 36  H     4.302106    1.710179   -3.112294       6 34
 37  N     3.261530   -0.517449   -3.877456     214 38   40
 38  C     2.207666   -0.075239   -3.328277     209 37   39   88
 39  H     2.136488    0.117397   -2.247974      10 38
 40  C     4.457231   -0.715242   -3.068422     103 31   37   41   42
 41  H     5.240083   -0.102520   -3.531548       6 40
 42  H     4.785530   -1.741902   -3.250378       6 40
 43  N    -2.551580    3.099014    1.343393     214 57   74
 44  N    -3.421347   -2.888972    1.644529     214 50   76
 45  N     3.867813    0.154342    3.595581     214 52   60
 46  N     3.973911    3.180056    1.603108     214 66   68
 47  N     2.866905   -2.730834    1.848257     214 55   62
 48  C    -4.792348   -1.083127   -0.247206     311 26   49   71
 49  C    -4.591039   -0.822643    1.116670     311 48   73   76
 50  C    -2.450364   -3.134318    2.419380     209 44   51   91
```

51	H	-2.386565	-2.697399	3.429161	10	50			
52	C	4.970469	0.344758	2.659266	103	29	45	53	54
53	H	5.322608	1.364805	2.821113	6	52			
54	H	5.788574	-0.300607	3.005687	6	52			
55	C	2.551120	-3.948671	1.718450	209	47	56	112	
56	H	3.313603	-4.724648	1.523596	10	55			
57	C	-3.985813	2.949449	1.114994	103	43	58	59	65
58	H	-4.569926	3.212968	2.008672	6	57			
59	H	-4.269115	3.678353	0.354778	6	57			
60	C	2.708957	-0.075974	3.138971	209	45	61	106	
61	H	2.487156	-0.145129	2.067455	10	60			
62	C	4.274546	-2.360817	1.730599	103	30	47	63	64
63	H	4.599632	-2.042862	2.722022	6	62			
64	H	4.907735	-3.221474	1.456440	6	62			
65	C	-4.322942	1.544338	0.638136	311	57	72	73	
66	C	2.771726	2.787649	1.518289	209	46	67	100	
67	H	2.451448	1.978125	0.846466	10	66			
68	C	4.994772	2.565169	0.761398	103	6	46	69	70
69	H	5.150904	3.252949	-0.070106	6	68			
70	H	5.928747	2.579966	1.334650	6	68			
71	C	-4.760915	-0.024634	-1.171668	311	10	48	72	
72	C	-4.473017	1.280901	-0.733989	311	20	65	71	
73	C	-4.441405	0.498509	1.570792	311	49	65	82	
74	C	-2.185656	3.492605	2.490254	209	43	75	115	
75	H	-2.916292	3.697255	3.291028	10	74			
76	C	-4.478900	-1.990509	2.093237	103	44	49	77	78
77	H	-5.413442	-2.565565	2.109214	6	76			
78	H	-4.304097	-1.639138	3.116480	6	76			
79	N	-3.280004	0.597816	3.883592	214	80	82		
80	C	-2.243311	0.096254	3.356243	209	79	81	85	
81	H	-2.193230	-0.166814	2.288960	10	80			
82	C	-4.472769	0.787897	3.068114	103	73	79	83	84
83	H	-5.254259	0.172071	3.530333	6	82			
84	H	-4.803640	1.813097	3.246884	6	82			
85	C	-1.009008	-0.192527	4.156877	3	80	86	87	124
86	H	-1.027955	0.397462	5.079369	6	85			
87	H	-1.025891	-1.250290	4.461202	6	85			
88	C	0.975661	0.219770	-4.132285	3	38	89	90	121
89	H	0.980760	1.287522	-4.398626	6	88			
90	H	1.015532	-0.334759	-5.076052	6	88			
91	C	-1.337985	-4.059701	2.017327	3	50	92	93	130
92	H	-1.339522	-4.910651	2.715039	6	91			
93	H	-1.573637	-4.453147	1.022516	6	91			
94	C	1.312446	4.106123	-2.028225	3	8	95	96	127
95	H	1.545980	4.506647	-1.035760	6	94			
96	H	1.303531	4.952585	-2.731126	6	94			
97	C	-1.821186	-3.375402	-2.405896	3	24	98	99	136
98	H	-2.125131	-4.390224	-2.683392	6	97			
99	H	-1.811125	-2.791253	-3.338728	6	97			
100	C	1.710735	3.375298	2.411163	3	66	101	102	133
101	H	1.711613	2.773462	3.332921	6	100			
102	H	2.017273	4.383449	2.709715	6	100			
103	C	-1.574409	0.339631	-4.039756	3	18	104	105	121
104	H	-1.712308	-0.170033	-4.999581	6	103			

105	H	-1.538496	1.418430	-4.246854	6	103				
106	C	1.537366	-0.328973	4.038578	3	60	107	108	124	
107	H	1.514396	-1.404298	4.266213	6	106				
108	H	1.672417	0.198649	4.989153	6	106				
109	C	-1.159813	4.481163	-1.747200	3	13	110	111	127	
110	H	-0.966593	4.965495	-0.778947	6	109				
111	H	-1.117199	5.290862	-2.491237	6	109				
112	C	1.139303	-4.455710	1.776112	3	55	113	114	130	
113	H	1.093190	-5.247872	2.538227	6	112				
114	H	0.947450	-4.967740	0.820517	6	112				
115	C	-0.750189	3.688756	2.882577	3	74	116	117	133	
116	H	-0.582496	3.063811	3.773375	6	115				
117	H	-0.629649	4.721672	3.240630	6	115				
118	C	0.649138	-3.709102	-2.816956	3	32	119	120	136	
119	H	0.522884	-4.747659	-3.152749	6	118				
120	H	0.519611	-3.104010	-3.728337	6	118				
121	C	-0.288543	-0.082188	-3.340880	303	88	103	122	123	
122	H	-0.231923	0.412842	-2.362980	603	121				
123	H	-0.327303	-1.152985	-3.125853	603	121				
124	C	0.245987	0.066978	3.336611	303	85	106	125	126	
125	H	0.185785	-0.470559	2.381457	603	124				
126	H	0.286133	1.127703	3.077359	603	124				
127	C	-0.075046	3.446335	-2.030942	303	94	109	128	129	
128	H	-0.269114	2.967782	-2.998689	603	127				
129	H	-0.122190	2.655471	-1.274346	603	127				
130	C	0.055090	-3.410540	2.030891	303	91	112	131	132	
131	H	0.239418	-2.929175	2.999210	603	130				
132	H	0.123742	-2.621256	1.275123	603	130				
133	C	0.297645	3.369320	1.816800	303	100	115	134	135	
134	H	0.072957	2.393391	1.370291	603	133				
135	H	0.226431	4.093743	0.997309	603	133				
136	C	-0.420092	-3.348590	-1.787828	303	97	118	137	138	
137	H	-0.365097	-4.047612	-0.944356	603	136				
138	H	-0.214534	-2.357682	-1.372403	603	136				
139	F	1.106626	-0.925041	-0.172383	401	140				
140	H	1.602398	-1.698908	-0.474728	402	139				

HCN@SP3

```
       141  molden generated tinker .xyz
    1  N    -2.575781   -3.187098    1.117784      214 15   32
    2  N    -3.432612    2.817002    1.855995      214  8   34
    3  N     3.857715   -0.382261    3.647626      214 10   18
    4  N     3.935577   -3.264213    1.456036      214 24   26
    5  N     2.792666    2.600006    2.040925      214 13   20
    6  C    -4.721259    1.140338   -0.214327      311  7   29   68
    7  C    -4.555160    0.777997    1.131037      311  6   31   34
    8  C    -2.488987    3.010100    2.677240      209  2    9   94
    9  H    -2.437081    2.481733    3.643717       10  8
   10  C     4.929131   -0.511068    2.665338      103  3   11   12   71
   11  H     5.280822   -1.540557    2.751233        6 10
   12  H     5.759727    0.110119    3.025192        6 10
   13  C     2.496710    3.830031    2.005671      209  5   14  109
   14  H     3.274798    4.604462    1.887525       10 13
   15  C    -4.001106   -2.994536    0.870716      103  1   16   17   23
   16  H    -4.610395   -3.308515    1.730841        6 15
   17  H    -4.280934   -3.661506    0.054645        6 15
   18  C     2.674029   -0.204852    3.236757      209  3   19  103
   19  H     2.427688   -0.132065    2.171145       10 18
   20  C     4.203065    2.235950    1.926031      103  5   21   22   72
   21  H     4.504155    1.839670    2.896236        6 20
   22  H     4.841468    3.114377    1.736174        6 20
   23  C    -4.303559   -1.551947    0.488431      311 15   30   31
   24  C     2.720679   -2.911808    1.361545      209  4   25   97
   25  H     2.381886   -2.110370    0.691731       10 24
   26  C     4.936086   -2.601513    0.623566      103  4   27   28   48
   27  H     5.069564   -3.236053   -0.252570        6 26
   28  H     5.884862   -2.645697    1.170785        6 26
   29  C    -4.688392    0.149961   -1.211647      311  6   30   52
   30  C    -4.429694   -1.188199   -0.863602      311 23   29   62
   31  C    -4.426576   -0.573917    1.491696      311  7   23   40
   32  C    -2.236499   -3.627341    2.256010      209  1   33  118
   33  H    -2.984573   -3.845785    3.037082       10 32
   34  C    -4.483495    1.866528    2.197814      103  2    7   35   36
   35  H    -5.428478    2.423844    2.234293        6 34
   36  H    -4.328252    1.437848    3.194063        6 34
   37  N    -3.335839   -0.858118    3.828002      214 38   40
   38  C    -2.274086   -0.335473    3.376979      209 37   39   88
   39  H    -2.181696    0.008306    2.335361       10 38
   40  C    -4.503294   -0.970764    2.963363      103 31   37   41   42
   41  H    -5.293766   -0.383279    3.446654        6 40
   42  H    -4.848653   -2.002337    3.056108        6 40
   43  N     2.522558    3.113940   -1.156207      214 57   74
   44  N     3.383315   -2.836903   -1.824380      214 50   76
   45  N    -3.851663    0.420263   -3.635728      214 52   60
   46  N    -3.993954    3.289587   -1.409036      214 66   68
   47  N    -2.875923   -2.618750   -2.112670      214 55   62
   48  C     4.681525   -1.153086    0.213967      311 26   49   71
   49  C     4.519068   -0.799969   -1.136539      311 48   73   76
   50  C     2.418306   -3.029243   -2.621013      209 44   51   91
```

51	H	2.355766	-2.515698	-3.595390	10	50			
52	C	-4.940923	0.526503	-2.669725	103	29	45	53	54
53	H	-5.298117	1.554832	-2.745975	6	52			
54	H	-5.760746	-0.090472	-3.060093	6	52			
55	C	-2.570888	-3.838037	-1.977090	209	47	56	112	
56	H	-3.337351	-4.604336	-1.765828	10	55			
57	C	3.954927	2.968202	-0.895457	103	43	58	59	65
58	H	4.554026	3.303814	-1.752226	6	57			
59	H	4.201522	3.643899	-0.075619	6	57			
60	C	-2.686871	0.143736	-3.224443	209	45	61	106	
61	H	-2.453310	-0.024650	-2.166193	10	60			
62	C	-4.276344	-2.244460	-1.953948	103	30	47	63	64
63	H	-4.610127	-1.844553	-2.912295	6	62			
64	H	-4.917367	-3.115973	-1.741021	6	62			
65	C	4.283681	1.533663	-0.509282	311	57	72	73	
66	C	-2.790119	2.898493	-1.385170	209	46	67	100	
67	H	-2.456543	2.033587	-0.792961	10	66			
68	C	-4.991384	2.590452	-0.604362	103	6	46	69	70
69	H	-5.142524	3.209489	0.280276	6	68			
70	H	-5.933926	2.637567	-1.161659	6	68			
71	C	4.654757	-0.156244	1.204038	311	10	48	72	
72	C	4.395237	1.178921	0.845234	311	20	65	71	
73	C	4.401156	0.549463	-1.508311	311	49	65	82	
74	C	2.168195	3.679733	-2.234618	209	43	75	115	
75	H	2.918428	4.033672	-2.958602	10	74			
76	C	4.435018	-1.899889	-2.191545	103	44	49	77	78
77	H	5.378679	-2.459256	-2.229147	6	76			
78	H	4.274537	-1.480218	-3.191986	6	76			
79	N	3.282175	0.901967	-3.825324	214	80	82		
80	C	2.261930	0.267135	-3.422666	209	79	81	85	
81	H	2.215296	-0.205600	-2.431243	10	80			
82	C	4.470223	0.952130	-2.979205	103	73	79	83	84
83	H	5.223896	0.333354	-3.481725	6	82			
84	H	4.863869	1.966992	-3.065565	6	82			
85	C	1.035844	0.099680	-4.270024	3	80	86	87	124
86	H	1.071792	0.802600	-5.109100	6	85			
87	H	1.045737	-0.912999	-4.701723	6	85			
88	C	-1.059447	-0.145395	4.240781	3	38	89	90	121
89	H	-1.103999	0.851799	4.705569	6	88			
90	H	-1.081813	-0.871329	5.060928	6	88			
91	C	1.306150	-3.978493	-2.290576	3	50	92	93	130
92	H	1.306892	-4.780429	-3.044051	6	91			
93	H	1.531509	-4.436214	-1.321374	6	91			
94	C	-1.383999	3.981856	2.380432	3	8	95	96	127
95	H	-1.620789	4.480517	1.434164	6	94			
96	H	-1.375143	4.749255	3.168156	6	94			
97	C	1.662975	-3.539488	2.232439	3	24	98	99	136
98	H	1.979104	-4.551653	2.507577	6	97			
99	H	1.649520	-2.963149	3.170515	6	97			
100	C	-1.740503	3.563432	-2.237345	3	66	101	102	133
101	H	-1.733061	3.032394	-3.201492	6	100			
102	H	-2.050089	4.591032	-2.453934	6	100			
103	C	1.506517	-0.050325	4.170929	3	18	104	105	121
104	H	1.605793	-0.748860	5.009896	6	103			

105	H	1.532290	0.962352	4.597351	6	103			
106	C	-1.526187	-0.026563	-4.161376	3	60	107	108	124
107	H	-1.522090	-1.069836	-4.507336	6	106			
108	H	-1.660762	0.606966	-5.045039	6	106			
109	C	1.095543	4.357589	2.089133	3	13	110	111	127
110	H	0.907421	4.913291	1.158178	6	109			
111	H	1.065906	5.114118	2.887042	6	109			
112	C	-1.168569	-4.360077	-2.045796	3	55	113	114	130
113	H	-1.125213	-5.133170	-2.827915	6	112			
114	H	-0.988721	-4.889990	-1.099214	6	112			
115	C	0.741086	3.893784	-2.645274	3	74	116	117	133
116	H	0.601066	3.326293	-3.578381	6	115			
117	H	0.627522	4.946893	-2.939042	6	115			
118	C	-0.810408	-3.858735	2.666646	3	32	119	120	136
119	H	-0.713276	-4.901309	3.003472	6	118			
120	H	-0.648466	-3.256186	3.574275	6	118			
121	C	0.206727	-0.256221	3.405222	303	88	103	122	123
122	H	0.162788	0.477203	2.593068	603	121			
123	H	0.224786	-1.230418	2.907603	603	121			
124	C	-0.223704	0.264648	-3.428224	303	85	106	125	126
125	H	-0.163869	-0.397401	-2.556681	603	124			
126	H	-0.251916	1.282712	-3.026620	603	124			
127	C	-0.001016	3.320622	2.309082	303	94	109	128	129
128	H	0.205654	2.762091	3.230298	603	127			
129	H	0.010879	2.589516	1.493690	603	127			
130	C	-0.079038	-3.316179	-2.265936	303	91	112	131	132
131	H	-0.265033	-2.787359	-3.209764	603	130			
132	H	-0.126800	-2.567230	-1.468005	603	130			
133	C	-0.334489	3.505139	-1.632129	303	100	115	134	135
134	H	-0.147286	2.487426	-1.276535	603	133			
135	H	-0.269509	4.151509	-0.748354	603	133			
136	C	0.255257	-3.524602	1.623646	303	97	118	137	138
137	H	0.195589	-4.237106	0.792066	603	136			
138	H	0.039779	-2.543179	1.188074	603	136			
139	C	1.084472	0.635156	-0.076810	501	140	141		
140	N	0.514297	-0.367169	0.000479	502	139			
141	H	1.605178	1.584521	-0.198630	503	139			

NH_3@SP3

```
        142  molden generated tinker .xyz
   1  N     2.460248    3.117759    1.206599      214 15   32
   2  N     3.422090   -2.845780    1.751790      214  8   34
   3  N    -3.858143    0.260146    3.646185      214 10   18
   4  N    -4.047442    3.217504    1.464545      214 24   26
   5  N    -2.835675   -2.698079    2.028451      214 13   20
   6  C     4.700924   -1.101201   -0.241719      311  7   29   68
   7  C     4.529164   -0.784095    1.116043      311  6   31   34
   8  C     2.469039   -3.072239    2.553797      209  2    9   94
   9  H     2.415582   -2.593577    3.544689       10  8
  10  C    -4.941740    0.401948    2.677837      103  3   11   12   71
  11  H    -5.295178    1.428673    2.784297        6 10
  12  H    -5.765634   -0.224285    3.044761        6 10
  13  C    -2.514526   -3.910888    1.875899      209  5   14  109
  14  H    -3.270568   -4.685667    1.657327       10 13
  15  C     3.893431    2.976110    0.964741      103  1   16   17   23
  16  H     4.483928    3.288430    1.837119        6 15
  17  H     4.155220    3.674045    0.168395        6 15
  18  C    -2.685193    0.016462    3.235552      209  3   19  103
  19  H    -2.439760   -0.096508    2.172352       10 18
  20  C    -4.241256   -2.339631    1.883898      103  5   21   22   72
  21  H    -4.578130   -1.971891    2.853826        6 20
  22  H    -4.870543   -3.213472    1.646221        6 20
  23  C     4.238199    1.556473    0.546418      311 15   30   31
  24  C    -2.841949    2.830943    1.437824      209  4   25   97
  25  H    -2.507690    1.966360    0.846028       10 24
  26  C    -5.043657    2.515143    0.663516      103  4   27   28   48
  27  H    -5.229630    3.151291   -0.202239        6 26
  28  H    -5.976393    2.526451    1.239050        6 26
  29  C     4.656152   -0.080753   -1.207801      311  6   30   52
  30  C     4.367813    1.238900   -0.816702      311 23   29   62
  31  C     4.375156    0.552370    1.521921      311  7   23   40
  32  C     2.105647    3.641305    2.304884      209  1   33  118
  33  H     2.849900    3.952720    3.055960       10 32
  34  C     4.467408   -1.909969    2.142562      103  2    7   35   36
  35  H     5.417076   -2.460034    2.159548        6 34
  36  H     4.305378   -1.518834    3.153162        6 34
  37  N     3.289500    0.748171    3.883283      214 38   40
  38  C     2.236022    0.206564    3.429796      209 37   39   88
  39  H     2.129257   -0.105801    2.383062       10 38
  40  C     4.444764    0.907973    3.007558      103 31   37   41   42
  41  H     5.251784    0.321540    3.464514        6 40
  42  H     4.773213    1.942893    3.125052        6 40
  43  N    -2.510656   -3.153901   -1.215754      214 57   74
  44  N    -3.471560    2.823055   -1.781081      214 50   76
  45  N     3.854875   -0.276828   -3.650004      214 52   60
  46  N     4.004182   -3.186594   -1.549477      214 66   68
  47  N     2.766779    2.675986   -2.005412      214 55   62
  48  C    -4.747947    1.081532    0.235065      311 26   49   71
  49  C    -4.575551    0.757952   -1.119778      311 48   73   76
  50  C    -2.524198    3.049997   -2.589318      209 44   51   91
```

51	H	-2.465747	2.557459	-3.574636	10	50			
52	C	4.934237	-0.399412	-2.675661	103	29	45	53	54
53	H	5.310506	-1.417944	-2.784846	6	52			
54	H	5.747235	0.247508	-3.029827	6	52			
55	C	2.454291	3.896282	-1.889602	209	47	56	112	
56	H	3.218157	4.670077	-1.698687	10	55			
57	C	-3.940027	-3.004710	-0.956067	103	43	58	59	65
58	H	-4.544431	-3.314267	-1.821031	6	57			
59	H	-4.195733	-3.699787	-0.156042	6	57			
60	C	2.676463	-0.080320	-3.234866	209	45	61	106	
61	H	2.433046	0.011557	-2.169639	10	60			
62	C	4.177707	2.322577	-1.872363	103	30	47	63	64
63	H	4.503758	1.957637	-2.846955	6	62			
64	H	4.803045	3.201375	-1.644857	6	62			
65	C	-4.276774	-1.581164	-0.536689	311	57	72	73	
66	C	2.783311	-2.868479	-1.442558	209	46	67	100	
67	H	2.432723	-2.108804	-0.730738	10	66			
68	C	4.986879	-2.533180	-0.686844	103	6	46	69	70
69	H	5.118771	-3.191243	0.171854	6	68			
70	H	5.941098	-2.548928	-1.225412	6	68			
71	C	-4.689661	0.068168	1.208041	311	10	48	72	
72	C	-4.409686	-1.255707	0.824124	311	20	65	71	
73	C	-4.419363	-0.580949	-1.515138	311	49	65	82	
74	C	-2.170407	-3.588056	-2.355891	209	43	75	115	
75	H	-2.917603	-3.826143	-3.131346	10	74			
76	C	-4.514087	1.876672	-2.155975	103	44	49	77	78
77	H	-5.463510	2.426647	-2.179274	6	76			
78	H	-4.353035	1.478126	-3.164323	6	76			
79	N	-3.311059	-0.810746	-3.848243	214	80	82		
80	C	-2.273204	-0.246824	-3.393171	209	79	81	85	
81	H	-2.208290	0.116336	-2.355677	10	80			
82	C	-4.485531	-0.945180	-2.995300	103	73	79	83	84
83	H	-5.273139	-0.349666	-3.473139	6	82			
84	H	-4.827253	-1.975399	-3.112714	6	82			
85	C	-1.054722	-0.026097	-4.242121	3	80	86	87	124
86	H	-1.068081	-0.727949	-5.082725	6	85			
87	H	-1.098431	0.984876	-4.675151	6	85			
88	C	1.035661	-0.053649	4.292873	3	38	89	90	121
89	H	1.002931	-1.131792	4.513719	6	88			
90	H	1.145163	0.464719	5.251107	6	88			
91	C	-1.425710	4.015874	-2.252864	3	50	92	93	130
92	H	-1.425222	4.815334	-3.008173	6	91			
93	H	-1.665475	4.473419	-1.286954	6	91			
94	C	1.361199	-4.017193	2.199459	3	8	95	96	127
95	H	1.589152	-4.449980	1.219102	6	94			
96	H	1.363712	-4.838574	2.931355	6	94			
97	C	-1.790312	3.504824	2.277458	3	24	98	99	136
98	H	-2.075954	4.549905	2.438991	6	97			
99	H	-1.816153	3.022773	3.266324	6	97			
100	C	1.728849	-3.470626	-2.333210	3	66	101	102	133
101	H	1.721145	-2.876108	-3.259645	6	100			
102	H	2.038223	-4.479526	-2.626096	6	100			
103	C	-1.532745	-0.208012	4.168670	3	18	104	105	121
104	H	-1.751042	0.227022	5.149500	6	103			

105	H	-1.441185	-1.295335	4.304180	6	103				
106	C	1.508671	0.086621	-4.164696	3	60	107	108	124	
107	H	1.520175	1.113674	-4.554906	6	106				
108	H	1.614929	-0.583430	-5.025257	6	106				
109	C	-1.104258	-4.411596	1.931701	3	13	110	111	127	
110	H	-0.913391	-4.911307	0.970588	6	109				
111	H	-1.050800	-5.207700	2.689469	6	109				
112	C	1.048961	4.412272	-1.962663	3	55	113	114	130	
113	H	1.010508	5.192549	-2.737179	6	112				
114	H	0.854459	4.934344	-1.014183	6	112				
115	C	-0.742051	-3.788589	-2.773997	3	74	116	117	133	
116	H	-0.587676	-3.168769	-3.671072	6	115				
117	H	-0.628552	-4.823623	-3.127290	6	115				
118	C	0.676239	3.879964	2.689343	3	32	119	120	136	
119	H	0.551618	4.956933	2.876883	6	118				
120	H	0.527969	3.406613	3.671215	6	118				
121	C	-0.244857	0.330466	3.555062	303	88	103	122	123	
122	H	-0.165923	-0.022604	2.518737	603	121				
123	H	-0.302373	1.421056	3.495244	603	121				
124	C	0.210576	-0.156873	-3.407332	303	85	106	125	126	
125	H	0.165247	0.558463	-2.576886	603	124				
126	H	0.236191	-1.150584	-2.948759	603	124				
127	C	-0.027790	-3.362606	2.191711	303	94	109	128	129	
128	H	-0.220614	-2.869439	3.152132	603	127				
129	H	-0.077967	-2.579386	1.427113	603	127				
130	C	-0.034362	3.368600	-2.211640	303	91	112	131	132	
131	H	0.173376	2.844992	-3.153227	603	130				
132	H	0.002629	2.614265	-1.417528	603	130				
133	C	0.321378	-3.464647	-1.726184	303	100	115	134	135	
134	H	0.100274	-2.487060	-1.280122	603	133				
135	H	0.259787	-4.181769	-0.899426	603	133				
136	C	-0.376930	3.383243	1.702284	303	97	118	137	138	
137	H	-0.298563	3.943234	0.762615	603	136				
138	H	-0.167366	2.336742	1.454361	603	136				
139	N	1.043990	0.311797	0.249694	423	140	141	142		
140	H	1.364374	1.280529	0.203665	424	139				
141	H	0.346933	0.181071	-0.478563	424	139				
142	H	1.850779	-0.251449	-0.010333	424	139				

MeOH@SP3

```
       144  molden generated tinker .xyz
   1  N     2.337942   -3.160472   -1.285045     214 15   32
   2  N     3.485246    2.770402   -1.763162     214  8   34
   3  N    -3.922763   -0.167815   -3.599649     214 10   18
   4  N    -4.159303   -3.090079   -1.478340     214 24   26
   5  N    -2.722142    2.774851   -1.949807     214 13   20
   6  C     4.767017    0.960878    0.198504     311  7   29   68
   7  C     4.549508    0.668800   -1.157231     311  6   31   34
   8  C     2.535095    3.034970   -2.557451     209  2    9   94
   9  H     2.452531    2.557800   -3.548661      10  8
  10  C    -4.993970   -0.263652   -2.613209     103  3   11   12   71
  11  H    -5.402965   -1.269183   -2.724446       6 10
  12  H    -5.790844    0.410610   -2.954148       6 10
  13  C    -2.410996    3.999153   -1.879533     209  5   14  109
  14  H    -3.174814    4.780180   -1.718654      10 13
  15  C     3.774953   -3.066430   -1.045033     103  1   16   17   23
  16  H     4.353611   -3.382706   -1.924705       6 15
  17  H     4.018281   -3.784170   -0.261113       6 15
  18  C    -2.730435   -0.011254   -3.203203     209  3   19  103
  19  H    -2.454988    0.063769   -2.143564      10 18
  20  C    -4.132416    2.425112   -1.808770     103  5   21   22   72
  21  H    -4.465550    2.068800   -2.783881       6 20
  22  H    -4.752282    3.305756   -1.569933       6 20
  23  C     4.170569   -1.664840   -0.609103     311 15   30   31
  24  C    -2.939310   -2.748796   -1.426399     209  4   25   97
  25  H    -2.567162   -1.944111   -0.776479      10 24
  26  C    -5.121889   -2.398916   -0.625671     103  4   27   28   48
  27  H    -5.287709   -3.049832    0.232887       6 26
  28  H    -6.072688   -2.378817   -1.170925       6 26
  29  C     4.690407   -0.067125    1.155898     311  6   30   52
  30  C     4.337150   -1.368217    0.754895     311 23   29   62
  31  C     4.339790   -0.656291   -1.574306     311  7   23   40
  32  C     1.968976   -3.597239   -2.415877     209  1   33  118
  33  H     2.700869   -3.869120   -3.194454      10 32
  34  C     4.498509    1.805782   -2.172457     103  2    7   35   36
  35  H     5.460001    2.333590   -2.206337       6 34
  36  H     4.308207    1.426810   -3.183384       6 34
  37  N     3.224214   -0.860441   -3.915119     214 38   40
  38  C     2.208494   -0.251112   -3.467137     209 37   39   88
  39  H     2.169792    0.135692   -2.439554      10 38
  40  C     4.397356   -1.004733   -3.061095     103 31   37   41   42
  41  H     5.188721   -0.408428   -3.531904       6 40
  42  H     4.736969   -2.035159   -3.184058       6 40
  43  N    -2.282460    3.080890    1.292710     214 57   74
  44  N    -3.549047   -2.795182    1.803180     214 50   76
  45  N     3.946956    0.163101    3.624940     214 52   60
  46  N     4.195929    3.082165    1.511755     214 66   68
  47  N     2.717935   -2.779971    1.945671     214 55   62
  48  C    -4.775752   -0.982245   -0.181954     311 26   49   71
  49  C    -4.564973   -0.681151    1.172142     311 48   73   76
  50  C    -2.571797   -3.023337    2.574016     209 44   51   91
```

51	H	-2.461417	-2.510810	3.544198	10	50			
52	C	5.008346	0.227507	2.622716	103	29	45	53	54
53	H	5.443669	1.222540	2.726023	6	52			
54	H	5.791338	-0.463987	2.960693	6	52			
55	C	2.367137	-3.985429	1.793567	209	47	56	112	
56	H	3.105121	-4.775130	1.567733	10	55			
57	C	-3.718750	3.031430	1.018425	103	43	58	59	65
58	H	-4.302845	3.388294	1.878260	6	57			
59	H	-3.914124	3.742638	0.216196	6	57			
60	C	2.761931	-0.071351	3.247977	209	45	61	106	
61	H	2.506283	-0.233976	2.195874	10	60			
62	C	4.134597	-2.462215	1.797838	103	30	47	63	64
63	H	4.487176	-2.120482	2.771764	6	62			
64	H	4.734258	-3.352384	1.545332	6	62			
65	C	-4.149691	1.638642	0.600315	311	57	72	73	
66	C	2.975841	2.745155	1.486339	209	46	67	100	
67	H	2.605012	1.933394	0.847077	10	66			
68	C	5.132561	2.374058	0.643505	103	6	46	69	70
69	H	5.294015	3.025237	-0.215971	6	68			
70	H	6.092327	2.341317	1.171753	6	68			
71	C	-4.689598	0.035963	-1.147251	311	10	48	72	
72	C	-4.327171	1.336713	-0.759397	311	20	65	71	
73	C	-4.330623	0.643002	1.575300	311	49	65	82	
74	C	-1.932359	3.616508	2.387642	209	43	75	115	
75	H	-2.681935	3.983819	3.106704	10	74			
76	C	-4.544698	-1.808833	2.198993	103	44	49	77	78
77	H	-5.518440	-2.314191	2.228797	6	76			
78	H	-4.352796	-1.423646	3.207202	6	76			
79	N	-3.198466	0.810442	3.907257	214	80	82		
80	C	-2.170181	0.239532	3.436077	209	79	81	85	
81	H	-2.095869	-0.084137	2.386201	10	80			
82	C	-4.365210	1.005726	3.057374	103	73	79	83	84
83	H	-5.181776	0.450944	3.535545	6	82			
84	H	-4.654445	2.052456	3.177731	6	82			
85	C	-0.964890	-0.030421	4.293481	3	80	86	87	124
86	H	-0.974413	0.644611	5.155956	6	85			
87	H	-1.032361	-1.053845	4.693179	6	85			
88	C	0.980905	-0.013110	-4.297622	3	38	89	90	121
89	H	1.036299	0.995810	-4.735246	6	88			
90	H	0.968504	-0.718233	-5.135864	6	88			
91	C	-1.504620	-4.011869	2.210558	3	50	92	93	130
92	H	-1.506184	-4.815831	2.961512	6	91			
93	H	-1.773976	-4.458282	1.247225	6	91			
94	C	1.468899	4.028852	-2.201335	3	8	95	96	127
95	H	1.716123	4.453057	-1.222232	6	94			
96	H	1.505768	4.847225	-2.935454	6	94			
97	C	-1.921849	-3.388010	-2.332393	3	24	98	99	136
98	H	-2.266773	-4.387846	-2.616676	6	97			
99	H	-1.909951	-2.793200	-3.258807	6	97			
100	C	1.956559	3.388567	2.384330	3	66	101	102	133
101	H	1.931784	2.794347	3.310688	6	100			
102	H	2.301000	4.388081	2.669471	6	100			
103	C	-1.583644	0.118993	-4.168370	3	18	104	105	121
104	H	-1.720980	-0.571436	-5.008684	6	103			

105	H	-1.595079	1.135115	-4.587233	6	103			
106	C	1.606956	-0.203271	4.200307	3	60	107	108	124
107	H	1.585218	-1.238265	4.569873	6	106			
108	H	1.757641	0.445902	5.069967	6	106			
109	C	-1.001319	4.502312	-1.962865	3	13	110	111	127
110	H	-0.795720	5.034922	-1.022127	6	109			
111	H	-0.951044	5.268854	-2.749924	6	109			
112	C	0.948593	-4.460089	1.865004	3	55	113	114	130
113	H	0.892579	-5.258077	2.620595	6	112			
114	H	0.733879	-4.952311	0.905270	6	112			
115	C	-0.506554	3.783789	2.819649	3	74	116	117	133
116	H	-0.375017	3.169891	3.724321	6	115			
117	H	-0.372014	4.819872	3.160576	6	115			
118	C	0.531849	-3.761131	-2.812618	3	32	119	120	136
119	H	0.390026	-4.792504	-3.166745	6	118			
120	H	0.378765	-3.136021	-3.706112	6	118			
121	C	-0.271024	-0.118505	-3.435912	303	88	103	122	123
122	H	-0.212102	0.603739	-2.615722	603	121			
123	H	-0.294595	-1.100511	-2.953645	603	121			
124	C	0.305639	0.098530	3.466696	303	85	106	125	126
125	H	0.228070	-0.580209	2.610145	603	124			
126	H	0.352439	1.108364	3.044786	603	124			
127	C	0.056528	3.430542	-2.193083	303	94	109	128	129
128	H	-0.147662	2.913951	-3.139304	603	127			
129	H	-0.030197	2.676570	-1.404303	603	127			
130	C	-0.102065	-3.391970	2.145974	303	91	112	131	132
131	H	0.135175	-2.887699	3.091360	603	130			
132	H	-0.067069	-2.626300	1.362711	603	130			
133	C	0.549460	3.421053	1.781083	303	100	115	134	135
134	H	0.303350	2.442715	1.356302	603	133			
135	H	0.506202	4.127352	0.942843	603	133			
136	C	-0.507091	-3.415083	-1.748746	303	97	118	137	138
137	H	-0.447702	-4.132786	-0.921884	603	136			
138	H	-0.271665	-2.439034	-1.310733	603	136			
139	O	-0.861413	0.143355	-0.007523	38	140	141		
140	H	-1.193879	1.041076	0.156133	39	139			
141	C	0.550110	0.153480	-0.072979	41	139	142	143	144
142	H	0.883278	-0.844294	-0.373399	40	141			
143	H	0.936948	0.882167	-0.798243	40	141			
144	H	1.007650	0.377047	0.895442	40	141			
